# Supplementary material for: Neoadjuvant PD-1 blockade in surgically resectable desmoplastic melanoma: cohort A of the phase 2 SWOG S1512 trial
Source: Nat Cancer. 2026 Jan 29;7(2):272–82. doi: 10.1038/s43018-025-01113-y (PMC12948668; doi:10.1038/s43018-025-01113-y)
Supplement: Supplementary file 1 — Supplementary Tables 1–8 and clinical trial protocol. [file 43018_2025_1113_MOESM1_ESM.pdf]

nature cancer

Supplementary information

https://doi.org/10.1038/s43018-025-01113-y

Neoadjuvant PD-1 blockade in surgically resectable desmoplastic melanoma: cohort A of the phase 2 SWOG S1512 trial

In the format provided by the authors and unedited

Supplementary Tables

Neoadjuvant PD-1 blockade in surgically resectable desmoplastic melanoma: cohort A of the phase 2 SWOG S1512 trial.

Kendra et al.

Index:

Supplementary Table 1. Individual patient baseline characteristics.

Supplementary Table 2. Individual patient treatment summary and outcomes.

Supplementary Table 3. Clinical response rate. Objective response rate (ORR) after 9 weeks of neoadjuvant pembrolizumab, assessed radiologically or clinically.

Supplementary Table 4. Surgery and pathology analysis of patients with involved lymph nodes at baseline.

Supplementary Table 5. Centralized pathology review of diagnostic biopsies, on-therapy biopsies and surgical resection specimens.

Supplementary Table 6. Summary data from the centralized pathology review of diagnostic biopsies, on-therapy biopsies and surgical resection specimens.

Supplementary Table 7. Treatment-related toxicities observed in ≥ 5% of patients who received protocol therapy.

Supplementary Table 8. Genomic summary of samples and genes of interest.

Supplementary Table 1. Individual patient baseline characteristics.

ID	Age	Sex	PS	Target Lesions	Non-target Lesions	AJCC Stage	Disease Status
PT0209	>40-50	F	0	Right proximal lateral arm		T2aN0M0	Primary
PT0216	>80-90	M	1	Left scalp soft tissue		T3aN0M0	Recurrent
PT0289	>70-80	M	0	Left lower back		T4aN2cM0	Recurrent
PT0502	>80-90	M	0	Scalp lesion		T3aN0M0	Primary
PT0526	>80-90	M	0	Scalp lesion		T4bN0M0	Primary
PT0560	>80-90	M	0	NA	Right submandibular lymph node	T4aN1bM0	Recurrent
PT0634	>60-70	M	1	Right cheek lesion		T4aN0M0	Primary
PT0644	>80-90	F	0	Left nose, cutaneous		T4aN0M0	Primary
PT0654	>80-90	M	0	Central forehead, adjacent to frontal scalp		T2aN0M0	Primary
PT0665	>90	F	0	Left nose ridge		T1bN0M0	Primary

PT0674	>40-50	F	0	Mid mucosa of upper lip, cutaneous		T2aN0M0	Recurrent
PT0697	>70-80	M	1	Left upper back subcutaneous nodule		T1aN0M0	Primary
PT0698	>30-40	M	0	Right upper back	Right axillary lymph node-	TXN0M0	Primary
PT0707	>70-80	M	0	Right posterior shoulder, right axillary lymph node		T2aN1bM0	Primary
PT0760	>70-80	M	0	Left parietal scalp		T4aN0M0	Primary
PT0761	>60-70	M	0	Left plantar foot mass		T4bN0M0	Primary
PT0764	>80-90	M	0	Right parietal scalp		T4aN0M0	Primary
PT0771	>50-60	M	0	Left axillary lymph node		T3aN2cM0	Primary
PT0773	>80-90	M	0	Right parietal scalp		T3aN0M0	Primary
PT0776	>70-80	M	0	Bridge of nose		T3aN0M0	Primary
PT0779	>70-80	M	1	Left frontal scalp		T4aN0M0	Primary
PT0784	>50-60	M	0	NA	Left lip	T3aN0M0	Recurrent
PT0785	>50-60	M	1	Right axillary lymph node, right back subcutaneous nodules	Additional right axillary lymph nodes	T3bN1bM0	Primary

PT0786	>70-80	F	0	NA	Right cheek	T4aN0M0	Primary
PT0788	>80-90	M	1	Left superior posterior subcutaneous nodule		T2aN0M0	Primary
PT0791	>70-80	F	0	Left posterior proximal upper arm		T1bN0M0	Primary
PT0798	>70-80	F	0	Right forearm		T2aN0M0	Primary
PT0806	>70-80	M	0	Scalp Mass		T3aN0M0	Primary

NA, not evaluable due to not having measurable disease at baseline.

Supplementary Table 2. Individual patient treatment summary and outcomes.

ID	Number of Infusions	Reason for Discontinuation	Clinical Response[1]	pCR	Relapse	RFS (months)	OS (months)	Cause of Death
PT0209	3	Treatment complete	uPR	Yes	No	58	61	Alive
PT0216	3	Treatment complete	PD	No	No	0[2]	2	Alive
PT0289	3	Treatment complete	SD	Yes	No	61	64	Alive
PT0502	4	Treatment complete	uPR	Yes	No	53	57	Alive
PT0526	3	Treatment complete	uCR	Yes	No	29	32	Unknown
PT0560	3	Treatment complete	NA	Yes	No	31	37	Other – Cardiac related
PT0634	3	Treatment complete	uPR	Yes	No	56	58	Alive
PT0644	3	Treatment complete	NA	Yes	No	46	48	Alive
PT0654	3	Treatment complete	SD	Yes	No	51	54	Alive
PT0665	3	Treatment complete	uPR	No	No	24	26	Alive

PT0674	3	Treatment complete	SD	No	No	53	55	Alive
PT0697	3	Treatment complete	NA	Yes	No	47	50	Alive
PT0698	3	Treatment complete	NA	Yes	No	50	53	Alive
PT0707	3	Treatment complete	uPR	Yes	No	45	48	Alive
PT0760	3	Treatment complete	uPR	Yes	No	46	49	Alive
PT0761	3	Treatment complete	SD	No	Locally recurrent	23	39	Alive
PT0764	3	Treatment complete	SD	No	Locally and distant recurrent	6	21	Other – Sepsis among multiple factors
PT0771	3	Treatment complete	uPR	Yes	No	32	35	Alive
PT0773	3	Treatment complete	uCR	Yes	No	40	42	Alive
PT0776	3	Treatment complete	uCR	Yes	No	40	42	Alive
PT0779	3	Treatment complete	SD	Yes	No	27	30	Other – Cardiac related
PT0784	3	Treatment complete	NA	No[3]	No	NA[3]	10	Alive

PT0785	4	Treatment complete	uPR	No	No	16	20	Alive
PT0786	3	Treatment complete	NA	Yes	No	26	29	Alive
PT0788	3	Treatment complete	SD	Yes	No	36	39	Alive
PT0791	1	Adverse event: Immune-mediated colitis	SD	Yes	No	33	36	Alive
PT0798	3	Treatment complete	SD	No	Locally recurrent	30	33	Alive
PT0806	3	Treatment complete	uCR	Yes	No	27	30	Alive

[1]Clinical response at 9-week assessment, prior to resection. uCR, unconfirmed complete response. uPR, unconfirmed partial response. SD, stable disease. PD, progressive disease. NA, not evaluable due to: not having measurable disease at baseline (3), not assessed using the same technique as baseline (2), no assessment prior to resection (1).

[2]Withdrew consent after undergoing resection.

[3]Did not undergo resection.

Supplementary Table 3. Clinical response rate. Objective response rate (ORR) after 9 weeks of neoadjuvant pembrolizumab, assessed radiologically or clinically.

**9-week response**[1]	**N**	**%**
Complete Response	4	16
Partial Response	8	32
Stable Disease	9	36
Progressive Disease	1	4
Not evaluable[2]	3	12
**Total**[3]	**25**	**100**
**Overall Response Rate (ORR)**	**12**	**48%**
**95% Confidence Interval**		**(28%-69%)**

[1] Response assessed radiographically or by clinical exam with photos.

[2] One patient did not have a 9-week assessment prior to surgery and two patients were not assessed using the same technique as baseline.

[3] Among patients with measurable disease at baseline.

Supplementary Table 4. Surgery and pathology analysis of patients with involved lymph nodes at baseline.

ID	Lesion Locations	AJCC Stage	Surgery and pathology assessment
PT0289	Left lower back	T4aN2cM0	Wide excision and left axillary sentinel lymph node resection<br>0/5 lymph nodes
PT0560	Right submandibular lymph node	T4aN1bM0	Right parotid and right neck lymphadenectomy<br>0/20 lymph nodes
PT0707	Right posterior shoulder, right axillary lymph node	T2aN1bM0	Wide excision and right axillary lymphadenectomy<br>0/20 lymph nodes
PT0771	Left axillary lymph node	T3aN2cM0	Wide excision and left axillary lymphadenectomy<br>0/36 lymph nodes
PT0785	Right axillary lymph node, right back subcutaneous nodule	T3bN1bM0	Wide excision and right axillary lymphadenectomy<br>2/30 positive lymph nodes

Supplementary Table 5. Centralized pathology review of diagnostic biopsies, on-therapy biopsies and surgical resection specimens.

ID	Diagnostic pathology[a]	Desmoplastic histology[b]	On-therapy biopsy[c]	Surgical resection[d]
PT0209	Biopsy not evaluable	N/A	Biopsy not evaluable (of two blocks submitted)	Surgical specimen not available
PT0216	Confirmed desmoplastic melanoma	Mixed	No pathological response	No pathological response
PT0289	Not confirmed desmoplastic melanoma	N/A	No pathological response (consistent with desmoplastic melanoma)	pCR
PT0502	Confirmed desmoplastic melanoma	Pure	No pathological response	pCR
PT0526	Confirmed desmoplastic melanoma	Pure	Biopsy not available	pCR
PT0560	Confirmed desmoplastic melanoma	Pure	Some areas of pathological response	pCR
PT0634	Confirmed desmoplastic melanoma	Pure	No pathological response	pCR
PT0644	Confirmed desmoplastic melanoma	Pure	No pathological response	pCR
PT0654	Confirmed desmoplastic melanoma	Mixed	Biopsy not available	pCR
PT0665	Confirmed desmoplastic melanoma	Pure	No pathological response	No pathological response (consistent with desmoplastic melanoma)
PT0674	Biopsy not evaluable	N/A	Biopsy not evaluable (of one block submitted)	No pathological response (consistent with desmoplastic melanoma)
PT0697	Confirmed desmoplastic melanoma	Pure	Biopsy not available	pCR
PT0698	Confirmed desmoplastic melanoma	Pure	Biopsy not evaluable (of three blocks submitted)	pCR

PT0707	Confirmed desmoplastic melanoma	Mixed	Entirely pathological tumor response (no viable tumor)	pCR
PT0760	Confirmed desmoplastic melanoma	Mixed	Biopsy not evaluable (of three blocks submitted)	pCR
PT0761	Confirmed desmoplastic melanoma	Mixed	No pathological response	pPR with near pCR
PT0764	Confirmed desmoplastic melanoma	Mixed	Some areas of pathological response	No pathological response (consistent with desmoplastic melanoma)
PT0771	Not confirmed desmoplastic melanoma	N/A	Entirely pathological tumor response (no viable tumor)	pCR
PT0773	Confirmed desmoplastic melanoma	Pure	Entirely pathological tumor response (no viable tumor)	pCR
PT0776	Biopsy not evaluable	N/A	Entirely pathological tumor response (no viable tumor)	pCR
PT0779	Confirmed desmoplastic melanoma	Mixed	No pathological response	pCR
PT0784	Biopsy not evaluable	N/A	Biopsy not available	Surgery not performed
PT0785	Confirmed desmoplastic melanoma	Mixed	Entirely pathological tumor response (no viable tumor)	Surgical specimen not available
PT0786	Confirmed desmoplastic melanoma	Pure	Biopsy not evaluable (of three blocks submitted)	pCR
PT0788	Confirmed desmoplastic melanoma	Pure	No pathological response	pCR
PT0791	Confirmed desmoplastic melanoma	Pure	Entirely pathological tumor response (no viable tumor)	Surgical specimen not available
PT0798	Biopsy not evaluable	N/A	Biopsy not available	No pathological response (consistent with desmoplastic melanoma)
PT0806	Confirmed desmoplastic melanoma	Mixed	Entirely pathological tumor response (no viable tumor)	pPR

Legend. [a]Biopsy of the primary lesion or the S1512 clinical trial baseline biopsy were reviewed centrally to confirm the diagnosis of desmoplastic melanoma. Two pathologists (D.W., J.A.P.) reviewed the available stained slides (including H&E and immunohistochemistry for either S100 or SOX10 [melanoma cell markers]), resulting in a consensus determination of whether the biopsies were consistent with the diagnosis of desmoplastic melanoma. Consensus annotation was used to describe whether there appeared to be tumor content in the diagnostic biopsies and if the two pathologists agreed that the tumor region of these biopsies was consistent with desmoplastic melanoma pathology. [b]Diagnostic biopsies were annotated for their degree of desmoplastic histology. Disease was annotated as "Pure" if at least 90% of the tumor content contained spindled tumor cells with abundant stromal collagen (desmoplasia) imparting a scar-like pattern. "Mixed" histology indicates the presence of desmoplastic histology and a significant component (greater than 10% of tumor area) in the diagnostic biopsy with a high cell density, lacking stromal collagen, and/or presence of epithelioid tumor cells. This value is not available (N/A) if the diagnostic biopsy was not confirmed as desmoplastic melanoma or the sample not available. [c]On-therapy biopsies were collected after one cycle of therapy (between days 28 and 35 after the start of treatment). Slides submitted for central review were analyzed to determine whether there was tumor content and if the two pathologists agreed that the tumor region was consistent with desmoplastic melanoma pathology (consistent with the centralized pathology review of the diagnostic biopsies). In addition, slides were reviewed for a consensus on whether there were features of pathological response by the tumor, including the presence of treatment effect (fibrosis or melanosis) and whether there were viable tumor cells. [d]Clinical study sites were requested to provide specimens from the surgical excision after three infusions of neoadjuvant pembrolizumab, which were stored as formalin-fixed, paraffin-embedded blocks, slides (unstained or H&E-stained), or digital images (from stained slides). Slides were reviewed to assess whether there was tumor content, if it was consistent with desmoplastic melanoma pathology, and the

extent of pathological response. Biopsy or specimen not available: the sample was not provided for central review. Biopsy or specimen not evaluable: the provided sample did not contain tumor cells or areas that were consistent with a regressed tumor. No pathological response: indicates that there were only viable tumor cells and an absence of a treatment effect by pathological review. pPR: partial pathological response with viable tumor cells remaining in the evaluated surgical specimens. pCR: pathological complete response with no viable tumor cells and the entire resection contained treatment effect with evidence of pathological response.

Supplementary Table 6. Summary data from the centralized pathology review of diagnostic biopsies, on-therapy biopsies and surgical resection specimens.

Timing	Assessment	Number	Percentage of the evaluable samples
Diagnostic biopsies	Biopsy not available	2	-
	Biopsy not evaluable	3	-
	Not confirmed to be desmoplastic melanoma	2	9%
	Confirmed to be desmoplastic melanoma	21	91%
On-therapy biopsies	Biopsy not available	5	-
	Biopsy not evaluable	5	-
	No pathological response	9	50%
	Some areas of pathological response	2	11%
	Entirely pathological tumor response (no viable tumor)	7	39%
Surgical specimens	Specimen not available	4	-
	Specimen not evaluable	0	-
	No pathological response	5	21%
	pPR	2	8%

	pCR	17	71%

Legend. Biopsy or specimen not available: the sample was not provided for central review. Biopsy or specimen not evaluable: the provided sample did not contain tumor cells or areas that were consistent with a regressed tumor. No pathological response: indicates that there were only viable tumor cells and an absence of a treatment effect by pathological review. Some areas of pathological response: indicates that the on-therapy biopsy had residual cancer cells and areas consistent with an antitumor immune response. Entirely pathological tumor response (no viable tumor): indicates that the sampled area in the on-therapy biopsy was consistent with having no residual cancer cells after an immune response. pPR: partial pathological response with viable tumor cells remaining in the evaluated surgical specimens. pCR: pathological complete response with no viable tumor cells and the entire resection contained treatment effect with evidence of pathological response.

Supplementary Table 7. Treatment-related toxicities observed in ≥ 5% of patients who received protocol therapy.

	Pembrolizumab (n=28)	
**Adverse Event**	**Any grade**	**Grade 3-5**
Fatigue	12 (43%)	0 (0%)
Maculopapular rash	6 (21%)	0 (0%)
Diarrhea	4 (14%)	0 (0%)
Arthralgia	2 (7%)	0 (0%)
Headache	2 (7%)	0 (0%)
Lymphocyte count decreased	2 (7%)	0 (0%)
Myalgia	2 (7%)	0 (0%)

Legend. All adverse events reported were assessed as possibly, probably or definitely related to the study treatment. Adverse event severity was scored using the National Cancer Institute Common Terminology Criteria for Adverse Events v4.0.

Supplementary Table 8. Genomic summary of samples and genes of interest.

ID	Tumor purity[a]	Average sequencing depth[b]	TMB[c]	Mutations in genes of interest[d]
PT0216	0.19	197.73	158.36	ROS1 L30fs (14% VAF), TP53 L194F (16% VAF)
PT0289	0.73	184.27	72.40	NF1 Y1521* (43% VAF)
PT0502	1	202.83	59.60	NF1 K261* (7% VAF), ROS1 E2265K (11% VAF), TP53 I255fs (16% VAF), CDKN2A P48L (6% VAF), BRAF N581S (6% VAF)
PT0526	0.28	197.55	90.20	NF1 Q1218* (14% VAF), ROS1 L1949F (8% VAF), TP53 P151S (17% VAF), CDKN2A W110* (14% VAF)
PT0560	0.27	167.60	109.96	NF1 splice_acceptor (13% VAF), ROS1 P944S (18% VAF), TP53 R110P (20% VAF), CDKN2A G55fs (17% VAF)
PT0634	0.99	195.77	49.65	NF1 Q948* (14% VAF), ROS1 K2228Q (28% VAF), TP53 R342* (13% VAF)
PT0644	0.24	194.44	96.40	NF1 R1241* (11% VAF), ROS1 G227D (7% VAF), TP53 R342* (10% VAF), CDKN2A P114L (12% VAF)
PT0654	0.36	176.94	25.01	No recurrent drivers detected
PT0665	0.62	185.63	24.32	NF1 Q1010* (5% VAF), ROS1 S819F (6% VAF), TP53 A406T (7% VAF)
PT0697	0.94	188.50	60.69	NF1 Q2239* (7% VAF), ROS1 G1382E (7% VAF)
PT0761	0.7	194.10	0.43	BRAF V600E (7% VAF)
PT0764	0.45	175.97	35.46	NF1 R1362* (26% VAF), ROS1 E1935K (23% VAF), TP53 L111Q (17% VAF)
PT0773	0.25	179.88	44.60	ROS1 M1527I (5% VAF)
PT0779	0.49	198.01	160.13	NF1 H1494Y (22% VAF), ROS1 R1802G (15% VAF), TP53 R196* (23% VAF), CDKN2A P114L (32% VAF)
PT0785	0.35	170.51	85.93	NF1 S1599F (41% VAF), ROS1 W1397* (27% VAF), TP53 R213* (53% VAF), CDKN2A R58* (31% VAF)
PT0788	0.47	198.63	64.86	NF1 L161* (13% VAF), TP53 K164* (49% VAF), CDKN2A P48L (33% VAF)

Legend: WES samples were filtered to represent the driver genetic events corresponding to each patient, based upon biopsies with sufficient tumor content that was annotated for desmoplastic melanoma. [a]Tumor cellularity was summarized by Sequenza based upon

the copy number profile. [b]The average depth of sequencing indicates the average number of reads at each genomic position throughout the whole exome (defined by the targeted regions of the Agilent SureSelect Exome v7). [c]Tumor mutational burden (TMB, in mutations per megabase) was calculated for each sample. [d]Putative genetic drivers are shown for each patient, including mutations in *NF1, ROS1, TP53, CDKN2A,* and *BRAF.* The mutation impact on protein sequence and variant allele frequency (VAF) is shown for each mutation.

SWOG CANCER RESEARCH NETWORK

Version Date: January 30, 2023

TO: ALL NATIONAL CLINICAL TRIALS NETWORK (NCTN) MEMBERS; CTSU

FROM: SWOG Operations Office (protocols@swog.org)

RE: **S1512**, "A Phase II and Pilot Trial of PD-1 Blockade with Pembrolizumab (MK-3475) in Patients with Resectable or Unresectable Desmoplastic Melanoma (DM)". Study Chairs: Drs. K. Kendra, S. Hu-Lieskovan, W. E. Carson III

REVISION #10

Study Chair: Kari Kendra, M.D., Ph.D.
Phone number: : 614/293-4320
E-mail: kari.kendra@osumc.edu

Action Codes

(✓) Patients Must be Informed*

(✓) Consent Must Be Amended*

* See "Patient Notification and Use of Consent Addendum" and "Regulatory Considerations" instructions below.

Key Updates

(√) Dose modification updates
(√) Other: CAEPR Update for Pembrolizumab
(√) Informed Consent Changes

Sites using the CIRB as their IRB of record: The protocol and/or informed consent form changes have been approved by the CIRB and must be activated within 30 days of distribution of this notice through the CTSU Bi-Monthly Broadcast email

Sites not using the NCI CIRB: Per CTMB Guidelines, the protocol updates and/or informed consent changes must be approved by local IRBs within 90 days of distribution of this notice through the CTSU Bi-Monthly Broadcast email.

REVISION #10

S1512 has been revised with the following changes in response to the Request for Rapid Amendment (RRA) received on February 03, 2023 from Dr. Elad Sharon (sharone@mail.nih.gov). The associated Action Letter is attached.

1. Throughout the protocol and consent, formatting has been updated as needed.

Protocol Changes:

1. The version date has been updated.

2. The table of contents has been updated.

3. **Section 3.1.c**: The pembrolizumab (MK-3475) CAEPR has been updated to Version 2.7, December 13, 2022 as follows:

Added New Risk:

a. Rare but Serious: Endocrine disorders - Other (hypoparathyroidism); Nervous system disorders - Other (optic neuritis)

Decrease in Risk Attribution:

a. Changed to Also Reported on pembrolizumab (MK-3475) Trials But With Insufficient Evidence for Attribution from Less Likely: CPK increased; Joint effusion; Pleuritic pain

Deleted:

b. Less Likely: Immune system disorders - Other (pseudoprogression/tumor inflammation); Infection; Musculoskeletal and connective tissue disorder – Avascular Necrosis; Musculoskeletal and connective tissue disorder - Other (tenosynovitis)

c. Footnote #4 "Infection includes all 75 sites of infection under the INFECTIONS AND INFESTATIONS SOC." is deleted.

4. **Section 7.1:** A section regarding supportive care for pembrolizumab has been added. Subsequent sections have been renumbered.

5. **Section 8.1:** This section has been updated to reference CTCAE Criteria version 5.0 for consistency with the updated pembrolizumab dose modification guidelines provided by the NCI.

6. **Section 8.2:** A new section for "General Considerations" has been added. Information regarding missed doses and dose delays has been moved to this section. A statement regarding supportive care information has been added to cross reference Sections 7.1 and 8.3a. Subsequent sections have been renumbered.

7. **Section 8.3:** The section title has been updated. Additional information has been added regarding identification and management of immune-related AEs (irAEs), infusion-reactions, and non-irAEs.

8. **Section 8.3a**: The section title has been updated.

9. **Section 8.3a, Table 1:** The table has been revised in the following ways:

General instructions

- Instruction 1 has been changed to include non-endocrine-related severe and life-threatening irAEs. A statement has been added to highlight that some non-endocrine irAEs do not require steroids.
- Instruction 2 has been revised to specifically mention non-endocrine-related toxicities.
- Instruction 3 has been reworded for clarity.
- Instruction 4 has been revised to clarify instructions for non-endocrine irAE.

irAEs Toxicity Management Guidelines

- Diarrhea / Colitis:
  - In the "Corticosteroid and/or other therapy" section, a sentence has been added to clarify that a gastroenterologist should examine patients who do not respond to corticosteroids in order to confirm the diagnosis and assess secondary immune suppression.
  - The section "Monitoring and Follow-up" has been updated to include a statement that participants should specifically be evaluated for celiac disease serologically and rule out Clostridium difficile infections. Additionally, wording has been added for assessing mucosal severity for participants with ≥Grade 2.

- Type 1 diabetes mellitus (T1DM) or Hyperglycemia:
  - New guidelines for "Grade 1 or 2" have been added.

    - "New Onset T1DM or Grade 3 or 4 hyperglycemia associated with evidence of B-cell failure" has been updated to "New onset T1DM (evidence of B-cell failure) or Grade 3 or 4 hyperglycemia" and the following have been added: a clarification for resuming pembrolizumab and updates to management, monitoring, and follow-up. Additionally, wording has been added for assessing mucosal severity for participants with ≥Grade 2.

- Hypophysitis:
    - Under the column "Monitoring and follow-up," a statement has been added to instruct patients are to be provided with adrenal insufficiency precautions, including indications for stress dosage steroids and medical alert jewelry, and that they should consider referral to an endocrinologist.

- Hyperthyroidism:
    - For Grade 2, in the column "Action with pembrolizumab", "continue" has been updated to "Consider withholding. Resume pembrolizumab once symptoms have subsided and thyroid function has improved".
    - A sentence has been added under the column "Corticosteroid and/or other medications" to initiate treatment with an anti-thyroid medicine such as methimazole or carbimazole as needed.
    - A sentence has been inserted under the column "Monitoring and follow-up" stating that patients should strongly consider referral to an endocrinologist.

- Nephritis:
    - In the "Monitoring and Follow-up" section, a statement has been added advising patients to strongly consider referral to a nephrologist.

- The "Myocarditis" section has been updated to "Cardiac Events (including myocarditis, pericarditis, arrhythmias, impaired ventricular function, vasculitis)"
    - Grade 1 has been clarified as "Asymptomatic cardiac enzyme elevation with clinical suspicion of myocarditis (previously CTCAE v4.0 Grade 1), or Grade 1" and now has separate guidelines for management, monitoring, and follow-up.
    - The guidelines for Grade 2 have been combined with Grades 3-4. Additional guidelines for management, monitoring, and follow-up have been added.

- Guidelines for"Exfoliative Dermatologic Conditions" have been added.

- The footnotes have been updated as follows:
    - Added definitions for acronyms used within the table.
    - Added a note about managing non-irAEs following clinical practice recommendations.
    - The sentence referring to resuscitation equipment and physician availability has also been included here.

10. **Section 8.3b, Table 2:** The table title has been revised to "Pembrolizumab (MK-3475) Infusion Reaction Treatment Guidelines"
    - An additional column has been added for "Infusion Reactions" description as it applies to each NCI CTCAE Grade.
    - Throughout the table "patient(s)" has been updated to "participant(s)"
    - The description for "Infusion Reactions" and "Treatment" for Grade 2 has been reworded for clarity.

    - Under grade 3 treatment:

- Corticosteroids has been clarified to include the following additional information " (*e.g.* methylprednisolone 2 mg/kg/day or dexamethasone 10 mg every 6 hours)"
- A statement has been added that epinephrine should be used immediately in situations of anaphylaxis.
- "Grade 4" guidelines have been separated from "Grade 3". Instructions have been added to admit participants to the intensive care unit (ICU) with monitoring and additional treatment and, as appropriate, follow Grade 3 recommendations.

11. **Section 8.3c, Table 3:** The "Management of Neurological Toxicities" section and "Neurological Toxcities" table have been added.

Model Consent Form Changes

1. The version date has been updated.

2. The pembrolizumab (MK-3475) risk profile date has been updated to CAEPR Version 2.7, December 13, 2022. Instances of MK-3475 (pembrolizumab) have been updated to pembrolizumab (MK-3475).

3. Possible Side effects of pembrolizumab (MK-3475) updates:

Deleted Risk Attribution:
- Occasional: Infection

Decreased in Risk Attribution:
- Changed to Also Reported on Pembrolizumab (MK-3475) Trials But With Insufficient Evidence for Attribution from Occasional (i.e., removed from the Risk Profile): Fluid in the joints; Pain in chest
- Provided Further Clarification: Rare: Changed from "Swelling and redness of the eye" to "Swelling and redness of the eye **which may cause blurred vision with a chance of blindness**"

PLEASE NOTE: The potential risks listed in the CAEPR whose relationship to pembrolizumab is still undetermined are not required by CTEP to be described in the ICD; however, they may be communicated to patients according to local IRB requirements.

Patient Notification and use of Consent Addendum:

Please note that the information provided below regarding patient notification and amendments to local consent forms reflects SWOG's minimum requirements. Sites should refer to the policies/procedures of the IRB of record to determine whether they have any more stringent requirements

SWOG has determined that the changes above that are **bolded** may affect a patient's willingness to participate in the study; therefore, SWOG requires that patients be notified of these changes.

Who must be informed?
- All patients currently on study treatment with pembrolizumab (MK-3475).

How must patients be notified?
- For patients currently receiving pembrolizumab (MK-3475): Notification must take place either via the attached Consent Addendum or via amended consent form by next study visit. After the change has been discussed with the patient, the patient must sign and date either the Consent Addendum or the 1/30/2023 version of the consent form.

What is the notification deadline and process?

- For patients currently receiving treatment with MK-3475 (pembrolizumab): Patients must be notified by their next scheduled visit or within 90 days after CTSU distribution of this revision, whichever is sooner.
- Sites using the NCI CIRB as their IRB of record: CIRB has approved the attached Consent Addendum; therefore, the Consent Addendum may be utilized immediately to notify patients of these changes.
- Sites not using the NCI CIRB as their IRB of record: If local IRB approval of the Consent Addendum is required before sites may utilize it, the site must still notify patients verbally prior to the notification deadline and notification must be documented in the patient chart. The site must then obtain patient signature on the Consent Addendum or updated consent form once the addendum and/or revised consent is locally approved. Important: Any changes to eligibility criterion are effective 30 days after distribution of this notice. If local IRB approval is not granted within 30 days, new registrations must meet any revised eligibility criteria included in the revision or accrual must be suspended until approval is obtained.

Regulatory Considerations:

Do local consent forms need to be updated?

- It depends. If your site will utilize the updated consent form for notification and formal reconsent then local consent forms must be updated. If your site will not utilize updated consent form for notification and formal reconsent then local consent forms need not be updated.

Can accrual continue until local implementation of the 1/30/2023 version of the consent form?

- Unless otherwise noted in the Action Letter, accrual may continue; however:
  - Patients enrolled after the notification deadline must be enrolled under the 1/30/2023 version of the consent form.
  - Sites using the NCI CIRB as their IRB of record: Patients enrolled prior to the notification deadline but before the 1/30/2023 version is implemented locally may be consented by signing the previous version of the consent form 3/21/2022 together with signing the attached Consent Addendum.
  - Sites not using the NCI CIRB as their IRB of record: Patients enrolled prior to the notification deadline but before the 1/30/2023 version is implemented locally may be consented by signing the previous version of the consent form 3/21/2022 together with being notified of the updated information verbally at the time of consent. The site must then obtain patient signature on the Consent Addendum or updated consent form once the addendum and/or revised consent is locally approved. Important: Any changes to eligibility criterion are effective 30 days after distribution of this notice. If local IRB approval is not granted within 30 days, new registrations must meet any revised eligibility criteria included in the revision or accrual must be suspended until approval is obtained.
  - **PLEASE NOTE: If the Action Letter requires suspension of accrual until the updated consent is implemented locally, the Action Letter instructions supersede this memo.**

The updated protocol and model informed consent form can be accessed from the CTSU website (www.ctsu.org). Please discard any previous versions of the documents and replace with the updated versions.

This study has been reviewed and approved by the NCI's Central Institutional Review Board (CIRB).

This memorandum serves to notify the NCI, and SWOG Statistics and Data Management Center.

cc: PROTOCOL & INFORMATION OFFICE

Informed Consent Addendum Model for S1512

S1512, "A Phase II and Pilot Trial of PD-1 Blockade with Pembrolizumab (MK-3475) in Patients with Resectable or Unresectable Desmoplastic Melanoma (DM)"

The following information should be read as an update to the original Consent form that you read and signed at the beginning of the study. Unless specifically stated below, all information contained in that original Consent Form is still true and remains in effect. Your participation continues to be voluntary. You may refuse to participate or may withdraw your consent to participate at any time, and for any reason, without jeopardizing your future care at this institution or your relationship with your study doctor.

New or additional information

The following risks have been identified with the Pembrolizumab (MK-3475) updates:

- **Rare: Changed from "Swelling and redness of the eye" to "Swelling and redness of the eye which may cause blurred vision with a chance of blindness"**

Patient Signature and Date

By signing this form, I acknowledge that I have read the information above or had it read to me. I have discussed it with a member of the study team and my questions have been answered. I understand that I will be given a copy of this form.

Participant's signature (or legally authorized representative) ______________________________

Date of signature_________________________

Signature of person(s) conducting the informed consent discussion________________________

Date of signature _________________________

CLOSED EFFECTIVE 06/04/2021

SWOG Authorized Document

PRIVILEGED COMMUNICATION
FOR INVESTIGATIONAL USE ONLY

Activation date October 20, 2016

SWOG

A PHASE II AND PILOT TRIAL OF PD-1 BLOCKADE WITH PEMBROLIZUMAB (MK-3475) IN PATIENTS WITH RESECTABLE OR UNRESECTABLE DESMOPLASTIC MELANOMA (DM)

NCT#02775851

CLOSED EFFECTIVE 06/04/2021

STUDY CHAIRS:

Kari Kendra, M.D., Ph.D. (Medical Oncology)
Ohio State University
B415 Starling Loving Hall, 320 W. 10th Ave.
Columbus, OH 43210
Phone: 614/293-4320
FAX: 614/293-2584
E-mail: kari.kendra@osumc.edu

Siwen Hu-Lieskovan, M.D., Ph.D. (Translational Med)
University of Utah Medical Center
Huntsman Cancer Institute
2000 Circle of Hope, Suite 2100
Salt Lake City, UT 84112
Phone: 801/585-1659
E-mail: siwen.hu-lieskovan@hci.utah.edu

William E. Carson III, M.D., FACS (Surgery)
The Ohio State University
410 W 10th Ave, N924 Doan Hall
Columbus, OH 43210-1228
Phone: 614/293-6306
Fax: 614/293-3465
Email: william.carson@osumc.edu

AGENTS:

NCI Supplied Investigational Agents

Pembrolizumab (MK-3475) (NSC-776864) (DCTD, NCI IND-125133)

STUDY CHAIRS (contd.):

David Wada, M.D. (Pathology)
University of Utah, Dept. of Dermatology
30N 1900E 4A330SOM
Salt Lake City, UT 84132
Phone: 801-585-0221/ 801-213-2098
E-mail: David.Wada@hsc.utah.edu

Jose A. Plaza, M.D. (Pathology)
The Ohio State University
930 Martha Morehouse Tower, 2050 Kenny Rd
Columbus, OH 43221
Phone: 614/293-9887
Fax: 614/293-7634
Email: JoseAPlaza@osumc.edu

BIOSTATISTICIANS:

Michael Wu, Ph.D.
James Moon, M.S.
SWOG Statistical Center
1100 Fairview Ave N, M3-C102
P.O. Box 190204
Seattle, WA 98109-1024
Phone: 206/667-4623
FAX: 206/667-4408
E-mail: mcwu@fredhutch.org
E-mail: jmoon@fredhutch.org

SWOG Authorized Document

PARTICIPANTS

ALL NATIONAL CLINICAL TRIALS NETWORK (NCTN) MEMBERS
SWOG/SWOG
ALLIANCE/Alliance for Clinical Trials in Oncology
ECOG-ACRIN/ECOG-ACRIN Cancer Research Group
NRG/NRG Oncology

CLOSED EFFECTIVE 06/04/2021

SWOG Authorized Document

TABLE OF CONTENTS

TITLE 1
PARTICIPANTS 2
TABLE OF CONTENTS 3
CANCER TRIALS SUPPORT UNIT (CTSU) ADDRESS AND CONTACT INFORMATION 5
SCHEMA 6
1.0 OBJECTIVES 7
1.1 Primary Objective 7
1.2 Secondary Objectives 7
1.3 Other objectives (all patients) 7
2.0 BACKGROUND 8
3.0 DRUG INFORMATION 10
3.1 Pembrolizumab (MK-3475) (NSC-776864, IND # 125133) 10
4.0 STAGING CRITERIA 20
5.0 ELIGIBILITY CRITERIA 21
5.1 Disease Related Criteria 21
5.2 Prior/Concurrent Therapy Criteria 22
5.3 Clinical/Laboratory Criteria 22
5.4 Specimen Criteria 23
5.5 Regulatory Criteria 23
6.0 STRATIFICATION FACTORS 23
7.0 TREATMENT PLAN 24
7.1 Supportive Care Guidelines 24
7.2 Treatment 24
7.3 **Surgical guidelines** 27
7.4 Prohibited and Cautionary Medications 27
7.5 Criteria for Removal from Protocol Treatment – Cohort A 28
7.6 Criteria for Removal from Protocol Treatment – Cohort B 28
7.7 Full CDUS Reporting Requirement 28
7.8 Discontinuation of Treatment 28
7.9 Follow-Up Period 28
8.0 TOXICITIES TO BE MONITORED AND DOSE MODIFICATIONS 29
8.1 NCI Common Terminology Criteria for Adverse Events 29
8.2 General Considerations 29
8.3 Pembrolizumab Dose Modification Guidelines for Drug-Related Adverse Events 29
8.4 Use of Transfusion and/or EPO 43
8.5 Use of G-CSF 43
8.6 Anti-Infectives 43
8.7 Dose Modification Contacts 43
8.8 Adverse Event Reporting 43
9.0 STUDY CALENDAR 44
9.1 Pembrolizumab (MK-3475)-Cohort A 44
9.2 Pembrolizumab (MK-3475)-Cohort B 48
10.0 CRITERIA FOR EVALUATION AND ENDPOINT ANALYSIS 51
10.1 Measurability of Lesions 51
10.2 Objective Status at Each Disease Evaluation 52
10.3 Best Response 53
10.4 Performance Status 54
10.5 Progression-Free Survival 54
10.6 Time to Death 55
10.7 Pathologic Complete Response 55
11.0 STATISTICAL CONSIDERATIONS 55
11.1 Cohort A 55
11.2 Cohort B 57

CLOSED EFFECTIVE 06/04/2021

SWOG Authorized Document

11.3 Data and Safety Monitoring 59
12.0 DISCIPLINE REVIEW 59
12.1 Pathology Review 59
13.0 REGISTRATION GUIDELINES 59
13.1 Registration Timing 59
13.2 Investigator/Site Registration 60
13.3 OPEN Registration Requirements 62
13.4 Registration Procedures 64
13.5 Exceptions to SWOG registration policies will not be permitted. 64
14.0 DATA SUBMISSION SCHEDULE 64
14.1 Data Submission Requirement 64
14.2 Master Forms 64
14.3 Data Submission Procedures 64
14.4 Data Submission Overview and Timepoints 65
15.0 SPECIAL INSTRUCTIONS 68
15.1 Specimen Submission for Translational Medicine 68
15.2 Specimen Submission for Central Confirmatory Quality Review 72
16.0 ETHICAL AND REGULATORY CONSIDERATIONS 74
16.1 Adverse Event Reporting Requirements 76
17.0 BIBLIOGRAPHY 82
18.0 APPENDIX 83
18.1 Translational Medicine Details 84
18.2 Specimen Banking Instructions for the UCLA Lab #224: The Ribas Laboratory 87
18.3 Specimen Banking Instructions for the SWOG Biospecimen Bank 88

CLOSED EFFECTIVE
06/04/2021

SWOG Authorized Document

CANCER TRIALS SUPPORT UNIT (CTSU) ADDRESS AND CONTACT INFORMATION

CONTACT INFORMATION

**For regulatory requirements:**	**For patient enrollments:**	**For study data submission:**
Regulatory documentation must be submitted to the CTSU via the Regulatory Submission Portal:<br><br>(Sign in at www.ctsu.org, and select the Regulatory Submission sub-tab under the Regulatory tab.)<br><br>Institutions with patients waiting that are unable to use the Portal should alert the CTSU Regulatory Office immediately at 866-651-2878 to receive further information and support.<br><br>Contact the CTSU Regulatory Help Desk at 866-651-2878 for regulatory assistance.	Please refer to the patient enrollment section of the protocol for instructions on using the Oncology Patient Enrollment Network (OPEN) which can be accessed at https://www.ctsu.org/OPEN_SYSTEM / or https://OPEN.ctsu.org.<br><br>Contact the CTSU Help Desk with any OPEN-related questions at ctsucontact@westat.com.	Data collection for this study will be done exclusively through Medidata Rave. Please see the data submission section of the protocol for further instructions.<br><br>Other Tools and Reports: Institutions participating through the CTSU continue to have access to other tools and reports available on the SWOG Workbench. Access this by using your active CTEP-IAM userid and password at the following url:<br><br>https://crawb.crab.org/TXWB/ctsulogon.aspx

The most current version of the **study protocol and all supporting documents** must be downloaded from the protocol-specific Web page of the CTSU Member Web site located at https://www.ctsu.org. Access to the CTSU members' website is managed through the Cancer Therapy and Evaluation Program - Identity and Access Management (CTEP-IAM) registration system and requires user log on with CTEP-IAM username and password. Permission to view and download this protocol and its supporting documents is restricted and is based on person and site roster assignment housed in the CTSU RSS.

For patient eligibility or data submission questions contact the SWOG Data Operations Center by phone or email:
206/652-2267
melanomaquestion@crab.org

For treatment or toxicity related questions contact the Study Chair by phone or email:
Kari Kendra, M.D., Ph.D.
Phone: 614-293-4320
E-mail: kari.kendra@osumc.edu

For non-clinical questions (i.e. unrelated to patient eligibility, treatment, or clinical data submission) contact the CTSU Help Desk by phone or e-mail:
CTSU General Information Line – 1-888-823-5923, or ctsucontact@westat.com. All calls and correspondence will be triaged to the appropriate CTSU representative.

The CTSU Website is located at https://www.ctsu.org.

CLOSED EFFECTIVE 06/04/2021

SWOG Authorized Document

SCHEMA

COHORT A

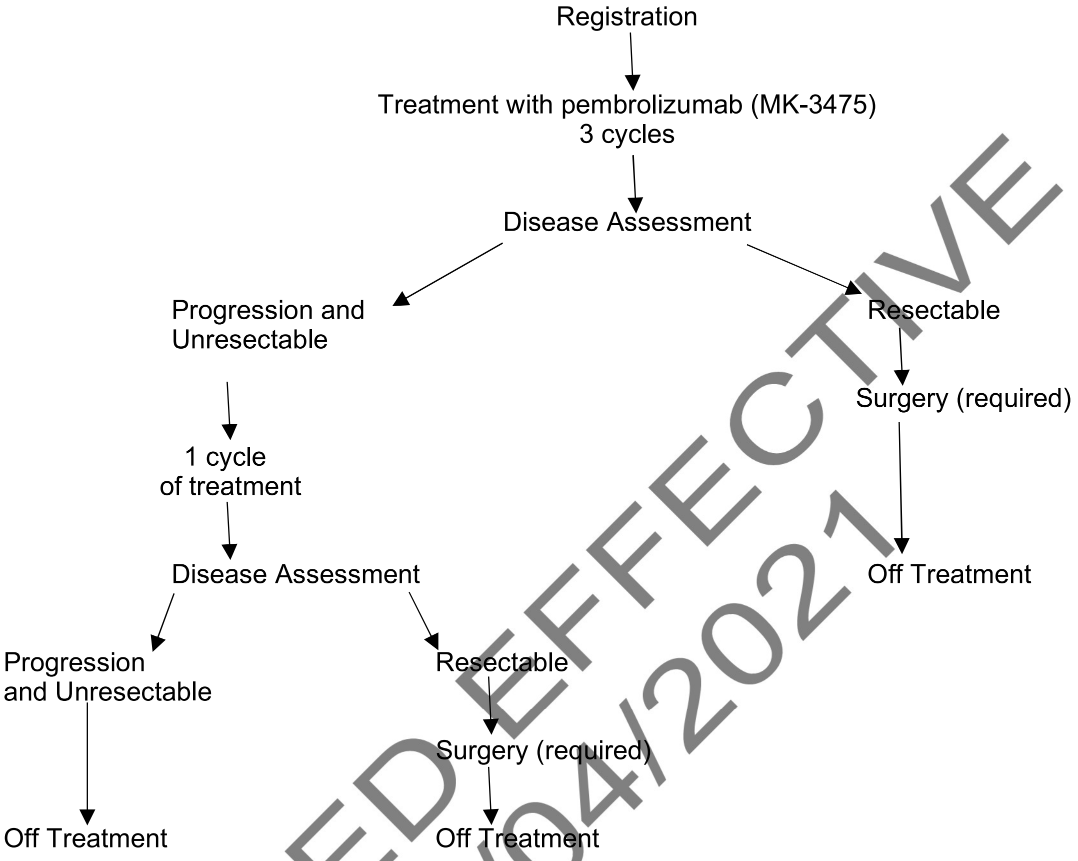

COHORT B

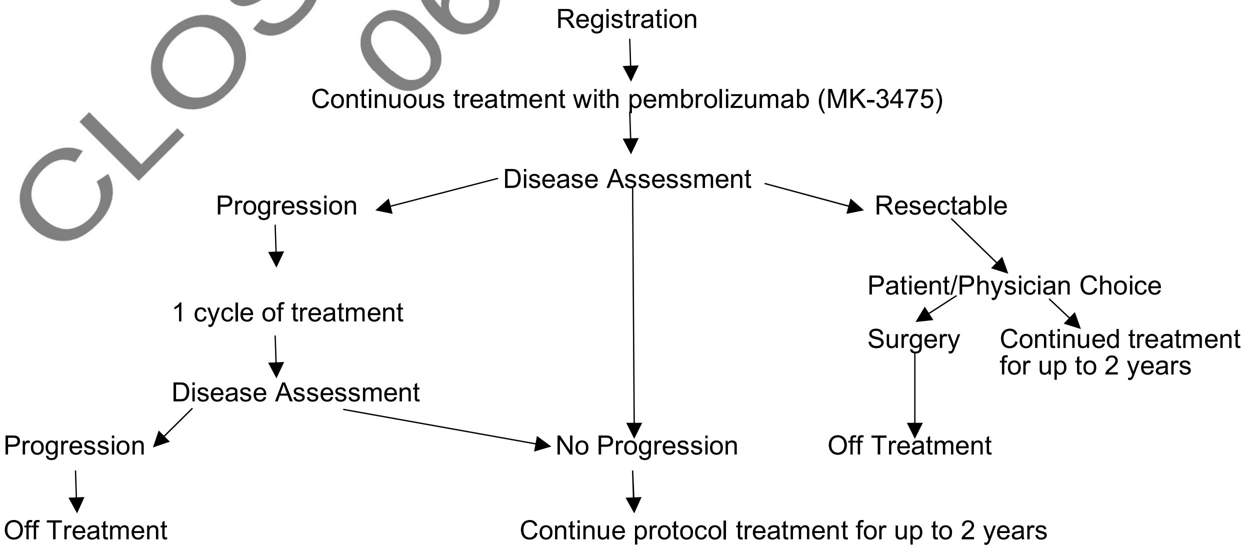

CLOSED EFFECTIVE
06/04/2021

SWOG Authorized Document

1.0 OBJECTIVES

This study will enroll two separate cohorts to assess the efficacy of pembrolizumab (MK-3475) in desmoplastic melanoma (DM). **Cohort A** will evaluate pembrolizumab (MK-3475) as neoadjuvant therapy for patients with DM that is deemed resectable by the treating investigator; including primary DM, locally advanced DM, and locally recurrent DM. **Cohort B** will be a pilot study to evaluate the use of pembrolizumab (MK-3475) for patients with DM that is deemed unresectable by the treating investigator, including metastatic DM.

1.1 Primary Objective

a. **Cohort A** - To evaluate the pathologic complete response rate (pCR as defined in Section 10.0) in patients with resectable desmoplastic melanoma treated with neoadjuvant pembrolizumab (MK-3475)

b. **Cohort B** - To evaluate the complete response rate (confirmed and unconfirmed in patients with unresectable desmoplastic melanoma treated with pembrolizumab (MK-3475).

1.2 Secondary Objectives

Cohort A

a. To estimate the 9 week response rate (RR) (unconfirmed complete and partial responses) among patients with measurable disease.

b. To estimate the median overall survival (OS).

c. To evaluate safety and tolerability of pembrolizumab (MK-3475) in the neoadjuvant setting.

Cohort B

a. To estimate the median progression-free survival (PFS).

b. To estimate the median overall survival (OS).

c. To evaluate safety and tolerability of pembrolizumab (MK-3475) in this setting.

1.3 Other objectives (all patients)

a. To evaluate the hypothesis that higher mutational load in the patient derived baseline tumor biopsy samples is associated with higher pathologic complete response (pCR).

b. To evaluate T cell infiltration into the tumors and circulating tumor DNA profile from blood samples in DM patients and correlate with response to PD-1 blockade.

c. To evaluate the clonality of tumor infiltrating T cells in DM patients and correlate with response to PD-1 blockade.

d. To evaluate adaptive immune resistant mechanism in DM tumors.

CLOSED EFFECTIVE 06/04/2021

SWOG Authorized Document

2.0 BACKGROUND

Rationale for selected approach and trial design.

Emerging evidence is being generated that a high mutational load may be one of the requisites for a melanoma to be immunogenic and detected by T cells, which are then turned off by PD-1/PD-L1 engagements. (*1,2*) Desmoplastic melanoma is one of the subtypes of melanoma with a known relationship to chronic skin sun damage and high mutational load. Anecdotal experience with pembrolizumab (MK-3475) suggests that desmoplastic melanoma may be uniquely sensitive to PD-1 blockade.

The programmed cell death 1 (PD-1) pathway represents a major immune checkpoint, which may be hijacked by tumor cells to overcome active T-cell immune surveillance. *(3,4,5)* The ligands for PD-1 (PD-L1 and PD-L2) can be constitutively expressed or can be induced in various tumors. *(6)* Preclinical in vitro and in vivo experiments have shown that PD-1 and/or PD-L1 blockade using monoclonal antibodies (mAb) enhances tumor-specific T-cell activation, T cell infiltration into the tumors, cytokine production, anti-tumor effector function, and clearance of tumor cells by the immune system. Pembrolizumab (MK-3475) strongly enhances T lymphocyte immune responses in cultured blood cells from healthy human donors, cancer patients, and primates. Pembrolizumab (MK-3475) also modulates the level of interleukin-2 (IL-2), tumor necrosis factor alpha (TNFα), interferon gamma (IFNγ), and other cytokines in the tumor microenvironment. The antibody potentiates existing immune responses only in the presence of antigen and does not nonspecifically activate T-cells.

Pembrolizumab (MK-3475) has recently been FDA approved under accelerated approval for the treatment of patients with unresectable or metastatic melanoma and disease progression following ipilimumab, and, if BRAF V600 mutation positive, treatment with a BRAF inhibitor. The indication is based on an overall response rate (ORR) of 24% and durability of response with 18 of the 21 responders demonstrating ongoing response with durations ranging from 1.4+ - 8.5+ months, including 8 patients with ongoing responses of 6 months or longer.

After analyzing biopsies from 46 patients with metastatic melanoma obtained before and during anti-PD1 therapy with pembrolizumab (MK-3475) using quantitative immunohistochemistry, quantitative multiplex immunofluorescence, and next generation sequencing for T-cell receptors (TCR), it became evident that responding patients showed proliferation of intratumoral $CD8^+$ T-cells that directly correlated with radiographic reduction in tumor size. *(7)* Pre-treatment samples obtained from responding patients showed higher numbers of CD8, PD1, and PD-L1 expressing cells at the invasive tumor margin and inside tumors, with close proximity between PD-1 and PD-L1, and a more clonal TCR repertoire. Using multivariate analysis, the authors established a predictive model based on CD8 expression at the invasive margin and validated the model in an independent cohort of 15 patients. These findings indicate that tumor regression following therapeutic PD-1 blockade requires pre-existing CD8+ T cells that are negatively regulated by PD-1/PD-L1 mediated adaptive immune resistance.

It is currently unknown which antigens are being recognized by T cells attacking tumors after release of PD-1 blockade. But evidence from studies with other immunotherapies suggests that most of these antigens are neoepitopes resultant from somatic mutations. In a recent experience, whole exome sequencing was performed on tumors and matching blood of patients treated with CTLA4 blockade with ipilimumab or tremelimumab. *(8)* Somatic mutations and candidate neoantigens generated from these mutations were characterized. Neoantigen peptides were tested for the ability to activate lymphocytes from ipilimumab-treated patients. A discovery set (n=25) consisted of 11 patients who derived long-term clinical benefit and 14 who derived minimal or no benefit. Mutational load was associated with clinical benefit (p=0.01), but alone was not

CLOSED EFFECTIVE 06/04/2021

SWOG Authorized Document

sufficient to predict benefit. Using genome-wide somatic neoepitope analysis and patient-specific HLA typing, the authors identified candidate tumor neoantigens for each patient. This work elucidated a neoantigen landscape that is specifically present in tumors with strong benefit from CTLA-4 blockade. This signature was validated in a second set of ipilimumab–treated melanoma patients (n=39). Predicted neoantigens activated T cells from the patients treated with ipilimumab.

Desmoplastic melanoma (DM) is a difficult tumor to remove surgically. *(9)* Frequently there is extensive perineural spread and surgery ends up being quite extensive with poor local control. Many times, these lesions present as small primaries only to find intraoperatively that they extend well beyond what is seen clinically or in imaging. *(10,11)* Patients are left with significant surgical deficits in the face of locally progressive disease. Due to the difficulty in gaining local control surgically, this study will offer patients with measurable desmoplastic melanoma treatment with pembrolizumab (MK-3475).

Patients with desmoplastic melanoma, locally advanced or metastatic, will be eligible to receive therapy on the trial. This will enhance the accrual rate. The primary end point of ORR after 3 cycles will normalize the data between the two groups while allowing for subsequent clinical intervention (ie surgery in those with locally advanced disease, or continued treatment in those with metastatic disease). The secondary endpoints of PFS and OS will also be collected for both groups. For individuals undergoing resection, this study will obtain data on the pCR and the ability to obtain clean margins. This study will evaluate tissue samples for mutational load and determine if the higher mutational load is associated with the ORR, in search of potential markers predictive of response. Tissue will also be evaluated for T cell infiltration, the clonality of tumor infiltrating T cells, and adaptive immune resistant mechanisms.

This trial hypothesizes that immune responses after administering pembrolizumab (MK-3475) will be also focused on neoepitopes resultant from somatic mutations, and that melanoma subsets with the highest mutational loads should have higher response rates than unselected melanomas. Among all melanomas, desmoplastic melanoma is a subset with a strong link to chronic UV exposure and high mutational load.

There are no prospective studies evaluating the response of desmoplastic melanoma to biologic therapies. This study has the potential to change the course of therapy for those with locally advanced disease, to improve overall survival in individuals with desmoplastic melanoma, and to obtain data that, if favorable, can be used further to determine predictive value of PD1 blockade with pembrolizumab (MK-3475).

Inclusion of Women and Minorities

This study was designed to include women and minorities, but was not designed to measure differences of intervention effects. The anticipated accrual in the ethnicity/race and sex categories is shown in the table below.

This study will be open to U.S. sites only.

CLOSED EFFECTIVE 06/04/2021

SWOG Authorized Document

DOMESTIC PLANNED ENROLLMENT REPORT					
Racial Categories	Ethnic Categories				Total
	Not Hispanic or Latino		Hispanic or Latino		
	Female	Male	Female	Male	
American Indian/Alaska Native	0	1	0	0	1
Asian	0	0	0	0	0
Native Hawaiian or Other Pacific Islander	0	0	0	0	0
Black or African American	0	1	0	0	1
White	16	37	0	1	54
More Than One Race	0	0	0	0	0
**Total**	16	39	0	1	56

3.0 DRUG INFORMATION

For information regarding Investigator's Brochures, please refer to SWOG Policy 15.

For this study, pembrolizumab (MK-3475) is investigational and is being provided under an IND held by the National Cancer Institute. The current version of the Investigator Brochure (IB) will be accessible to site investigators and research staff through the PMB Online Agent Order Processing (OAOP) application. Access to OAOP requires the establishment of a CTEP Identity and Access Management (IAM) account and the maintenance of an "active" account status and a "current" password. Questions about IB access may be directed via email to IBcoordinator@mail.nih.gov or by phone (240) 276-6575 Monday through Friday between 8:30 am and 4:30 pm (ET).

3.1 Pembrolizumab (MK-3475) (NSC-776864, IND # 125133)

a. PHARMACOLOGY

Pembrolizumab (MK-3475) is a humanized MAb of the IgG4/kappa isotype. The programmed cell death 1 (PD-1) receptor is an inhibitory receptor expressed by T cells. When bound to either of its ligands, PD-L1 or PD-L2, activated PD-1 negatively regulates T-cell activation and effector function. The pathway may be engaged by tumor cells expressing PD-1 ligands to suppress immune control. MK-3475 blocks the negative immune regulatory signaling by binding to the PD-1 receptor, inhibiting the interaction between PD-1 and its ligands and thereby promoting the host immune system to recognize tumor cells as foreign bodies to be eliminated.

CLOSED EFFECTIVE 06/04/2021

SWOG Authorized Document

b. PHARMACOKINETICS

The pharmacokinetic profile of pembrolizumab (MK-3475), with low clearance and limited volume of distribution, is typical for therapeutic antibodies. Elimination half-life after IV administration was approximately 14 to 21.6 days. Steady state concentration levels were achieved within 16 weeks of treatment when tested at 3 and 10 mg/kg dosing as administered at 2 week intervals. During repeated dosing of 2 or 10mg/kg Q3W, steady state in trough concentrations appeared to have been achieved after approximately three months. Furthermore, MK-3475 has a low potential of eliciting the formation of anti-drug antibodies.

c. ADVERSE EFFECTS

The Comprehensive Adverse Events and Potential Risks list (CAEPR) provides a single list of reported and/or potential adverse events (AE) associated with an agent using a uniform presentation of events by body system. In addition to the comprehensive list, a subset, the Specific Protocol Exceptions to Expedited Reporting (SPEER), appears in a separate column and is identified with bold and italicized text. This subset of AEs (SPEER) is a list of events that are protocol specific exceptions to expedited reporting to NCI (except as noted below). Refer to the 'CTEP, NCI Guidelines: Adverse Event Reporting Requirements' http://ctep.cancer.gov/protocolDevelopment/electronic_applications/docs/aeguidelines.pdf for further clarification. *Frequency is provided based on 3793 patients.* Below is the CAEPR for pembrolizumab (MK-3475).

NOTE: Report AEs on the SPEER **ONLY IF** they exceed the grade noted in parentheses next to the AE in the SPEER. If this CAEPR is part of a combination protocol using multiple investigational agents and has an AE listed on different SPEERs, use the lower of the grades to determine if expedited reporting is required.

Version 2.7, December 13, 2022[1]

**Adverse Events with Possible Relationship to Pembrolizumab (MK-3475) (CTCAE 5.0 Term) [n= 3793]**			**Specific Protocol Exceptions to Expedited Reporting (SPEER)**
**Likely (>20%)**	**Less Likely (<=20%)**	**Rare but Serious (<3%)**	
BLOOD AND LYMPHATIC SYSTEM DISORDERS			
	Anemia[2]		
		Blood and lymphatic system disorders - Other (immune thrombocytopenic purpura)[2]	
	Lymph node pain[2]		
CARDIAC DISORDERS			
		Myocarditis[2]	
		Pericarditis[2]	

CLOSED EFFECTIVE 04/2021

SWOG Authorized Document

Adverse Events with Possible Relationship to Pembrolizumab (MK-3475) (CTCAE 5.0 Term) [n= 3793]			Specific Protocol Exceptions to Expedited Reporting (SPEER)
Likely (>20%)	Less Likely (<=20%)	Rare but Serious (<3%)	
ENDOCRINE DISORDERS			
	Adrenal insufficiency[2]		
		Endocrine disorders - Other (hypoparathyroidism)	
	Endocrine disorders - Other (thyroiditis)[2]		
	Hyperthyroidism[2]		
	Hypophysitis[2]		
	Hypopituitarism[2]		
	Hypothyroidism[2]		
EYE DISORDERS			
		Uveitis[2]	
		Eye disorders - Other (Vogt-Koyanagi-Harada syndrome)	
GASTROINTESTINAL DISORDERS			
	Abdominal pain		
	Colitis[2]		
	Diarrhea[2]		***Diarrhea[2] (Gr 2)***
	Mucositis oral[2]		
	Nausea		***Nausea (Gr 2)***
	Pancreatitis[2]		
	Small intestinal mucositis[2]		
GENERAL DISORDERS AND ADMINISTRATION SITE CONDITIONS			
	Chills[2]		
Fatigue			***Fatigue (Gr 2)***
	Fever[2]		
HEPATOBILIARY DISORDERS			

CLOSED

SWOG Authorized Document

Adverse Events with Possible Relationship to Pembrolizumab (MK-3475) (CTCAE 5.0 Term) [n= 3793]			Specific Protocol Exceptions to Expedited Reporting (SPEER)
Likely (>20%)	Less Likely (<=20%)	Rare but Serious (<3%)	
	Hepatobiliary disorders - Other (autoimmune hepatitis)[2]		
		Hepatobiliary disorders - Other (sclerosing cholangitis)	
IMMUNE SYSTEM DISORDERS			
		Anaphylaxis[2]	
		Cytokine release syndrome[2]	
		Immune system disorders - Other (acute graft-versus-host-disease)[2,3]	
		Immune system disorders - Other (hemophagocytic lymphohistiocytosis)[2]	
	Immune system disorders - Other (sarcoidosis)[2]		
		Serum sickness[2]	
INJURY, POISONING AND PROCEDURAL COMPLICATIONS			
		Infusion related reaction	
INVESTIGATIONS			
	Alanine aminotransferase increased[2]		
	Alkaline phosphatase increased		
	Aspartate aminotransferase increased[2]		

CLOS

SWOG Authorized Document

Adverse Events with Possible Relationship to Pembrolizumab (MK-3475) (CTCAE 5.0 Term) [n= 3793]			Specific Protocol Exceptions to Expedited Reporting (SPEER)
**Likely (>20%)**	**Less Likely (<=20%)**	**Rare but Serious (<3%)**	
	Blood bilirubin increased		
		GGT increased	
		Serum amylase increased	
METABOLISM AND NUTRITION DISORDERS			
	Anorexia		
	Hyponatremia		
		Metabolism and nutrition disorders - Other (diabetic ketoacidosis)[2]	
		Metabolism and nutrition disorders - Other (type 1 diabetes mellitus)[2]	
MUSCULOSKELETAL AND CONNECTIVE TISSUE DISORDERS			
	Arthralgia[2]		***Arthralgia[2] (Gr 2)***
	Arthritis[2]		
	Back pain		
	Joint range of motion decreased		
	Myalgia[2]		
	Myositis[2]		
NERVOUS SYSTEM DISORDERS			
		Guillain-Barre syndrome[2]	
		Nervous system disorders - Other (myasthenic syndrome)[2]	
		Nervous system disorders - Other (neuromyopathy)[2]	
		Nervous system disorders - Other (non-infectious encephalitis)[2]	

CLOS

SWOG Authorized Document

Adverse Events with Possible Relationship to Pembrolizumab (MK-3475) (CTCAE 5.0 Term) [n= 3793]			Specific Protocol Exceptions to Expedited Reporting (SPEER)
**Likely (>20%)**	**Less Likely (<=20%)**	**Rare but Serious (<3%)**	
		Nervous system disorders - Other (non-infectious meningitis)[2]	
		Nervous system disorders - Other (non-infectious myelitis)	
		Nervous system disorders - Other (optic neuritis)	
		Nervous system disorders - Other (polyneuropathy)[2]	
		Paresthesia	
		Peripheral motor neuropathy[2]	
RENAL AND URINARY DISORDERS			
		Renal and urinary disorders - Other (autoimmune nephritis)[2]	
RESPIRATORY, THORACIC AND MEDIASTINAL DISORDERS			
	Cough		
	Pneumonitis[2]		
SKIN AND SUBCUTANEOUS TISSUE DISORDERS			
	Bullous dermatitis[2]		
		Erythema multiforme[2]	
	Erythroderma		
		Palmar-plantar erythrodysesthesia syndrome	
	Pruritus[2]		***Pruritus[2] (Gr 2)***
	Rash acneiform[2]		
	Rash maculo-papular[2]		***Rash maculo-papular[2] (Gr 2)***

CLOS

SWOG Authorized Document

Adverse Events with Possible Relationship to Pembrolizumab (MK-3475) (CTCAE 5.0 Term) [n= 3793]			Specific Protocol Exceptions to Expedited Reporting (SPEER)
**Likely (>20%)**	**Less Likely (<=20%)**	**Rare but Serious (<3%)**	
	Skin and subcutaneous tissue disorders - Other (dermatitis)[2]		
	Skin hypopigmentation[2]		
		Stevens-Johnson syndrome[2]	
		Toxic epidermal necrolysis	
	Urticaria[2]		
VASCULAR DISORDERS			
		Vasculitis[2]	

[1]This table will be updated as the toxicity profile of the agent is revised. Updates will be distributed to all Principal Investigators at the time of revision. The current version can be obtained by contacting PIO@CTEP.NCI.NIH.GOV. Your name, the name of the investigator, the protocol and the agent should be included in the e-mail.

[2]Immune-mediated adverse reactions have been reported in patients receiving Pembrolizumab (MK-3475). Adverse events potentially related to Pembrolizumab (MK-3475) may be manifestations of immune-mediated adverse events. In clinical trials, most immune-mediated adverse reactions were reversible and managed with interruptions of Pembrolizumab (MK-3475), administration of corticosteroids and supportive care.

[3]Acute graft-versus-host disease has been observed in patients treated with Pembrolizumab (MK-3475) who received hematopoeitic stem cell transplants.

CLOSED EFFECTIVE 06/04/20..

SWOG Authorized Document

Adverse events reported on Pembrolizumab (MK-3475) trials, but for which there is insufficient evidence to suggest that there was a reasonable possibility that Pembrolizumab (MK-3475) caused the adverse event:

BLOOD AND LYMPHATIC SYSTEM DISORDERS - Blood and lymphatic system disorders - Other (pancytopenia); Disseminated intravascular coagulation; Hemolysis
CARDIAC DISORDERS - Atrial fibrillation; Cardiac arrest; Chest pain - cardiac; Heart failure; Myocardial infarction; Pericardial effusion; Pericardial tamponade; Ventricular arrhythmia
EYE DISORDERS - Eye pain
GASTROINTESTINAL DISORDERS - Abdominal distension; Ascites; Constipation; Duodenal hemorrhage; Dysphagia; Gastritis; Gastrointestinal disorders - Other (diverticulitis); Gastrointestinal disorders - Other (intestinal obstruction); Gastrointestinal disorders - Other (intussusception); Oral pain; Rectal hemorrhage; Small intestinal perforation; Upper gastrointestinal hemorrhage; Vomiting
GENERAL DISORDERS AND ADMINISTRATION SITE CONDITIONS - Edema face; Edema limbs; Facial pain; Gait disturbance; General disorders and administration site conditions - Other (general physical health deterioration); Generalized edema; Malaise; Non-cardiac chest pain; Pain
INVESTIGATIONS - CPK increased; Cholesterol high; Creatinine increased; Fibrinogen decreased; Lymphocyte count decreased; Neutrophil count decreased; Platelet count decreased; Weight loss; White blood cell decreased
METABOLISM AND NUTRITION DISORDERS - Dehydration; Hypercalcemia; Hyperglycemia; Hyperkalemia; Hypertriglyceridemia; Hyperuricemia; Hypoalbuminemia; Hypokalemia; Hypophosphatemia; Metabolism and nutrition disorders - Other (failure to thrive); Tumor lysis syndrome
MUSCULOSKELETAL AND CONNECTIVE TISSUE DISORDERS - Bone pain; Generalized muscle weakness; Joint effusion[2]; Musculoskeletal and connective tissue disorder - Other (groin pain); Pain in extremity
NERVOUS SYSTEM DISORDERS - Aphonia; Depressed level of consciousness; Dysarthria; Edema cerebral; Encephalopathy; Headache; Hydrocephalus; Lethargy; Meningismus; Nervous system disorders - Other (brainstem herniation); Seizure; Syncope; Tremor
PSYCHIATRIC DISORDERS - Agitation; Confusion
RENAL AND URINARY DISORDERS - Acute kidney injury; Nephrotic syndrome; Proteinuria; Renal and urinary disorders - Other (hydronephrosis); Urinary incontinence; Urinary tract pain
REPRODUCTIVE SYSTEM AND BREAST DISORDERS - Pelvic pain
RESPIRATORY, THORACIC AND MEDIASTINAL DISORDERS - Dyspnea; Hypoxia; Laryngeal inflammation; Pleural effusion; Pleuritic pain[2]; Pneumothorax; Respiratory failure
SKIN AND SUBCUTANEOUS TISSUE DISORDERS - Alopecia; Dry skin; Skin and subcutaneous tissue disorders - Other (drug eruption)
VASCULAR DISORDERS - Hypertension; Peripheral ischemia; Thromboembolic event

Note: Pembrolizumab (MK-3475) in combination with other agents could cause an exacerbation of any adverse event currently known to be caused by the other agent, or the combination may result in events never previously associated with either agent.

CLOSED EFFECTIVE 05/04/2021

SWOG Authorized Document

1. Pregnancy and Lactation: The central function of the PD-1/PD-L1 pathway is to maintain immune tolerance to the fetal allograft, and its important role in maintaining pregnancy has been recently emphasized in literature [4-6, 4-7] The PD-L1 molecule expressed at the uteroplacental interface effectively protects the concepti from maternal T cell-mediated immunity. Blockade of PD-L1 signaling has been shown in murine models of allogeneic pregnancy [4-6, 4-7] to abrogate fetomaternal tolerance to the concepti and to result in an increase in fetal resorption. The evidence from these experimental results indicates that there is a theoretical risk associated with the administration of MK-3475 to women of child-bearing potential (WOCBP). It is therefore anticipated that the inhibition of PD-1 by treatment with anti-PD-1 monoclonal *antibody (i.e., MK-3475) during pregnancy would have detrimental effects that might include increased rates of abortion and stillbirth. Women/men of reproductive potential should use an effective contraceptive method while on treatment and through 120 days after the last dose of MK-3475.*

   It is not known whether MK-3475 is excreted in human milk. Because of the potential for drugs to be excreted in human milk, the risk to the nursing infant cannot be excluded and therefore MK-3475 should not be administered to nursing mothers.

   Drug Interactions: No studies on pharmacodynamic drug interactions have been performed. Due to potential drug interactions, a complete patient medication list, including MK-3475, should be screened prior to initiation of and during treatment with MK-3475. See Section 8.0 Toxicities to be Monitored and Dosage Modifications

d. DOSING & ADMINISTRATION

   See Section 7.0 Treatment Plan

e. HOW SUPPLIED

   Pembrolizumab (MK-3475) is supplied by Merck & Co., Inc. and distributed by the Pharmaceutical Management Branch, CTEP/DCTD/NCI. Pembrolizumab (MK-3475) injection is a sterile, preservative-free clear to slightly opalescent, colorless to slightly yellow solution for intravenous use. Each vial contains 100 mg of pembrolizumab (MK-3475) in 4 mL of solution. Each 1 mL of solution contains 25 mg of pembrolizumab (MK-3475) and is formulated in: L histidine (1.55 mg), polysorbate 80 (0.2 mg), sucrose (70 mg) and water for injection, USP.

f. STORAGE, PREPARATION & STABILITY

   1. **Preparation**: MK-3475 solution for infusion must be diluted prior to administration. Allow the required number of vials to equilibrate to room temperature. Do not shake the vials. Do not use if opaque or extraneous particulate matter other than translucent to white proteinaceous particles is observed. Do not use if discolored. To prepare the infusion solution add the dose volume of MK-3475 to an infusion bag containing 0.9% Sodium Chloride Injection, USP or 5% Dextrose Injection, USP. Gently invert the bag 10-15 times to mix the solution. The final concentration must be between 1 mg/mL to 10 mg/mL.

CLOSED EFFECTIVE 06/04/2021

SWOG Authorized Document

Compatible IV bag materials: PVC plasticized with DEHP, non-PVC (polyolefin), EVA, or PE lined polyolefin

2. **Storage**: Store intact vials between 2°C - 8°C (36°F - 46°F). Do not freeze. Protect from light by storing in the original box.

   If a storage temperature excursion is identified, promptly return MK-3475 to between 2-8°C and quarantine the supplies. Provide a detailed report of the excursion (including documentation of temperature monitoring and duration of the excursion) to PMBAfterHours@mail.nih.gov for determination of suitability.

3. **Stability**: Refer to the package label for expiration.

   Administer prepared solutions immediately after preparation. If not administered immediately, prepared solutions may be stored refrigerated for up to 24 hours. Pembrolizumab (MK-3475) solutions may be stored at room temperature for a cumulative time of up to 6 hours. This includes room temperature storage of liquid drug product solution in vials, room temperature storage of infusion solution in the IV bag, and the duration of infusion.

4. **Route of Administration**: IV infusion only. Do not administer as an IV push or bolus injection.

5. **Method of Administration**: Infuse over approximately 30 minutes (range: 25 - 40 minutes) using an infusion set containing a low-protein binding 0.2 to 5 µm in-line filter made of polyethersulfone or polysulfone. Infusion rate should not exceed 6.7 mL/min. A central line is not required; however if a patient has a central venous catheter in place, it is recommended that it be used for the infusion. Do not co-administer other drugs through the same infusion line. Following the infusion, flush the IV line with normal saline.

   Compatible infusion set materials: PVC plasticized with DEHP or DEHT, PVC and tri-(2-ethylhexyl) trimellitate, polyethylene lined PVC, polyurethane, or polybutadiene

g. DRUG ORDERING & ACCOUNTABILITY

1. Drug Ordering

   Starter supplies will not be provided. NCI supplied agents may be requested by the Principal Investigator (or their authorized designee) at each participating institution. Pharmaceutical Management Branch (PMB) policy requires that drug be shipped directly to the institution where the patient is to be treated. PMB does not permit the transfer of agents between institutions (unless prior approval from PMB is obtained). The CTEP assigned protocol number (**S1512**) must be used for ordering all CTEP supplied investigational agents. The responsible investigator at each participating institution must be registered with CTEP, DCTD through an annual submission of FDA Form 1572 and a CV. If there are several participating investigators at one institution, CTEP-supplied investigational agents for the study should be ordered under the name of one lead investigator at that institution. Active CTEP-registered investigators and

CLOSED EFFECTIVE 06/04/2021

SWOG Authorized Document

investigator-designated shipping designees and ordering designees can submit agent requests through the PMB Online Agent Order Processing (OAOP) application. Access to OAOP requires the -establishment of a CTEP Identity and Access Management (IAM) account and the maintenance of an "active" account status and a "current" password. For questions about drug orders, transfers, returns, or accountability, call or email PMB any time. Refer to the PMB's website for specific policies and guidelines related to agent management. In general, sites may order initial agent supplies when a patient is being screened for enrollment onto the study.

2. Drug Handling and Accountability (NCI logs or other)

The investigator, or a responsible party designated by the investigator, must maintain a careful record of the receipt, dispensing and final disposition of all agents received from the PMB using the appropriate NCI Investigational Agent (Drug) Accountability Record (DARF) available on the CTEP forms page. Store and maintain separate NCI Investigational Agent Accountability Records for each agent, strength, formulation and ordering investigator on this protocol.

3. Drug return and/or disposition instruction

Drug disposition: PMB will send a stock recovery letter when the agent is no longer suitable for use. All undispensed drug supplies should be returned to the PMB. When it is necessary to return study drug, investigators should return the study drug to the PMB using the NCI Return Agent Form available on the NCI home page (http://ctep.cancer.gov).

4. Drug Expiration: Stability testing is ongoing. PMB will send a stock recovery letter when notified that the agent is no longer suitable for use.

5. Contact Information

CTEP Forms, Templates, Documents: http://ctep.cancer.gov/forms/

NCI CTEP Investigator Registration: PMBRegPend@ctep.nci.nih.gov

PMB policies and guidelines: http://ctep.cancer.gov/branches/pmb/agent_management.htm

PMB Online Agent Order Processing (OAOP) application: https://eapps-ctep.nci.nih.gov/OAOP/pages/login.jspx

CTEP Identity and Access Management (IAM) account: https://eapps-ctep.nci.nih.gov/iam/

CTEP Associate Registration and IAM account help: ctepreghelp@ctep.nci.nih.gov

PMB email: PMBAfterHours@mail.nih.gov

PMB phone and hours of service: (240) 276-6575 Monday through Friday between 8:30 am and 4:30 pm (ET)

4.0 STAGING CRITERIA

CLOSED EFFECTIVE 06/04/2021

SWOG Authorized Document

Staging criteria are not required for eligibility. Please refer to Section 18.1 (Appendix) for staging definitions. Staging must be documented. Clinical staging may be used where appropriate.

5.0 ELIGIBILITY CRITERIA

Each of the criteria in the following section must be met in order for a patient to be considered eligible for registration. Use the spaces provided to confirm a patient's eligibility. For each criterion requiring test results and dates, please record this information on the Onstudy Form and submit via MediData Rave ® (see Section 14.0). Any potential eligibility issues should be addressed to the Data Operations Center in Seattle at 206/652-2267 or melanomaquestion@crab.org prior to registration.

In calculating days of tests and measurements, the day a test or measurement is done is considered Day 0. Therefore, if a test is done on a Monday, the Monday 1 week later would be considered Day 7. This allows for efficient patient scheduling without exceeding the guidelines. **If Day 7, 14, 28 or 42 falls on a weekend or holiday, the limit may be extended to the next working day.**

5.1 Disease Related Criteria

a. Cohort A: Patients must have histologically or cytologically confirmed primary desmoplastic melanoma (see Section 12.1 for definition) that is deemed resectable. The decision to perform surgery on patients must be based on good clinical judgment. Eligible patients for surgical resection must have disease that, in the judgment of the surgeon, is deemed completely resectable resulting in free surgical margins. Patients must have residual disease after initial biopsy which can be measurable or non-measurable disease per RECIST 1.1 (see Section 10.1). Residual disease can either be confirmed with FNA or if measurable disease is present, no FNA needs to be obtained.

OR

b. Cohort B: Patients must have histologically or cytologically confirmed primary desmoplastic melanoma (see Section 12.1 for definition) that is unresectable. Patients in Cohort B must have measurable disease per RECIST 1.1 (see Section 10.1).

c. Contrast-enhanced CT scans of the chest, abdomen and pelvis are required. A whole-body PET/CT scan with diagnostic quality images and intravenous iodinated contrast may be used in lieu of a contrast enhanced CT of the chest, abdomen and pelvis. Imaging of the head and neck is required only if the patient has a head/neck primary. Contrast may be omitted if the treating investigator believes that exposure to contrast poses an excessive risk to the patient. If skin lesions are being followed as measurable disease, photograph with a ruler included and physician measurements, must be kept in the patient's chart as source documentation. All measurable lesions must be assessed within 28 days prior to registration. Tests to assess non-measurable disease must be performed within 42 days prior to registration. All disease must be assessed and documented on the Baseline Tumor Assessment Form (RECIST 1.1).

CLOSED EFFECTIVE 06/04/2021

d. Patients must not have known brain metastases unless brain metastases have been treated and patient is asymptomatic with no residual neurological dysfunction and has not received enzyme-reducing anti-epileptic drugs or corticosteroids for at least 14 days prior to registration.

5.2 Prior/Concurrent Therapy Criteria

a. Patients must not have received prior systemic treatment for this melanoma.

b. Patients must not be planning to receive concomitant other biologic therapy, hormonal therapy, other chemotherapy, anti-cancer surgery or other anti-cancer therapy while on this protocol.

c. Patients must not have received radiation therapy, non-cytotoxic agents or investigational agents or systemic corticosteroids within 14 days prior to registration.

d. Patients may have received prior surgery. All adverse events associated with prior surgery must have resolved to ≤ Grade 1 (per CTCAE 4.0) prior to registration

5.3 Clinical/Laboratory Criteria

a. Patients must be ≥ 18 years of age.

b. Patients must have adequate bone marrow function as evidenced by all of the following: ANC ≥ 1,500/mcl; platelets ≥ 50,000/mcl; and hemoglobin ≥ 8 g/dL. These results must be obtained within 28 days prior to registration.

c. Patients must have adequate liver function as evidenced by the following: total bilirubin ≤ 1.5 x institutional upper limit of normal (IULN) (or ≤ 3.0 x IULN with Gilbert's Syndrome), and AST and ALT ≤ 2.5 x IULN (or < 5 x IULN for patients with known liver metastases). These results must be obtained within 28 days prior to registration.

d. Patients must have LDH performed within 28 days prior to registration.

e. Patients must have Zubrod Performance Status ≤ 2 (see Section 10.4).

f. Patients must not have history of (non-infectious) pneumonitis that required steroids or current pneumonitis.

g. Patients must not have an active infection requiring systemic therapy.

h. Patients must not have active autoimmune disease that has required systemic treatment in past 2 years (i.e., with use of disease modifying agents, corticosteroids or immunosuppressive drugs). Replacement therapy (e.g., thyroxine, insulin, or physiologic corticosteroid replacement therapy for adrenal or pituitary insufficiency, etc.) is not considered a form of systemic treatment.

i. Patients must not have received live vaccines within 42 days prior to registration. Examples of live vaccines include, but are not limited to, the following: measles, mumps, rubella, chicken pox, shingles, yellow fever, rabies, BCG, and typhoid (oral) vaccine. Seasonal influenza vaccines for injection are generally killed virus

CLOSED EFFECTIVE
06/04/2021

SWOG Authorized Document

vaccines and are allowed; however, intranasal influenza vaccines (e.g., Flu-Mist®) are live attenuated vaccines, and are not allowed.

j. Patients known to be HIV positive prior to registration are eligible if they meet the following criteria within 30 days prior to registration: stable and adequate CD4 counts (≥ 350 mm^3), and serum HIV viral load of < 25,000 IU/ml. Patients must be on a stable anti-viral therapy.

k. No other prior malignancy is allowed except for the following: adequately treated basal cell or squamous cell skin cancer, adequately treated in situ cancer, adequately treated Stage I or II cancer (including multiple primary melanomas) from which the patient is currently in complete remission, or any other cancer from which the patient has been disease free for three years.

l. Women of childbearing potential must have a negative urine or serum pregnancy test within 28 days prior to registration. Women/men of reproductive potential must have agreed to use an effective contraceptive method for the course of the study through 120 days after the last dose of study medication. Should a woman become pregnant or suspect she is pregnant while she or her partner is participating in this study, she should inform her treating physician immediately. A woman is considered to be of "reproductive potential" if she has had menses at any time in the preceding 12 consecutive months. In addition to routine contraceptive methods, "effective contraception" also includes heterosexual celibacy and surgery intended to prevent pregnancy (or with a side-effect of pregnancy prevention) defined as a hysterectomy, bilateral oophorectomy, or bilateral tubal ligation. However, if at any point a previously celibate patient chooses to become heterosexually active during the time period for use of contraceptive measures outlined in the protocol, he/she is responsible for beginning contraceptive measures. Patients must not be pregnant or nursing due to unknown teratogenic side effects.

5.4 Specimen Criteria

a. Patients must have specimens available and institutions must be planning to submit for centralized pathology review as outlined in Section 12.0 and for integrated translational medicine objectives as outlined in Section 15.1.

5.5 Regulatory Criteria

a. Patients must be informed of the investigational nature of this study and must sign and give written informed consent in accordance with institutional and federal guidelines.

b. As a part of the OPEN registration process (see Section 13.4 for OPEN access instructions) the treating institution's identity is provided in order to ensure that the current (within 365 days) date of institutional review board approval for this study has been entered in the system.

6.0 STRATIFICATION FACTORS

Patients will be stratified by the following factors: Cohort A (resectable) vs Cohort B (unresectable)

CLOSED EFFECTIVE
06/04/2021

SWOG Authorized Document

7.0 TREATMENT PLAN

For treatment or dose modification questions, please contact Dr. Kendra at 614/293-4320 or kari.kendra@osumc.edu or Dr. Hu-Lieskovan at 310/794-4955 or siwen.hu-lieskovan@hci.utah.edu. For dosing principles or questions, please consult the SWOG Policy #38 "Dosing Principles for Patients on Clinical Trials" at http://swog.org ("Policies and Manuals" under the "Visitors" menu and choose Policy 38).

7.1 Supportive Care Guidelines

Patients should receive appropriate supportive care measures as deemed necessary by the treating investigator. Suggested supportive care measures for the management of AEs with potential immunologic etiology are also outlined in the table in Section 8.3a. Where appropriate, these guidelines include the use of oral or IV treatment with corticosteroids as well as additional anti-inflammatory agents if symptoms do not improve with administration of corticosteroids. Note that several courses of steroid tapering may be necessary as symptoms may worsen when the steroid dose is decreased. For each disorder, attempts should be made to rule out other causes such as metastatic disease or bacterial or viral infection, which might require additional supportive care. The treatment guidelines are intended to be applied when the investigator determines the events to be related to pembrolizumab.

NOTE: If after the evaluation the event is determined not to be related, the investigator does not need to follow the treatment guidance (as outlined below).

It may be necessary to perform conditional procedures such as bronchoscopy, endoscopy, or skin photography as part of the evaluation of the event.

7.2 Treatment

a. Pretreatment Labs:

Blood samples collected for pretreatment laboratory tests may be collected and analyzed no more than 3 days prior to dosing at each cycle. Albumin, glucose, electrolytes (Na, K, HC03 or CO2), and thyroid function tests (T3, TSH and T4) are required to obtain baseline data for future toxicity assessments.

b. Cohort A: **Pembrolizumab (MK-3475) for resectable disease**

Agent	Dose	Route	Schedule
MK-3475 (pembrolizumab)	200 mg	IV over 30 minutes	Day 1, Q 3 weeks for 3 cycles *

1 cycle = 21 days.
* One additional cycle may be given for patients whose disease is unresectable after 3 cycles. See below.

Pembrolizumab (MK-3475) treatment should be administered after all procedures and assessments have been completed. Pembrolizumab (MK-3475) treatment may be administered up to 3 days before or after the protocol-specified Q 3 weeks due to administrative reasons.

Pembrolizumab (MK-3475) treatment will be administered on an outpatient basis.

CLOSED EFFECTIVE 06/04/2021

SWOG Authorized Document

Pembrolizumab (MK-3475) will be administered as a 30 minute IV infusion. Infusion timing should be as close to 30 minutes as possible; however, a window of -5 minutes and +10 minutes is permitted (*i.e.*, infusion time is 25-40 minutes).

- Disease assessment, using the same technique as baseline, will be performed every 9 weeks until discontinuation of protocol treatment. Patients with treatment delays may have additional disease assessments.
- After discontinuation of treatment, if the tumor is potentially resectable, the patients will proceed with surgical resection (see surgical guidelines below in Section 7.2). Pathology will be evaluated for evidence of pCR. If potentially resectable, surgery is required. This includes patients who have a complete response per RECIST 1.1 with no macroscopic evidence of disease.
- If at any time after the first disease assessment the patient's disease is deemed to be unresectable, treatment will continue for 1 more cycle and a repeat disease assessment, using the same techniques as baseline, must be obtained 4 weeks (Day 28 +/- 7 days) later. If, after this second disease assessment the patient's disease is deemed to be resectable the patient must continue with resection as previously planned. Patients who do not undergo a second disease assessment following an initial documentation of progression will be considered failures. [Note: Most responses to pembrolizumab (MK-3475) will occur within 3 cycles, but a delayed response is also possible. If the patient has become unresectable after 3 cycles, one more cycle is given to capture those who might have a delayed response to therapy. If the delayed response is present, unresectable disease may become resectable].

c. Cohort B: **<u>Pembrolizumab (MK-3475) for unresectable disease</u>**

Patients in Cohort B will receive the following treatment for a maximum of 34 cycles or until the patient has met any of the criteria in Section 7.5, whichever is first.

Agent	Dose	Route	Schedule
MK-3475 (pembrolizumab)	200 mg	IV over 30 minutes	Day 1, Q 3 weeks

1 cycle = 21 days.

Pembrolizumab (MK-3475) treatment should be administered after all procedures and assessments have been completed and the patient is deemed eligible. Pembrolizumab (MK-3475) treatment may be administered up to 3 days before or after the protocol-specified Q 3 weeks due to administrative reasons.

Pembrolizumab (MK-3475) treatment will be administered on an outpatient basis.

Pembrolizumab (MK-3475) will be administered as a 30 minute IV infusion. Infusion timing should be as close to 30 minutes as possible; however, a window of -5 minutes and +10 minutes is permitted (*i.e.*, infusion time is 25-40 minutes).

- Disease assessments, using the same technique as baseline, must be performed every 3 cycles (9 weeks) for the first 6 months. Scans should then be obtained every 4 months. Tumor measurements are determined with

CLOSED EFFECTIVE 06/04/2021

SWOG Authorized Document

scans or if the only site of measurable disease is cutaneous, with photographically measurable disease.

- Patients whose disease becomes resectable in the opinion of the treating investigator, may have surgery which must be performed as described below in Section 7.2. Patient may choose to continue protocol therapy instead of surgical resection.
- Patients may remain on protocol treatment after progression if they continue to derive clinical benefit in the opinion of the treating investigator.

CLOSED EFFECTIVE 06/04/2021

SWOG Authorized Document

7.3 **Surgical guidelines**

Definitive Surgical Procedure
The surgical procedure necessary to resect the tumor with negative margins should be used. A marker can be placed prior to initiating treatment or in early treatment at the surgeon's discretion to identify the site of disease if a complete response is noted. The approach used will be at the discretion of the surgeon performing the procedure using best clinical judgment and institutional guidelines. Repeat surgeries are allowed. Intent is for all sites of disease to be completely resected. Pathology from all of the resection(s) will need to be submitted for histologic review. For potentially resectable tumors, surgery is required.

Sentinel lymph node evaluation
Sentinel lymph nodes will be obtained per NCCN guidelines. If sentinel lymph nodes are positive, further nodal dissection will be performed per institutional guidelines.

Histological tumor assessment of the surgical specimens. Surgical specimens will be assessed for presence of tumor and tumor margin status.

- Tumor free margins (negative margins, resections defined by pathologist as absence of ink on the tumor), R0. All other resections of viable tumor are described as tumor positive margins resections, and are defined as
    - R1 resection – complete resection with no grossly visible tumor left behind as defined by the surgeon, or
    - R2 resection – partial resection with grossly visible tumor left behind as defined by the surgeon.

See Section 10.7 for definition of pathologic complete response.

7.4 Prohibited and Cautionary Medications

Patients are prohibited from receiving the following therapies before discontinuing protocol treatment during this trial:

- Anti-cancer systemic chemotherapy or biological therapy.
- Immunotherapy not specified in this protocol.
- Any non-study anti-cancer agent (investigational or non-investigational).
- Investigational agents other than MK-3475.
- Radiation therapy
- Live vaccines: Examples of live vaccines include, but are not limited to, the following: measles, mumps, rubella, chicken pox, shingles, yellow fever, rabies, BCG, and typhoid (oral) vaccine. Seasonal influenza vaccines for injection are generally killed virus vaccines and **are allowed**; however, intranasal influenza vaccines (*e.g.* Flu-Mist®) are live attenuated vaccines, and are not allowed.
- Glucocorticoids for any purpose other than to modulate symptoms from an event of suspected immunologic etiology. The use of physiologic doses of corticosteroids (defined as 10 mg prednisone) are acceptable, however site investigators should consult with the Study Chair for any dose higher than 10 mg prednisone.

Patients who, in the assessment by the investigator, require the use of any of the aforementioned treatments for clinical management should be removed from protocol treatment. Patients may receive other medications that the investigator deems to be medically necessary.

CLOSED EFFECTIVE
06/04/2021

SWOG Authorized Document

7.5 Criteria for Removal from Protocol Treatment – Cohort A

a. Progression of disease (as defined in Section 10.0) that renders the patient unable to have disease resected.

b. Patient chooses to proceed with surgical resection rather than continue on pembrolizumab.

c. Unacceptable toxicity.

d. The patient may withdraw from the study at any time for any reason.

e. The investigator may discontinue treatment if they determine that the patient's continued treatment on the study is detrimental to their long-term health, or due to poor compliance with the study's required visits and treatments.

f. Positive pregnancy test.

g. Completion of protocol treatment (after resection).

7.6 Criteria for Removal from Protocol Treatment – Cohort B

a. Progression of disease (as defined in Section 10.0). However, patients may remain on protocol treatment after progression if they continue to derive clinical benefit in the opinion of the treating investigator.

b. Disease becomes resectable and patient chooses to have surgical resection.

c. Unacceptable toxicity.

d. The patient may withdraw from the study at any time for any reason.

e. The investigator may discontinue treatment if they determine that the patient's continued treatment on the study is detrimental to their long-term health, or due to poor compliance with the study's required visits and treatments.

f. Positive pregnancy test.

g. Completion of protocol treatment (either resection or 34 cycles of pembrolizumab).

7.7 Full CDUS Reporting Requirement

Because this study contains an investigational drug for which CTEP holds the IND, it falls under CTEP requirements for full reporting. This involves required submission of cycle-specific toxicity and dose information (see Section 14.4c, the **S1512** Treatment Form, and the **S1512** Adverse Event Form). A cycle is defined as 21 days.

7.8 Discontinuation of Treatment

All reasons for discontinuation of treatment must be documented in the Off Treatment Notice.

7.9 Follow-Up Period
All patients will be followed until death or 5 years after initial registration, whichever occurs first.

CLOSED EFFECTIVE 06/04/2021

SWOG Authorized Document

8.0 TOXICITIES TO BE MONITORED AND DOSE MODIFICATIONS

8.1 NCI Common Terminology Criteria for Adverse Events

This study will utilize the CTCAE (NCI Common Terminology Criteria for Adverse Events) Version 5.0 for toxicity and Serious Adverse Event reporting. A copy of the CTCAE Version 5.0 can be downloaded from the CTEP home page (http://ctep.cancer.gov). All appropriate treatment areas should have access to a copy of the CTCAE Version 5.0.

8.2 General Considerations

a. Missed doses of pembrolizumab should be made up.

b. Refer to Section 7.1 and 8.3a for supportive care information for pembrolizumab.

c. Dosing interruptions are permitted in the case of medical/surgical events or logistical reasons not related to study therapy (*e.g.*, elective surgery, unrelated medical events, patient vacation, and/or holidays). Patients should be placed back on study therapy within 3 weeks of the scheduled interruption, unless otherwise discussed with the study chair. The reason for interruption should be documented in the patient's study record.

8.3 Pembrolizumab Dose Modification Guidelines for Drug-Related Adverse Events

Pembrolizumab must be withheld for drug-related toxicities and severe or life-threatening AEs as described below.

Adverse events (both nonserious and serious) associated with pembrolizumab exposure may represent an immunologic etiology. These immune-related AEs (irAEs) may occur shortly after the first dose or several months after the last dose of pembrolizumab treatment and may affect more than one body system simultaneously. Therefore, early recognition and initiation of treatment is critical to reduce complications. Based on existing clinical study data, most irAEs were reversible and could be managed with interruptions of pembrolizumab, administration of corticosteroids and/or other supportive care. For suspected irAEs, ensure adequate evaluation to confirm etiology or exclude other causes. Additional procedures or tests such as bronchoscopy, endoscopy, skin biopsy may be included as part of the evaluation. Based on the severity of irAEs, withhold or permanently discontinue pembrolizumab and administer corticosteroids. See Section 8.3a, Table 1, below.

Pembrolizumab may cause severe or life-threatening infusion-reactions including severe hypersensitivity or anaphylaxis. Signs and symptoms usually develop during or shortly after drug infusion and generally resolve completely within 24 hours of completion of infusion. Dose modification and toxicity management guidelines for irAEs and infusion reactions associated with pembrolizumab are provided in Section 8.3b, Table 2, below.

NOTE that non-irAEs will be managed as appropriate, following clinical practice recommendations.

CLOSED EFFECTIVE 06/04/2021

SWOG Authorized Document

a. Dose Modification and Toxicity Management for Immune-related Adverse Events Associated with Pembrolizumab

Table 1 Dose Modification and Toxicity Management Guidelines for Immune-related AEs and Infusion Reactions Associated with Pembrolizumab

General instructions:

1. For non-endocrine-related severe and life-threatening irAEs, investigators should consider the use of IV corticosteroids followed by oral steroids. Other immunosuppressive treatment should begin if the irAEs are not controlled by corticosteroids. Some non-endocrine irAEs do not require steroids. For example, celiac disease induced by pembrolizumab can be controlled by diet alone.
2. For non-endocrine-related toxicities, pembrolizumab must be permanently discontinued if the irAE does not resolve or the corticosteroid dose is not ≤10 mg/day within 12 weeks of the last pembrolizumab treatment.
3. Generally, when corticosteroids are used, investigators should begin a taper when the irAE is ≤Grade 1 and continue at least 4 weeks.
4. If pembrolizumab has been withheld due to a non-endocrine irAE, pembrolizumab may generally resume after the irAE has decreased to ≤Grade 1 after a corticosteroid taper.

irAEs	Toxicity grade (CTCAE V5.0)	Action with pembrolizumab	Corticosteroid and/or other therapies	Monitoring and follow-up
Pneumonitis	Grade 2	Withhold	Administer corticosteroids (initial dose of 1 to 2 mg/kg prednisone or equivalent) followed by taper<br>Add prophylactic antibiotics for opportunistic infections	Monitor participants for signs and symptoms of pneumonitis<br>Evaluate participants with suspected pneumonitis with radiographic imaging and initiate corticosteroid treatment
	Recurrent Grade 2, Grade 3 or 4	Permanently discontinue		

CLOSED EFFECTIVE 06/04/2021

SWOG Authorized Document

Diarrhea / Colitis	Grade 2 or 3	Withhold	Administer corticosteroids (initial dose of 1 to 2 mg/kg prednisone or equivalent) followed by taper<br>Patients who do not respond to corticosteroids should be seen by a gastroenterologist for confirmation of the diagnosis and consideration of secondary immune suppression	Monitor participants for signs and symptoms of enterocolitis (*i.e.*, diarrhea, abdominal pain, blood or mucus in stool with or without fever) and of bowel perforation (*i.e.* peritoneal signs and ileus)<br>Specifically assess for celiac disease serologically, and exclude *Clostridium difficile* infection<br>Participants with ≥Grade 2 diarrhea suspecting enterocolitis should consider GI consultation and performing endoscopy to rule out enterocolitis and assess mucosal severity<br>Participants with diarrhea/colitis should be advised to drink liberal quantities of clear fluids. If sufficient oral fluid intake is not feasible, fluid and electrolytes should be substituted via IV infusion
	Recurrent Grade 3 or Grade 4	Permanently discontinue		
AST or ALT elevation or Increased Bilirubin	Grade 2 [a]	Withhold	Administer corticosteroids (initial dose of 0.5 to 1 mg/kg prednisone or equivalent) followed by taper	Monitor with liver function tests (consider weekly or more frequently until liver enzyme value returned to baseline or is stable)
	Grade 3 [b] or 4 [c]	Permanently discontinue	Administer corticosteroids (initial dose of 1 to 2 mg/kg prednisone or equivalent) followed by taper	

CLOSED EFFECTIVE 06/04/2021

SWOG Authorized Document

Type 1 diabetes mellitus (T1DM) or Hyperglycemia	Grade 1 or 2	Continue		Investigate for diabetes. In the absence of corticosteroids or diabetes medication non-adherence, any grade hyperglycemia may be an indication of beta-cell destruction and pembrolizumab-induced diabetes akin to type 1 diabetes. This should be treated as a Grade 3 event. Given this risk, exercise caution in utilizing non-insulin hypoglycemic agents in this setting. After a thorough investigation of other potential causes, which may involve a referral to an endocrinologist, follow institutional guidelines.
	New onset T1DM (evidence of β-cell failure) or Grade 3 or 4 hyperglycemia	Withhold [d] Resume pembrolizumab when symptoms resolve and glucose levels are stable	Initiate treatment with insulin<br>If patient is found to have diabetic ketoacidosis or hyperglycemic hyperosmolar syndrome, treat as per institutional guidelines with appropriate management and laboratory values (*e.g.* anion gap, ketones, blood pH, *etc.*) reported	Monitor for glucose control<br>Strongly consider referral to endocrinologist<br>Obtain C-peptide level paired with glucose, autoantibody levels (*e.g.* GAD65, islet cell autoantibodies), and hemoglobin A1C level

CLOSED EFFECTIVE 06/04/2021

Hypophysitis	Grade 2	Withhold	Administer corticosteroids and initiate hormonal replacements as clinically indicated	Monitor for signs and symptoms of hypophysitis (including hypopituitarism and adrenal insufficiency)<br>Provide adrenal insufficiency precautions including indications for stress dose steroids and medical alert jewelry<br>Strongly consider referral to endocrinologist
	Grade 3 or 4	Withhold or permanently discontinue [d]		
Hyperthyroidism	Grade 2	Consider withholding. Resume pembrolizumab when symptoms are controlled, and thyroid function is improving	Treat with nonselective beta-blockers (*e.g.*, propranolol) or thionamides as appropriate<br>Initiate treatment with anti-thyroid drug such as methimazole or carbimazole as needed	Monitor for signs and symptoms of thyroid disorders<br>Strongly consider referral to endocrinologist
	Grade 3 or 4	Withhold or permanently discontinue [d]		
Hypothyroidism	Grade 2, 3 or 4	Continue	Initiate thyroid replacement hormones (*e.g.*, levothyroxine or liothyronine) per standard of care	Monitor for signs and symptoms of thyroid disorders

CLOSED EFFECTIVE 06/04/2021

SWOG Authorized Document

Nephritis: grading according to increased creatinine or acute kidney injury	Grade 2	Withhold	Administer corticosteroids (prednisone 1 to 2 mg/kg or equivalent) followed by taper	Monitor changes of renal function<br>Strongly consider referral to nephrologist
	Grade 3 or 4	Permanently discontinue		
Cardiac Events (including myoca	Asymptomatic cardiac enzyme elevation with clinical suspicion of myocarditis (previously CTCAE v4.0 Grade 1), or Grade 1	Withhold	Based on severity of AE administer corticosteroids	Ensure adequate evaluation to confirm etiology and/or exclude other causes<br>Strongly consider referral to cardiologist and cardiac MRI<br>Consider endomyocardial biopsy<br>If event resolves to Grade 1 or better, taper corticosteroids over ≥1 month

CLOSED EFFECTIVE 06/04/2021

SWOG Authorized Document

rditis, pericarditis, arrhythmias, impaired ventricular function, vasculitis)	Grade 2, 3 or 4	Permanently discontinue	Initiate treatment with corticosteroids equivalent to 1-2 mg/kg/day IV methylprednisolone and convert to 1-2 mg/kg/day oral prednisone or equivalent upon improvement<br>If event does not improve within 48 hours after initiating corticosteroids, consider adding an immunosuppressive agent<br>Initiate treatment per institutional guidelines and consider antiarrhythmic drugs, temporary pacemaker, extracorporeal membrane oxygenation (ECMO), ventricular assist device (VAD), or pericardiocentesis as appropriate	Ensure adequate evaluation to confirm etiology and/or exclude other causes<br>Strongly consider referral to cardiologist and cardiac MRI<br>Consider endomyocardial biopsy<br>If event resolves to Grade 1 or better, taper corticosteroids over ≥1 month
Exfolia	Suspected SJS, TEN, or DRESS	Withhold		

CLOSED EFFECTIVE 06/04/2021

SWOG Authorized Document

tive Dermatologic Conditions	Confirmed SJS, TEN, or DRESS	Permanently discontinue	Based on severity of AE administer corticosteroids	Ensure adequate evaluation to confirm etiology or exclude other causes<br>Strongly consider referral to dermatologist<br>Consider skin biopsy for evaluation of etiology
All Other irAEs	Persistent Grade 2	Withhold	Based on severity of AE administer corticosteroids	Ensure adequate evaluation to confirm etiology or exclude other causes
	Grade 3	Withhold or discontinue based on the event[e]		
	Recurrent Grade 3 or Grade 4	Permanently discontinue		

AE(s)=adverse event(s); ALT= alanine aminotransferase; AST=aspartate aminotransferase; CTCAE=Common Terminology Criteria for Adverse Events; DRESS=Drug Rash with Eosinophilia and Systemic Symptom; ECMO=extracorporeal membrane oxygenation; GI=gastrointestinal; ICU=intensive care unit; IO=immuno-oncology; ir=immune related; IV=intravenous; MRI=magnetic resonance imaging; PO=per os; SJS=Stevens-Johnson Syndrome; T1DM=type 1 diabetes mellitus; TEN=Toxic Epidermal Necrolysis; ULN=upper limit of normal; VAD=ventricular assist device.

Note: Non-irAE will be managed as appropriate, following clinical practice recommendations.

[a] AST/ALT: >3.0 to 5.0 x ULN if baseline normal; >3.0 to 5.0 x baseline, if baseline abnormal; bilirubin:>1.5 to 3.0 x ULN if baseline normal; >1.5 to 3.0 x baseline if baseline abnormal

[b] AST/ALT: >5.0 to 20.0 x ULN, if baseline normal; >5.0 to 20.0 x baseline, if baseline abnormal; bilirubin:>3.0 to 10.0 x ULN if baseline normal; >3.0 to 10.0 x baseline if baseline abnormal

[c] AST/ALT: >20.0 x ULN, if baseline normal; >20.0 x baseline, if baseline abnormal; bilirubin: >10.0 x ULN if baseline normal; >10.0 x baseline if baseline abnormal

[d] The decision to withhold or permanently discontinue pembrolizumab is at the discretion of the investigator or treating physician. If control achieved or ≤Grade 2, pembrolizumab may be resumed.

[e] Events that require discontinuation include but are not limited to: encephalitis and other clinically important irAEs (*e.g.* vasculitis and sclerosing cholangitis).

CLOSED EFFECTIVE 06/04/2024

SWOG Authorized Document

Appropriate resuscitation equipment should be available at the bedside and a physician readily available during the period of drug administration. For further information, please refer to the Common Terminology Criteria for Adverse Events v5.0 (CTCAE) at http://ctep.cancer.gov.

b. Management of Infusion Reactions

- Signs and symptoms usually develop during or shortly after drug infusion and generally resolve completely within 24 hours of completion of infusion.

  Table 2 below shows treatment guidelines for patients who experience an infusion reaction associated with administration of pembrolizumab (MK-3475).

CLOSED EFFECTIVE
06/04/2021

SWOG Authorized Document

Table 2 Pembrolizumab (MK-3475) Infusion Reaction Treatment Guidelines

**Infusion Reactions**	**NCI CTCAE Grade**	**Treatment**	**Premedication at subsequent dosing**
Mild reaction; infusion interruption not indicated; intervention not indicated	Grade 1	Increase monitoring of vital signs as medically indicated until the participant is deemed medically stable in the opinion of the investigator.	None
Requires therapy or infusion interruption but responds promptly to symptomatic treatment (*e.g.*, antihistamines, NSAIDs, narcotics, IV fluids); prophylactic medications indicated for ≤24 hrs.	Grade 2	• **Stop Infusion.**<br>• Additional appropriate medical therapy may include but is not limited to:<br>• IV fluids<br>• Antihistamines<br>• NSAIDs<br>• Acetaminophen<br>• Narcotics<br>• Increase monitoring of vital signs as medically indicated until the participant is deemed medically stable in the opinion of the investigator.<br>• If symptoms resolve within 1 hour of stopping drug infusion, the infusion may be restarted at 50% of the original infusion rate (*e.g.* from 100 mL/hr. to 50 mL/hr.). Otherwise dosing will be held until symptoms resolve and the participant should be premedicated for the next scheduled dose.<br>**Participants who develop Grade 2 toxicity despite adequate premedication should be permanently discontinued from further protocol treatment**	Participant may be premedicated 1.5h (± 30 minutes) prior to infusion of pembrolizumab (MK-3475)with:<br>Diphenhydramine 50 mg PO (or equivalent dose of antihistamine).<br>Acetaminophen 500-1000 mg PO (or equivalent dose of analgesic).

CLOS[illegible] EFFECTIVE 04/2021

SWOG Authorized Document

Infusion Reactions	NCI CTCAE Grade	Treatment	Premedication at subsequent dosing
Prolonged (*i.e.*, not rapidly responsive to symptomatic medication and/or brief interruption of infusion); recurrence of symptoms following initial improvement; hospitalization indicated for other clinical sequelae (e.g., renal impairment, pulmonary infiltrates)	Grade 3	• **Stop Infusion.**<br>• Additional appropriate medical therapy may include but is not limited to:<br>• Epinephrine**<br>• IV fluids<br>• Antihistamines<br>• NSAIDs<br>• Acetaminophen<br>• Narcotics<br>• Oxygen<br>• Pressors<br>• Corticosteroids (*e.g.* methylprednisolone 2 mg/kg/day or dexamethasone 10 mg every 6 hours)<br>• Increase monitoring of vital signs as medically indicated until the participant is deemed medically stable in the opinion of the investigator.<br>• Hospitalization may be indicated.<br>**In cases of anaphylaxis, epinephrine should be used immediately.<br>**Participant is permanently discontinued from further study drug treatment.**	No subsequent dosing.

CLOSED EFFECTIVE 06/04/2021

SWOG Authorized Document

Infusion Reactions	NCI CTCAE Grade	Treatment	Premedication at subsequent dosing
Life-threatening; pressor or ventilator support indicated	Grade 4	Admit participant to intensive care unit (ICU) and initiate hemodynamic monitoring, mechanical ventilation, and/or IV fluids and vasopressors as needed. Monitor other organ function closely.<br>Manage constitutional symptoms and organ toxicities as per institutional practice.<br>Follow Grade 3 recommendations as applicable.	No subsequent dosing.
Appropriate resuscitation equipment should be available at the bedside and a physician readily available during the period of drug administration. For further information, please refer to the Common Terminology Criteria for Adverse Events v5.0 (CTCAE) at http://ctep.cancer.gov.			

CLOSED EFFECTIVE 06/04/2021

SWOG Authorized Document

c. Management of Neurological Toxicities

Table 3 Neurological Toxicities

Event	Management
Immune-mediated neuropathy, Grade 1	• Continue pembrolizumab.<br>• Investigate etiology.<br>• Any cranial nerve disorder (including facial paresis) should be managed as per Grade 2 management guidelines below.
Immune-mediated neuropathy, including facial paresis, Grade 2	• Withhold pembrolizumab for up to 12 weeks after event onset. [a]<br>• Investigate etiology and refer patient to neurologist.<br>• Initiate treatment as per institutional guidelines.<br>• For general immune-mediated neuropathy:<br>○ If event resolves to Grade 1 or better, resume pembrolizumab. [b]<br>○ If event does not resolve to Grade 1 or better while withholding pembrolizumab, permanently discontinue pembrolizumab. [c]<br>• For facial paresis:<br>○ If event resolves fully, resume pembrolizumab. [b]<br>○ If event does not resolve fully while withholding pembrolizumab, permanently discontinue pembrolizumab. [c]
Immune-mediated neuropathy, including facial paresis, Grade 3 or 4	• Permanently discontinue pembrolizumab. [c]<br>• Refer patient to neurologist.<br>• Initiate treatment as per institutional guidelines.
Myasthenia gravis and Guillain-Barré syndrome (any grade)	• Permanently discontinue pembrolizumab. [c]<br>• Refer patient to neurologist.<br>• Initiate treatment as per institutional guidelines.<br>• Consider initiation of corticosteroids equivalent to 1–2 mg/kg/day oral or IV prednisone.

[a] Pembrolizumab may be withheld for a longer period of time (*i.e.*, >12 weeks after event onset) to allow for corticosteroids (if initiated) to be reduced to the equivalent of ≤10 mg/day oral prednisone. The acceptable length of the extended period of time must be based on an assessment of benefit–risk by the investigator and in alignment with the protocol requirements for the duration of treatment and documented by the investigator.
[b] If corticosteroids have been initiated, they must be tapered over ≥1 month to the equivalent of ≤10 mg/day oral prednisone before pembrolizumab can be resumed.
[c] Resumption of pembrolizumab may be considered in patients who are deriving benefit and have fully recovered from the immune-mediated event. The decision to re-challenge patients with pembrolizumab should be based on investigator's assessment of benefit–risk and documented by the investigator (or an appropriate delegate).

CLOSED EFFECTIVE 06/04/2021

SWOG Authorized Document

Event	Management
Immune-mediated myelitis, Grade 1	• Continue pembrolizumab unless symptoms worsen or do not improve.<br>• Investigate etiology and refer patient to a neurologist.
Immune-mediated myelitis, Grade 2	• Permanently discontinue pembrolizumab.<br>• Investigate etiology and refer patient to a neurologist.<br>• Rule out infection.<br>• Initiate treatment with corticosteroids equivalent to 1-2 mg/kg/day oral prednisone.
Immune-mediated myelitis, Grade 3 or 4	• Permanently discontinue pembrolizumab.<br>• Refer patient to a neurologist.<br>• Initiate treatment as per institutional guidelines.

Event	Management
Immune-mediated meningoencephalitis, all grades	• Permanently discontinue pembrolizumab. [a]<br>• Refer patient to neurologist.<br>• Initiate treatment with corticosteroids equivalent to 1–2 mg/kg/day IV methylprednisolone and convert to 1–2 mg/kg/day oral prednisone or equivalent upon improvement.<br>• If event does not improve within 48 hours after initiating corticosteroids, consider adding an immunosuppressive agent.<br>• If event resolves to Grade 1 or better, taper corticosteroids over ≥1 month.

[a] Resumption of pembrolizumab may be considered in patients who are deriving benefit and have fully recovered from the immune-mediated event. The decision to re-challenge patients with pembrolizumab should be based on investigator's assessment of benefit–risk and documented by the investigator (or an appropriate delegate).

CLOSED EFFECTIVE 06/04/2021

SWOG Authorized Document

8.4 Use of Transfusion and/or EPO

Transfusions and/or erythropoietin may be utilized as clinically indicated for the treatment of anemia, but should be clearly noted as concurrent medications. Transfusion of platelets may be used if clinically indicated. ITP should be ruled out before initiation of platelet transfusion.

8.5 Use of G-CSF

Prophylactic use of colony-stimulating factors including Granulocyte Colony-Stimulating Factor (G-CSF), pegylated G-CSF or Granulocyte Macrophage Colony-Stimulating Factor GM CSF is not allowed in this study. Therapeutic use of G-CSF is allowed in patients with Grade 3-4 febrile neutropenia.

8.6 Anti-Infectives

Patients with a documented infectious complication should receive oral or IV antibiotics or other anti-infective agents as considered appropriate by the treating Investigator for a given infectious condition, according to standard institutional practice.

8.7 Dose Modification Contacts

For treatment or dose modification questions, please contact Dr. Kendra at 614/293-4320 or kari.kendra@osumc.edu or Dr. Hu-Lieskovan at 310/794-4955 or siwen.hu-lieskovan@hci.utah.edu. For dosing principles or questions, please consult the SWOG Policy #38 "Dosing Principles for Patients on Clinical Trials" at http://swog.org (then click on "Policies and Manuals" under the "Visitors" menu and choose Policy 38).

8.8 Adverse Event Reporting

Toxicities (including suspected reactions) that meet the expedited reporting criteria as outlined in Section 16.1 of the protocol must be reported to the Operations Office, Study Chair and NCI via CTEP-AERS, and to the IRB per local IRB requirements.

CLOSED EFFECTIVE 06/04/2021

SWOG Authorized Document

9.0 STUDY CALENDAR

9.1 Pembrolizumab (MK-3475)-Cohort A

REQUIRED STUDIES	Pre-study (screening) [t]	ON TREATMENT Each cycle consists of 1 infusion. Infusions to be given q 3 weeks. [aq]				End of Treatment assessment [u]	Surgical Resection [s]	Post treatment F/U prior to progression/ relapse[c]	Post treatment F/U post-progression/ relapse[d]
		Cycle 1 Week 1*	Cycle 2 Week 4*	Cycle 3 Week 7*	Possible Cycle 4 Week 10*				
**PHYSICAL**									
History & Physical, PS (w/BP &Weight) [r]	X	X	X	X	X	X		X	X
Adverse Event Notation			X	X	X	X [b]		X [b]	X [b]
Baseline Abnormalities	X	X							
Skin exam [p]	X	X	X	X	X	X [q]		X	
**LABORATORY[e]**									
ANC, platelets, Hgb	X	X	X	X	X	X		X	
Total bilirubin	X	X	X	X	X	X		X	
LDH [f]	X								X
AST and ALT, Alkaline Phosphatase	X	X	X	X	X	X		X	
Serum Creatinine or CrCl	X	X	X	X	X	X		X	
Albumin, Glucose, Na, K, HCO3/CO_2		X	X	X	X	X		X	
Thyroid function test [o]		X		X				X	X
Pregnancy test [i]	X	X	X	X	X	X			

Study Calendar 9.1 continued on next page. Click here for footnotes.

CLOSED EFFECTIVE 06/04/2021

REQUIRED STUDIES	Pre-study [t] (screening)	ON TREATMENT Each cycle consists of 1 infusion. Infusions to be given q 3 weeks. [a q]				End of treatment assessment[u]	Surgical resection [s]	Post treatment F/U prior to progression/ relapse[c]	Post treatment F/U post progression/ relapse[d]
		Cycle 1 Week 1 *	Cycle 2 Week 4 *	Cycle 3 Week 7 *	Possible Cycle 4 Week 10				
**X-RAYS AND SCANS**									
CT [j], chest, abdomen & pelvis or whole body PET/CT	X				X [q]	X [k q]		X [k]	X [v]
EKG[h]	X								
Brain MRI or CT with contrast [h]	X								
**SPECIMEN SUBMISSION**									
Tissue [X]	X		X				X		X (optional)
Whole Blood [w]	X								
**TREATMENT**									
Pembrolizumab (MK-3475) [a]		X	X	X	X[q]				

Click here for **footnotes**.

NOTE: Forms are found on the protocol abstract page of the SWOG website (www.swog.org). Form submission guidelines are found in Section 14.0.

NOTE: Unless indicated otherwise in the protocol, scheduled procedures and assessments (treatment administration, toxicity assessment for continuous treatment, disease assessment, specimen collection and follow-up activities) must follow the established SWOG guidelines as outlined in https://swog.org/Visitors/Download/QA/Best%20Practices%20upddate.pdf.

CLOSED EFFECTIVE 06/04/2021

Footnotes for Study Calendar 9.1: Pembrolizumab (MK-3475)

a. Treatment will continue every 3 weeks, with scans after 3 cycles (9 weeks).
b. Adverse Event Assessments on the study will continue for all patients until 30 days after the last study drug administration. However, patients with ongoing toxicities should be seen more often as clinically indicated.
c. Post-treatment (after receiving the final dose of pembrolizumab on study) follow-up (prior to progression/relapse): Patients should be seen at 6 weeks (-/+ 1 week) after the last dose, then every 12 weeks (+/- 2 weeks) to the end of the first year after registration, then every 6 months (+/- 4 weeks) to the end of the fifth year after registration.
d. After progression patients should be followed every 6 months for up to 2 years from the date of treatment registration, then annually thereafter until 5 years from treatment registration.
e. While on study, blood samples collected for pretreatment laboratory tests may be collected and analyzed no more than 3 days prior to dosing. NOTE: if MK-3475 was delayed per the dose delay/scheduling criteria or if a visit had to be delayed due to major circumstances (such as a health emergency, family/personal emergency, transportation difficulties, scheduling difficulties; a visit date falls on a holiday), the study assessments scheduled for these dates will be delayed.
f. LDH to be done at screening and at progression/relapse. If patient has a subsequent scan 4 weeks later that does not document progression, following an initial progression while on protocol therapy do not need to have LDH done at this time.
h. ECG to be performed at baseline, then as clinically indicated.
i. Women of childbearing potential must have a negative serum or urine pregnancy test within 28 days prior to registration. Following randomization, serum or urine pregnancy tests are required within 72 hours prior to the first dose of study treatment, then according to institutional practice during therapy, tests should coincide with clinic visits for blood work and ending with the discontinuation of study treatment. At discontinuation, a negative pregnancy test within the preceding 6 weeks is sufficient. A pregnancy test should be done at any time there are clinical concerns for possible pregnancy.
j. Patients with primary melanoma of the head and neck will also require a neck CT.
k. For patients who have not progressed/relapsed, disease assessments by CT (or MRI if CT cannot be done) chest/abdomen/pelvis or whole body PET-CT will be performed at Week 9 (at end of neoadjuvant therapy) and then imaging studies may be done as clinically indicated at the discretion of the treating investigator.
o. Thyroid function tests to include TSH, Free T3, and Free T4 must be performed prior to initiating treatment (within 3 days prior to initial dose on Cycle 1) as a reference, should hypothyroidism symptoms arise during treatment. TSH is to be performed prior to dose at week 7, and at initial post treatment follow-up. Additional testing of Free T3 and Free T4 should be performed as clinically indicated if TSH is abnormal or if symptoms are suggestive of thyroid dysfunction.
p. If a skin lesion is a site of disease, photographs (with a ruler in the photo if the lesion is measurable per RECIST 1.1) must be taken as documentation. Physician's report and photograph must be stored in the patient's chart as source documentation. In addition, the physician's report must be uploaded into Medidata Rave (see Section 14.0). In addition, abnormal lesions concerning for basal cell or squamous cell should be documented in the physician's report and, if biopsy obtained, the pathology report should be included in the patient's chart as source documentation.
q. See the third bullet under Section 7.1b. If after the first disease assessment, the tumor is unresectable, treatment will continue for 1 more cycle and a repeat disease assessment using the same technique as baseline will be obtained 4 weeks later. If at this time the patient's disease is deemed resectable, the patient must continue with resection as previously planned.
r. History prior to each treatment initiation to include a menstrual, sexual and contraceptive use history, including date of last menstrual period (for women of childbearing potential) which will help determine the need for pregnancy testing. Similar questions regarding contraceptive use should be asked of men who are sexually active with women of childbearing potential. Height is only required at baseline.

CLOSED EFFECTIVE 06/04/2021

SWOG Authorized Document

Footnotes for Study Calendar 9.1 (contd.): Pembrolizumab (MK-3475)

s. As the primary endpoint of the study is pCR and the goal of the surgery is to remove visible and microscopic disease, surgery is required for all potentially resectable tumors. If the tumor is deemed unresectable at 9 weeks, a biopsy should be obtained.
t. Pre-study labs, MRI brain, and ECG should be obtained within 28 days prior to registration.
u. End of treatment assessment is defined as the first clinical assessment after completion of all pembrolizumab treatments (treatment stopped in preparation for surgical resection). If treatment stopped due to progression, the F/U post progression column would apply. If treatment stopped due to toxicity, the F/U prior to progression would apply.
v. Survival data are to be collected. Scans obtained in post-treatment F/U post progression, are at the clinical discretion of the treating physician and do not need to be submitted into the EDC system.
w. Whole blood must be collected prior to protocol treatment (mandatory), but after protocol registration.
x. Please see Section 15.1 for FFPE submission for translational medicine and Section 15.2 for submission of slides for pathology review.
* These are the corresponding weeks if there are no dose delays.

CLOSED EFFECTIVE 06/04/2021

SWOG Authorized Document

9.2 Pembrolizumab (MK-3475)-Cohort B

REQUIRED STUDIES	Pre-study (screening [s])	ON TREATMENT Each cycle consists of 1 infusion. Infusions to be given q 3 weeks. [a q]				Post treatment F/U prior to progression [c]	Post treatment F/U post progression [d]
		Cycle 1 Week 1 *	Cycle 2 W4 *	Cycle 3 W7*	Cycle 4+		
**PHYSICAL**							
History & Physical, PS (w/BP, Weight)[r]	X	X	X	X	X	X	X
Adverse Event Notation [b]		X	X	X	X	X	X
Skin exam [p]	X	X	X	X	X [q]	X [q]	
Baseline Abnormalities	X	X					
**LABORATORY[e]**							
ANC, platelets, Hgb	X	X	X	X	X	X	
Total bilirubin	X	X	X	X	X	X	
LDH [f]	X						X
AST, ALT, and Alkaline Phosphatase	X	X	X	X	X	X	
Serum Creatinine or CrCl	X	X	X	X	X	X	
Albumin, Glucose, Na, K, HCO3/CO_2		X	X	X	X	X	
Thyroid function tests [o]		X		X	X[o]	X[o]	X
Pregnancy Test[i]	X	X	X	X	X		X

Click here for **footnotes**.

Study Calendar 9.2 continued on next page.

CLOSED EFFECTIVE 06/04/2021

SWOG Authorized Document

REQUIRED STUDIES	Pre-study [s] (Screening)	ON TREATMENT Each cycle consists of 1 infusion. Infusions to be given q 3 weeks. [a q]				Post treatment F/U prior to progression [c]	Post treatment F/U post progression [d]
		Cycle 1 W 1 *	Cycle 2 W 4 *	Cycle 3 W 7 *	Cycle 4+ [a]		
**PHYSICAL**							
**X-RAYS AND SCANS**							
CT [j], chest, abdomen & pelvis or whole body PET/CT [k]	X				X[y]	X [k]	X [t]
Brain MRI or CT with contrast [k h]	X						
EKG [h]	X						
**SPECIMEN SUBMISSION**							
Tissue [w]	X		X				X (optional)
Whole Blood [u]	X						
**TREATMENT**							
Pembrolizumab (MK-3475)		X	X	X	X		

Click here for footnotes.

NOTE: Forms are found on the protocol abstract page of the SWOG website (www.swog.org). Form submission guidelines are found in Section 14.0.

NOTE: Unless indicated otherwise in the protocol, scheduled procedures and assessments (treatment administration, toxicity assessment for continuous treatment, disease assessment, specimen collection and follow-up activities) must follow the established SWOG guidelines as outlined in https://swog.org/Visitors/Download/QA/Best%20Practices%20upddate.pdf.

CLOSED EFFECTIVE 06/04/2021

Footnotes for Study Calendar 9.2: Pembrolizumab (MK-3475)

a. Treatment will continue every 3 weeks, with scans after each 3 cycles (9 weeks). Protocol treatment and parameters will continue on the same schedule until patient has met any of the criteria in Section 7.5. If disease becomes resectable, see surgical guidelines under Section 7.2 and also 14.4h.
b. Adverse Event Assessments on the study will continue for all patients until 30 days after the last study drug administration. However, patients with ongoing toxicities should be seen more often as clinically indicated.
c. Post-treatment follow-up (prior to progression/relapse): Patients should be seen at 6 weeks (-/+ 1 week) after the last dose, then every 12 weeks (+/- 2 weeks) to the end of the year after registration, then every 6 months (+/- 4 weeks) to the end of the fifth year after registration.
d. After progression patients should be followed every 6 months for up to 2 years from the date of treatment registration, then annually thereafter until 5 years from treatment registration.
e. While on study, blood samples collected for pretreatment laboratory tests may be collected and analyzed no more than 3 days prior to dosing. NOTE: if MK-3475 was delayed per the dose delay/scheduling criteria or if a visit had to be delayed due to major circumstances (such as a health emergency, family/personal emergency, transportation difficulties, scheduling difficulties; a visit date falls on a holiday), the study assessments scheduled for these dates will be delayed.
f. LDH to be done at screening and at progression/relapse. If patient has a subsequent scan 4 weeks later that does not document progression, following an initial progression while on protocol therapy, they do not need to have LDH done at this time.
h. To be performed at baseline, then as clinically indicated throughout treatment.
i. Women of child bearing potential must have a negative serum or urine pregnancy test within 28 days prior to registration. Following randomization, serum or urine pregnancy tests are required within 72 hours prior to the first dose of study treatment, then according to institutional practice during therapy, tests should coincide with clinic visits for blood work and ending with the discontinuation of study treatment. At discontinuation, a negative pregnancy test within the preceding 6 weeks is sufficient. A pregnancy test should be done at any time there are clinical concerns for possible pregnancy.
j. Patients with primary melanoma of the head and neck will also require a neck CT.
k. Disease assessments by CT (or MRI if CT cannot be done) chest/abdomen/pelvis or whole body PET/CT will be performed every 9 weeks until disease progression. During treatment and follow-up, brain MRI/CT or other imaging studies may be done as clinically indicated but are not required in the absence of clinical indications.
o. Thyroid function tests to include TSH, Free T3, and Free T4 must be performed prior to initiating treatment (within 3 days prior to initial dose on Cycle 1) as a reference, should hypothyroidism symptoms arise during treatment. TSH is to be performed prior to dose at week 7, and at initial post treatment follow-up. Additional testing of Free T3 and Free T4 should be performed as clinically indicated if TSH is abnormal or if symptoms are suggestive of thyroid dysfunction.
p. If a skin lesion is a site of measurable disease, photographs (with a ruler in the photo if the lesion is measurable per RECIST 1.1) must be taken as documentation. Physician's report and photograph must be stored in the patient's chart as source documentation. In addition, the physician's report must be uploaded into Medidata Rave (see Section 14.0). In addition, abnormal lesions concerning for basal cell or squamous cell should be documented in the physician's report and, if biopsy obtained, the pathology report should be included in the patient's chart as source documentation. If the skin lesion is the site of measurable disease, measurements should be taken after 3 cycles (9 week intervals).
q Patients may continue protocol treatment following progression if they are still deriving clinical benefit in the opinion of the treating investigator.
r. History prior to each treatment initiation to include a menstrual, sexual and contraceptive use history, including date of last menstrual period (for women of childbearing potential) which will help determine the need for pregnancy testing. Similar questions regarding contraceptive use should be asked of men who are sexually active with women of childbearing potential. Height is only required at baseline.
s. Pre-study labs, MRI brain, and ECG should be obtained within 28 days prior to registration.
t. Survival data are to be collected. Scans obtained in post-treatment F/U post progression, are at the clinical discretion of the treating physician and do not need to be submitted into the EDC system.
u. Whole blood must be collected prior to protocol treatment (mandatory).
w. Please see Section 15.1 for FFPE submission for translational medicine and Section 15.2 for additional submission of slides for pathology review.
y. Scans will be obtained every 9 weeks for the first 6 months, then every 4 months.
* These are the corresponding weeks if there are no dose delays.

CLOSED EFFECTIVE 06/04/2021

SWOG Authorized Document

10.0 CRITERIA FOR EVALUATION AND ENDPOINT ANALYSIS

10.1 Measurability of Lesions

a. **Measurable disease**: Measurable disease is defined differently for lymph nodes compared with other disease and will be addressed in a separate section below.

1. Lesions that can be accurately measured in at least one dimension (longest diameter to be recorded) as ≥ 2.0 cm by chest x-ray, by ≥ 1.0 cm with CT or MRI scans, or ≥ 1.0 cm with calipers by clinical exam. All tumor measurements must be recorded in decimal fractions of centimeters (or millimeters).

   The defined measurability of lesions on CT scan is based on the assumption that CT slice thickness is 0.5 cm or less. If CT scans have slice thickness greater than 0.5 cm, the minimum size for a measurable lesion should be twice the slice thickness.

2. Malignant lymph nodes are to be considered pathologically enlarged and measurable if it measures ≥ 1.5 cm in **SHORT AXIS** (greatest diameter perpendicular to the long axis of the lymph node) when assessed by scan (CT scan slice recommended being no greater than 0.5 cm).

b. **Non-measurable disease**: All other lesions (or sites of disease), including small lesions (longest diameter < 1.0 cm or pathologic lymph nodes with ≥ 1.0 cm to < 1.5 cm short axis), are considered non-measurable disease. Bone lesions, leptomeningeal disease, ascites, pleural/pericardial effusions, lymphangitis cutis/pulmonitis, inflammatory breast disease, and abdominal masses (not followed by CT or MRI), are considered non-measurable as are previously radiated lesions that have not progressed.

c. **Notes on measurability**

1. For CT and MRIs, the same type of scanner should be used and the image acquisition protocol should be followed as closely as possible to prior scans. Body scans should by performed with breath-hold scanning techniques, if possible.

2. PET-CT: At present, the low dose or attenuation correction CT portion of a PET-CT is not always of optimal diagnostic CT quality for use with RECIST measurements. However, if the site can document that the CT performed as part of a PET-CT is of identical diagnostic quality to a diagnostic CT, then the CT portion of the PET-CT can be used for RECIST measurements and can be used interchangeably with conventional CT.

3. Ultrasound: Ultrasound is not useful in assessment of lesion size and should not be used as a method of measurement.

4. Cystic lesions that meet the criteria for radiographically defined simple cysts should not be considered as malignant lesions (neither measurable nor non-measurable) since they are, by definition simple cysts.

5. If a target lesion becomes very small some radiologists indicate that it is too small to measure. If the lesion is actually still present, a default

CLOSED EFFECTIVE 06/04/2021

SWOG Authorized Document

measurement of 0.5 cm should be applied. If the radiologist believes the lesion has gone, a default measurement of 0.0cm should be recorded.

10.2 Objective Status at Each Disease Evaluation

Objective Status is to be recorded at each evaluation. All measurable lesions up to a maximum of 2 lesions per organ 5 lesions in total, representative of all involved organs, should be identified as *target* lesions at baseline. All other lesions (or sites of disease) including any measurable lesions over and above the 5 target lesions should be identified as *non-target* lesions. Measurements must be provided for target measurable lesions, while presence or absence must be noted for non-target measurable and non-measurable disease.

For studies that use disease progression as an endpoint, whole body scanning at specific intervals is necessary to determine that progression is NOT present outside of the "target" areas. Therefore, in these studies it is not acceptable to image only the "target" areas of the body in follow-up scans. For study-specific imaging requirements, see the Study Calendar in Section 9.0.

a. **Complete Response (CR):** Complete disappearance of all target and non-target lesions (with the exception of lymph nodes mentioned below). No new lesions. No disease related symptoms. Any lymph nodes (whether target or non-target) must have reduction in short axis to < 1.0 cm. All disease must be assessed using the same technique as baseline.

b. **Partial Response (PR):** Applies only to patients with at least one measurable lesion. Greater than or equal to 30% decrease under baseline of the sum of appropriate diameters of all target measurable lesions. No unequivocal progression of non-measurable disease. No new lesions. All target measurable lesions must be assessed using the same techniques as baseline.

c. **Stable:** Does not qualify for CR, PR, Progression or Symptomatic Deterioration. All target measurable lesions must be assessed using the same techniques as baseline.

d. **Progression**: One or more of the following must occur: 20% increase in the sum of appropriate diameters of target measurable lesions over smallest sum observed (over baseline if no decrease during therapy) using the same techniques as baseline, as well as an absolute increase of at least 0.5 cm. Unequivocal progression of non-measurable disease in the opinion of the treating physician (an explanation must be provided). Appearance of any new lesion/site. Death due to disease without prior documentation of progression and without symptomatic deterioration (see Section 10.2e).

Notes regarding new lesions: FDG-PET imaging can complement regular scans in identifying new lesions according to the following algorithm.

1. Negative FDG-PET at baseline, with a positive FDG-PET at follow-up is a sign of progression based on a new lesion.

2. No FDG-PET at baseline and a positive FDG-PET at follow-up corresponding to a potential new site of disease must have a confirmation by anatomical assessment (e.g., CT, MRI, x-ray) as new site of disease to be considered progressive disease. In such a case, the date of progressive disease will be the date of the initial abnormal FDG-PET.

CLOSED EFFECTIVE 06/04/2021

SWOG Authorized Document

e. **Symptomatic deterioration**: Global deterioration of health status requiring discontinuation of **treatment** without objective evidence of progression. Efforts should be made to obtain objective evidence of progression after discontinuation.

f. **Assessment inadequate, objective status unknown**. Progression or symptomatic deterioration has not been documented, and one or more target measurable lesions have not been assessed or inconsistent assessment methods were used.

g. **Objective status notes:**

1. Non-measurable and non-target measurable disease do not affect Objective Status in determination of CR (must be absent--a patient who otherwise has a CR, but who has non-measurable or non-target measurable disease present or not assessed, will be classified as having a PR). However, non-measurable and non-target lesions are included in determination of progression (if new sites of disease develop or if unequivocal progression occurs in the opinion of the treating physician).

2. An objective status of PR or stable cannot follow one of CR. Stable can follow PR only in the rare case that tumor increases too little to qualify as progression, but enough that a previously documented 30% decrease no longer holds.

3. In cases for which initial flare reaction is possible (hypercalcemia, increased bone pain, erythema of skin lesions), objective status is not progression unless either symptoms persist beyond 4 weeks or there is additional evidence of progression.

4. Lesions that appear to increase in size due to presence of necrotic tissue will not be considered to have progressed.

5. For bone disease documented on bone scan only, increased uptake does not constitute unequivocal progression. However, increase in the soft tissue component of a lesion as measured by CT or MRI would constitute progression.

6. Appearance of new pleural effusions does not constitute unequivocal progression unless cytologically proven of neoplastic origin, since some effusions are a toxicity related to therapy or other medical conditions. Increase in the size of an existing effusion does not constitute unequivocal progression, since the fluid status of the patient could alter the size of the effusion.

7. If CR determination depends on a lesion for which the status is unclear by the required tests, it is recommended the residual lesion be investigated with biopsy or fine needle aspirate.

10.3 Best Response

This is calculated from the sequence of objective statuses.

a. CR: Two or more objective statuses of CR a minimum of four weeks apart documented before progression or symptomatic deterioration.

CLOSED EFFECTIVE 06/04/2021

SWOG Authorized Document

b. PR: Two or more objective statuses of PR or better a minimum of four weeks apart documented before progression or symptomatic deterioration, but not qualifying as CR.

c. Unconfirmed CR: One objective status of CR documented before progression or symptomatic deterioration but not qualifying as CR or PR.

d. Unconfirmed PR: One objective status of PR documented before progression or symptomatic deterioration but not qualifying as CR, PR or unconfirmed CR.

e. Stable/no response: At least one objective status of stable/no response documented at least 6 weeks after registration and before progression or symptomatic deterioration, but not qualifying as anything else above.

f. Increasing disease: Objective status of progression within 12 weeks of registration, not qualifying as anything else above.

g. Symptomatic deterioration: Objective status of symptomatic deterioration within 12 weeks of registration, not qualifying as anything else above.

Inadequate assessment, response unknown: Progression or symptomatic deterioration greater than 12 weeks after registration and no other response category applies.

10.4 Performance Status

Patients will be graded according to the Zubrod Performance Status Scale.

POINT	DESCRIPTION
0	Fully active, able to carry on all pre-disease performance without restriction.
1	Restricted in physically strenuous activity but ambulatory and able to carry out work of a light or sedentary nature, e.g., light housework, office work.
2	Ambulatory and capable of self-care but unable to carry out any work activities; up and about more than 50% of waking hours.
3	Capable of limited self-care, confined to bed or chair more than 50% of waking hours.
4	Completely disabled; cannot carry on any self-care; totally confined to bed or chair.

10.5 Progression-Free Survival

From date of registration to date of first documentation of progression or symptomatic deterioration (see Section 10.2e), or death due to any cause. Patients last known to be alive without report of progression are censored at date of last contact.

CLOSED EFFECTIVE 06/04/2021

SWOG Authorized Document

10.6 Time to Death

From date of registration to date of death due to any cause. Patients last known to be alive are censored at date of last contact.

10.7 Pathologic Complete Response

No evidence of viable tumor cells on complete pathological evaluation of the surgical specimen per institutional standard of care.

11.0 STATISTICAL CONSIDERATIONS

11.1 Cohort A

a. Cohort A will use a single stage design with an accrual goal of 25 eligible patients in total. The anticipated accrual rate for Cohort A is 2 patients per month. Assuming an ineligibility rate of 20%, we will accrue 30 total patients to enroll 25 eligible patients.

b. Primary Endpoint:

The primary endpoint for Cohort A is pathologic complete response rate (pCR) as defined in Section 10.7. All patients who begin neoadjuvant therapy with pembrolizumab will be included in the analysis. Patients who are unable to have all sites of disease completely resected and patients who do not undergo surgery on protocol (for any reason) will be counted as non-responders.

This is a single stage design with the assumption that a true pCR of 25% or higher indicates that the treatment is of considerable interest for patients with resectable DM whereas a true pCR of 5% or lower indicates that the treatment will not be of further interest. Specifically, we will enroll 25 eligible patients with resectable DM. Four or more pathologic complete responses out of 25 would be considered evidence that the treatment warrants further study, provided other factors such as toxicity and overall survival also appear favorable. This design has an alpha of 3.4% (i.e. probability of declaring the regimen warrants further study when the true CR is 5%) and a power of 90% (probability of declaring the regimen warrants further study when the true pCR is 25%).

c. Secondary Endpoints

For the secondary endpoints, 25 eligible patients with measurable disease will be sufficient to estimate the overall response rate (ORR) at 9 weeks within ±19.6% (95% confidence interval). If only half of patients have measurable disease (13), then ORR can be estimated to within 27.2% (95% confidence Interval). Twenty-five eligible patients will also be sufficient to estimate the OS rate at a particular time point (e.g. 9 weeks) to within ±19.6% (95% confidence interval).

CLOSED EFFECTIVE 06/04/2021

SWOG Authorized Document

d. Other Objectives:

Toxicity will be assessed across all patients receiving pembrolizumab. With 25 patients, any toxicity with at least 5% chance of occurring has 72.3% chance of being observed at least once. For any toxicities, the study will estimate the rate of occurrence to within 19.6% (95% confidence interval).

To assess the hypothesis that higher mutational load is associated with pCR rate, the study will assess the association between overall mutational load and pCR rate. We will first calculate the per megabase mutation rate for each individual. Then we will use logistic regression to regress pCR status on the mutation rates and use a 1-degree freedom likelihood ratio test to obtain a p-value for the association between response and mutation rate. We will control the type I error at the two-sided alpha=0.05 level. We estimated the minimal detectable odds-ratio under the assumption that we will collect exome sequencing data on 25 patients and that we will control the type I error rate at the two-sided alpha=0.05 level. In prior data, the median number of mutations per megabase in desmoplastic melanoma was found to be 62 and the 25th and 75th percentiles were 33 and 101, respectively (private communication, Boris Bastian to Antoni Ribas, 11/18/2014). Therefore, by matching the quantiles, we approximate the distribution of the per megabase mutation rate across patients as a Weibull with shape parameter 1.4 and scale parameter 80. Assuming a baseline response rate of 5%, we anticipate over 80% power to detect a log odds-ratio of 0.31 (corresponding to an odds-ratio of 1.36) for each 10 mutation increase in the per megabase mutation rate. If logistic regression is unstable or behaves poorly, e.g. due to low response rates, we will also consider robust and flexible alternatives such as the Wilcoxon test to compare mutation rates between responders and non-responders.

The study will examine whether change in T-cell infiltration following treatment is higher in the DM patients who respond. Specifically, we will compute the change in CD8 expression from baseline to resection and then compare the change between responders and non-responders using a two-sample t-test. We will control type I error at the alpha=0.05 level. Assuming that 25% of patients respond, then we anticipate 80% power to detect a difference in mean CD8 expression change 1.35 standard deviations. We will repeat the analysis comparing the change in CD8 expression from baseline to week 4 to determine whether T-cell infiltration in the on-treatment biopsy is associated with response at resection.

To assess the hypothesis that ctDNA fraction at baseline and week 4 are associated with response in DM patients, we will assess the association between response and ctDNA fraction using a two-sample t-test at the two-sided α=0.05 level. Assuming that 25% of patients respond, then we anticipate 80% power to detect a difference in mean ctDNA fraction of 1.35 standard deviations.
To assess the hypothesis that TCR clonality is higher in DM patients who respond, suggesting that the T-cells are targeting the tumor, we will compute the clonality metric as the normalized Shannon entropy for all patients at baseline, Week 4, and resection. We will then compute the change in clonality metric from baseline to resection. The change in clonality will be compared between responders and non-responders using a two-sample t-test with significance determined at the two-sided alpha=0.05 level. Assuming that 25% of patients respond, then we anticipate 80% power to detect a difference in clonality change of 1.35 standard deviations. We will repeat the analysis comparing the change in clonality from baseline to week 4 to determine whether T-cell clonality in the on-treatment biopsy is associated with response at resection.

CLOSED EFFECTIVE 06/04/2024

SWOG Authorized Document

The study will examine adaptive immune resistance by first evaluating whether PD-L1 expression is increased at Week 4 and resection as compared to baseline using paired t-tests and controlling the type I error at the two-sided alpha=0.05 level. With 25 patients, we anticipate 80% power at each time point to detect a mean difference in expression of 0.58 standard deviations. We will further evaluate whether increased PD-L1 expression at Week 4 or at resection is associated with response. Specifically, we will compute the change in expression from baseline to resection and then compare the change between responders and non-responders using a two-sample t-test. We will control type I error at the two-sided alpha=0.05 level. Assuming that 25% of patients respond then at each time point we anticipate 80% power to detect a difference in mean PD-L1 expression change of 1.35 standard deviations. We will repeat the analysis comparing the change in PD-L1 expression from baseline to week 4 to determine whether T-cell infiltration in the on-treatment biopsy is associated with response at resection. In addition to our assessment of overall mutational load, we will further conduct a neoantigen analysis. Specifically, we will predict the immunogenicity of each nonsynonymous somatic mutation using NetMHC. We will then use logistic regression to regress pCR status on the number of predicted immunogenic mutations, computing a p-value using a 1-df test and controlling type I error at the two-sided alpha=0.05 level.

11.2 Cohort B

a. As a pilot study, Cohort B will use a single stage design with an accrual goal of 21 eligible patients. The anticipated accrual rate for Cohort B is 1 patient per month. Assuming an ineligibility rate of 20%, the study will accrue 26 total patients to enroll 21 eligible patients.

b. Primary Endpoint

The primary endpoint for Cohort B is complete response rate (CR). Given that Cohort B is intended as a pilot trial, we propose single stage design with the assumption that a true CR of 20% or higher indicates that the treatment is of considerable interest for patients with unresectable/metastatic DM whereas a true CR of 5% or lower indicates that the treatment will not be of further interest. Specifically, we will enroll 21 eligible patients with unresectable/metastatic DM. Three or more complete responses out of 21 would be considered evidence that the treatment warrants further study, provided other factors such as toxicity and overall survival also appear favorable. This design has an alpha of 8.5% (i.e. probability of declaring the regimen warrants further study when the true CR is 5%) and a power of 82% (probability of declaring the regimen warrants further study when the true CR is 20%). The higher alpha and lower power used in this design reflect the intention to use this as a pilot trial.

c. Secondary Endpoints

For the secondary endpoints, 21 eligible patients will be sufficient to estimate the PFS and OS at a particular time point (e.g. 9 weeks) to within ±21.4% (95% confidence interval). However, since PFS is estimated only among patients who do not undergo resection, we will only be able to estimate the PFS to within ±21.9%, ±22.5%, or ±23.9% (95% confidence intervals) if the percentage of patients who undergo resection is 5%, 10%, or 20%, respectively.

d. Translational Medicine Objectives: The statistical analysis of the translational medicine objectives for Cohort B will be the same as for Cohort A except we will use CR as the endpoint instead of pCR.

CLOSED EFFECTIVE 06/04/2024

SWOG Authorized Document

To assess the hypothesis that higher mutational load is associated with CR rate, the study will assess the association between overall mutational load and CR rate. We will first calculate the per megabase mutation rate for each individual. Then we will use logistic regression to regress CR status on the mutation rates and use a 1-degree freedom likelihood ratio test to obtain a p-value for the association between response and mutation rate. We will control the type I error at the two-sided alpha=0.05 level. We estimated the minimal detectable odds-ratio under the assumption that we will collect exome sequencing data on 21 patients and that we will control the type I error rate at the two-sided alpha=0.05 level. In prior data, the median number of mutations per megabase in desmoplastic melanoma was found to be 62 and the 25th and 75th percentiles were 33 and 101, respectively (private communication, Boris Bastian to Antoni Ribas, 11/18/2014). Therefore, by matching the quantiles, we approximate the distribution of the per megabase mutation rate across patients as a Weibull with shape parameter 1.4 and scale parameter 80. Assuming a baseline response rate of 5%, we anticipate over 70% power to detect a log odds-ratio of 0.45 (corresponding to an odds-ratio of 1.57) for each 10 mutation increase in the per megabase mutation rate. If logistic regression is unstable or behaves poorly, e.g. due to low response rates, we will also consider robust and flexible alternatives such as the Wilcoxon test to compare mutation rates between responders and non-responders.

The study will examine whether change in T-cell infiltration following treatment is higher in the DM patients who respond. Specifically, we will compute the change in CD8 expression from baseline to progression and then compare the change between responders and non-responders using a two-sample t-test. We will control type I error at the alpha=0.05 level. Assuming that 20% of patients respond, then we anticipate 80% power to detect a difference in mean CD8 expression change 1.61 standard deviations. We will repeat the analysis comparing the change in CD8 expression from baseline to week 4 to determine whether T-cell infiltration in the on-treatment biopsy is associated with response at progression.

To assess the hypothesis that ctDNA fraction at baseline and week 4 are associated with response in DM patients, we will assess the association between response and ctDNA fraction using a two-sample t-test at the two-sided α=0.05 level. Assuming that 20% of patients respond, then we anticipate 80% power to detect a difference in mean ctDNA fraction of 1.61 standard deviations.

To assess the hypothesis that TCR clonality is higher in DM patients who respond, suggesting that the T-cells are targeting the tumor, we will compute the clonality metric as the normalized Shannon entropy for all patients at baseline, Week 4, and progression. We will then compute the change in clonality metric from baseline to progression. The change in clonality will be compared between responders and non-responders using a two-sample t-test with significance determined at the two-sided alpha=0.05 level. Assuming that 20% of patients respond, then we anticipate 80% power to detect a difference in clonality change of 1.61 standard deviations. We will repeat the analysis comparing the change in clonality from baseline to Week 4 to determine whether T-cell clonality in the on-treatment biopsy is associated with response at progression.

The study will examine adaptive immune resistance by first evaluating whether PD-L1 expression is increased at Week 4 and progression as compared to baseline using paired t-tests and controlling the type I error at the two-sided alpha=0.05 level. With 21 patients, we anticipate 80% power at each time point to detect a mean difference in expression of 0.64 standard deviations. We will further evaluate whether increased PD-L1 expression at Week 4 or at progression is

CLOSED EFFECTIVE 06/04/2024

SWOG Authorized Document

associated with response. Specifically, we will compute the change in expression from baseline to progression and then compare the change between responders and non-responders using a two-sample t-test. We will control type I error at the two-sided alpha=0.05 level. Assuming that 20% of patients respond, then at each time point we anticipate 80% power to detect a difference in mean PD-L1 expression change of 1.61 standard deviations. We will repeat the analysis comparing the change in PD-L1 expression from baseline to Week 4 to determine whether T-cell infiltration in the on-treatment biopsy is associated with response at progression. In addition to our assessment of overall mutational load, we will further conduct a neoantigen analysis. Specifically, we will predict the immunogenicity of each nonsynonymous somatic mutation using NetMHC. We will then use logistic regression to regress CR status on the number of predicted immunogenic mutations, computing a p-value using a 1-df test and controlling type I error at the two-sided alpha=0.05 level.

11.3 Data and Safety Monitoring

While the SWOG DSMC does not monitor non-randomized studies, SWOG has policies and procedures in place to monitor single arm studies. Accrual reports are generated weekly and study-specific accrual monitoring is done by the Study Chair, Study Statistician and the Disease Committee Chair. Reports summarizing adverse events, serious adverse events (SAEs) and treatment administration are provided monthly to the Study Chair and Study Statistician for monitoring. In addition all SAEs, which by definition require expeditious reporting, are reviewed and processed by the Adverse Event Coordinator at the SWOG Operations Office and a physician reviewer based on data provided via the NCI CTEP-AERS system. Cumulative study-specific SAE reports are provided to the Study Chair and Study Statistician upon occurrence of an event. Formal reports summarizing the study are prepared for all SWOG members every 6 months.

12.0 DISCIPLINE REVIEW

12.1 Pathology Review

Desmoplastic melanoma will be defined as: a proliferation of spindle cells in the background of stromal fibrosis. Lesional cells may resemble fibroblasts, but there are scattered cells with hyperchromatic atypical nuclei. These would be S100+ and/or SOX-10+ and negative for HMB45, gp100, tyrosinase, and often negative for Melan-A. After completion of the study, tissues will be reviewed centrally to standardize the pathologic eligibility. (*12,13*,14)

The slides used for the primary diagnosis of desmoplastic melanoma must be submitted for confirmatory review if available. These include the H&E slides and any immunostains completed to diagnose desmoplastic melanoma and to rule out other diagnoses. All possible attempts should be made to obtain these slides.

These specimens will be submitted as outlined in Section 15.2.

13.0 REGISTRATION GUIDELINES

13.1 Registration Timing

Patients must be registered prior to initiation of treatment (no more than nine calendar days prior to planned start of treatment).

CLOSED EFFECTIVE 06/04/2021

SWOG Authorized Document

13.2 Investigator/Site Registration

Prior to the recruitment of a patient for this study, investigators must be registered members of a Cooperative Group. Each investigator must have an NCI investigator number and must maintain an "active" investigator registration status through the annual submission of a complete investigator registration packet to CTEP.

a. CTEP Investigator Registration Procedures

Food and Drug Administration (FDA) regulations and National Cancer Institute (NCI) policy require all investigators participating in any NCI-sponsored clinical trial to register and to renew their registration annually.

Registration requires the submission of:

- a completed ***Statement of Investigator Form*** (FDA Form 1572) with an original signature
- a current Curriculum Vitae (CV)
- a completed and signed ***Supplemental Investigator Data Form*** (IDF)
- a completed ***Financial Disclosure Form*** (FDF) with an original signature

Fillable PDF forms and additional information can be found on the CTEP website at http://ctep.cancer.gov/investigatorResources/investigator_registration.htm.

For questions, please contact the ***CTEP Investigator Registration Help Desk*** by email at pmbregpend@ctep.nci.nih.gov.

b. CTEP Associate Registration Procedures

The Cancer Therapy Evaluation Program (CTEP) Identity and Access Management (IAM) application is a web-based application intended for use by both Investigators (i.e., all physicians involved in the conduct of NCI-sponsored clinical trials) and Associates (i.e., all staff involved in the conduct of NCI-sponsored clinical trials).

Associates will use the CTEP-IAM application to register (both initial registration and annual re-registration) with CTEP and to obtain a user account.

Investigators will use the CTEP-IAM application to obtain a user account only. (See CTEP Investigator Registration Procedures above for information on registering with CTEP as an Investigator, which must be completed before a CTEP-IAM account can be requested.)

An active CTEP-IAM user account will be needed to access all CTEP and CTSU (Cancer Trials Support Unit) websites and applications, including the CTSU members' website.

Additional information can be found on the CTEP website at http://ctep.cancer.gov/branches/pmb/associate_registration.htm. For questions, please contact the ***CTEP Associate Registration Help Desk*** by email at ctepreghelp@ctep.nci.nih.gov.

c. CTSU Registration Procedures

This study is supported by the NCI Cancer Trials Support Unit (CTSU).

CLOSED EFFECTIVE 06/04/2021

SWOG Authorized Document

IRB Approval:

Each investigator or group of investigators at a clinical site must obtain IRB approval for this protocol and submit IRB approval and supporting documentation to the CTSU Regulatory Office before they can be approved to enroll patients. Assignment of site registration status in the CTSU Regulatory Support System (RSS) uses extensive data to make a determination of whether a site has fulfilled all regulatory criteria including but not limited to: an active Federal Wide Assurance (FWA) number, an active roster affiliation with the Lead Network or a participating organization, a valid IRB approval, and compliance with all protocol specific requirements.

Sites participating on the NCI CIRB initiative that are approved by the CIRB for this study are not required to submit IRB approval documentation to the CTSU Regulatory Office. For sites using the CIRB, IRB approval information is received from the CIRB and applied to the RSS in an automated process. Signatory Institutions must submit a Study Specific Worksheet for Local Context (SSW) to the CIRB via IRBManager to indicate their intent to open the study locally. The CIRB's approval of the SSW is then communicated to the CTSU Regulatory Office. In order for the SSW approval to be processed, the Signatory Institution must inform the CTSU which CIRB-approved institutions aligned with the Signatory Institution are participating in the study.

Downloading Site Registration Documents:

Site registration forms may be downloaded from the **S1512** protocol page located on the CTSU members' website. Permission to view and download this protocol and its supporting documents is restricted and is based on person and site roster assignment housed in the CTSU RSS.

- Go to https://www.ctsu.org and log in to the members' area using your CTEP-IAM username and password
- Click on the Protocols tab in the upper left of your screen
- Either enter the protocol # in the search field at the top of the protocol **S1512** tree, or
- Click on the By Lead Organization folder to expand
- Click on the SWOG link to expand, then select trial protocol **S1512**
- Click on LPO Documents, select the Site Registration documents link, and download and complete the forms provided.

Requirements For S1512 Site Registration:

- CTSU Transmittal Sheet (optional)
- IRB approval (For sites not participating via the NCI CIRB; local IRB documentation, an IRB-signed CTSU IRB Certification Form, Protocol of Human Subjects Assurance Identification/IRB Certification/Declaration of Exemption Form, or combination is accepted)

Submitting Regulatory Documents:

Submit required forms and documents to the CTSU Regulatory Office via the Regulatory Submission Portal, where they will be entered and tracked in the CTSU RSS.

Regulatory Submission Portal: www.ctsu.org (members' area) → Regulatory Tab → Regulatory Submission

CLOSED EFFECTIVE 06/04/2021

SWOG Authorized Document

When applicable, original documents should be mailed to:

CTSU Regulatory Office
1818 Market Street, Suite 1100
Philadelphia, PA 19103

Institutions with patients waiting that are unable to use the Portal should alert the CTSU Regulatory Office immediately at 866-651-2878 in order to receive further instruction and support.

Checking Your Site's Registration Status:

You can verify your site registration status on the members' section of the CTSU website.

- Go to https://www.ctsu.org and log in to the members' area using your CTEP-IAM username and password
- Click on the Regulatory tab at the top of your screen
- Click on the Site Registration tab
- Enter your 5-character CTEP Institution Code and click on Go

Note: The status given only reflects compliance with IRB documentation and institutional compliance with protocol-specific requirements as outlined by the Lead Network. It does not reflect compliance with protocol requirements for individuals participating on the protocol or the enrolling investigator's status with the NCI or their affiliated networks.

13.3 OPEN Registration Requirements

The individual registering the patient must have completed the appropriate SWOG Registration Worksheet. The completed form must be referred to during the registration but should not be submitted as part of the patient data.

Patient enrollment will be facilitated using the Oncology Patient Enrollment Network (OPEN). OPEN is a web-based registration system available on a 24/7 basis. To access OPEN, the site user must have an active CTEP-IAM account (check at < https://eapps-ctep.nci.nih.gov/iam/index.jsp >) and a 'Registrar' role on either the LPO or participating organization roster.

CLOSED EFFECTIVE 06/04/2021

SWOG Authorized Document

OPEN will also ask additional questions that are not present on the SWOG Registration Worksheet. The individual registering the patient must be prepared to provide answers to the following questions:

a. Institution CTEP ID

b. Protocol Number

c. Registration Step

d. Treating Investigator

e. Credit Investigator

f. Patient Initials

g. Patient's Date of Birth

h. Patient SSN (SSN is desired, but optional. Do not enter invalid numbers.)

i. Country of Residence

j. ZIP Code

k. Gender (select one):
- Female Gender
- Male Gender

l. Ethnicity (select one):
- Hispanic or Latino
- Not Hispanic or Latino
- Unknown

m. Method of Payment (select one):
- Private Insurance
- Medicare
- Medicare and Private Insurance
- Medicaid
- Medicaid and Medicare
- Military or Veterans Sponsored NOS
- Military Sponsored (Including Champus & Tricare)
- Veterans Sponsored
- Self Pay (No Insurance)
- No Means of Payment (No Insurance)
- Other
- Unknown

n. Race (select all that apply):
- American Indian or Alaska Native
- Asian
- Black or African American
- Native Hawaiian or other Pacific Islander
- White
- Unknown

CLOSED EFFECTIVE 06/04/2021

SWOG Authorized Document

13.4 Registration Procedures

a. All site staff will use OPEN to enroll patients to this study. OPEN is integrated with the CTSU Enterprise System for regulatory and roster data and, upon enrollment, initializes the patient in the Rave database. OPEN can be accessed at https://open.ctsu.org, from the OPEN tab on the CTSU members' side of the website at https://www.ctsu.org, or from the OPEN Patient Registration link on the SWOG CRA Workbench.

b. Prior to accessing OPEN site staff should verify the following:

- All eligibility criteria have been met within the protocol stated timeframes and the affirmation of eligibility on the Registration Worksheet has been signed by the registering investigator or another investigator designate. Site staff should refer to Section 5.0 to verify eligibility.

- All patients have signed an appropriate consent form and HIPAA authorization form (if applicable).

c. The OPEN system will provide the site with a printable confirmation of registration and treatment information. Please print this confirmation for your records.

d. Further instructional information is provided on the OPEN tab on the CTSU members' side of the website at https://www.ctsu.org or at https://open.ctsu.org. For any additional questions contact the CTSU Help Desk at 888/823-5923 or ctsucontact@westat.com.

13.5 Exceptions to SWOG registration policies will not be permitted.

a. Patients must meet all eligibility requirements.

b. Institutions must be identified as approved for registration.

c. Registrations may not be cancelled.

d. Late registrations (after initiation of treatment) will not be accepted.

14.0 DATA SUBMISSION SCHEDULE

14.1 Data Submission Requirement

Data must be submitted according to the protocol requirements for **ALL** patients registered, whether or not assigned treatment is administered, including patients deemed to be ineligible. Patients for whom documentation is inadequate to determine eligibility will generally be deemed ineligible.

14.2 Master Forms

Master forms can be found on the protocol abstract page on the SWOG website (www.swog.org) and (with the exception of the sample consent form and the Registration Worksheet) must be submitted on-line via the Web; see below for details.

14.3 Data Submission Procedures

CLOSED EFFECTIVE 06/04/2021

SWOG Authorized Document

a. Data collection for this study will be done exclusively through the Medidata Rave® clinical data management system. Access to the trial in Rave is granted through the iMedidata application to all persons with the appropriate roles assigned in Regulatory Support System (RSS). To access Rave via iMedidata, you must have an active CTEP-IAM account (check at https://eapps-ctep.nci.nih.gov/iam/index.jsp) and the appropriate Rave role (Rave CRA, Read-Only, Site Investigator) on either the LPO or participating organization roster at the enrolling site.

Upon initial site registration approval for the study in RSS, all persons with Rave roles assigned on the appropriate roster will be sent a study invitation e-mail from iMedidata. To accept the invitation, site users must log into the Select Login (https://login.imedidata.com/selectlogin) using their CTEP-IAM user name and password, and click on the “accept” link in the upper right-corner of the iMedidata page. Please note, site users will not be able to access the study in Rave until all required Medidata and study specific trainings are completed. Trainings will be in the form of electronic learnings (eLearnings), and can be accessed by clicking on the link in the upper right pane of the iMedidata screen.

Users that have not previously activated their iMedidata/Rave account at the time of initial registration approval for the study in RSS will also receive a separate invitation from iMedidata to activate their account. Account activation instructions are located on the CTSU website, Rave tab under the Rave resource materials (Medidata Account Activation and Study Invitation Acceptance). Additional information on iMedidata/Rave is available on the CTSU members’ website under the Rave tab at www.ctsu.org/RAVE/ or by contacting the CTSU help Desk at 888/823-5923 or by e-mail at ctsucontact@westat.com.

b. You may also access Rave® via the SWOG CRA Workbench. Go to the SWOG web site (http://swog.org) and logon to the Members Area using your SWOG Roster ID Number and password. After you have logged on, click on *Workbenches*, then *CRA Workbench* to access the home page for the CRA Workbench and follow the link to Rave® provided in the left-hand navigation panel.

To access the CRA Workbench the following must be done (in order):

1. You are entered into the SWOG Roster and issued a SWOG Roster ID Number,
2. You are associated as an investigator or CRA/RN at the institution where the patient is being treated or followed,
3. Your Web User Administrator has added you as a web user and has given you the appropriate system permissions to view data for that institution.

For assistance with points 1 and 2 call the Operations Office at 210/614-8808. For point 3, contact your local Web User Administrator (refer to the "Who is my Web User Administrator?" function on the swog.org Members logon page).

For difficulties with the CRA Workbench, please email technicalquestion@crab.org.

c. Institutions participating through the Cancer Trials Support Unit (CTSU), please refer to the CTSU Participation Table.

14.4 Data Submission Overview and Timepoints

a. <u>WITHIN 7 DAYS OF INITIAL REGISTRATION, SUBMIT</u>:

CLOSED EFFECTIVE 06/04/2021

SWOG Authorized Document

S1512 Onstudy Form

S1512 Baseline Abnormalities Form

Baseline Tumor Assessment Form (RECIST 1.1)

Radiology reports from all scans performed to assess disease at baseline**

Physician report documenting any measurement of skin lesions made during a physical examination**

Pathology report documenting histologic confirmation of desmoplastic melanoma**

**NOTE: Upload reports via the Source Documentation: Baseline form in Rave®.

Whole blood specimens must be collected prior to starting protocol treatment and MUST BE SUBMITTED THE SAME DAY AS COLLECTED.

b. WITHIN 28 DAYS OF REGISTRATION:

See Section 15.0 for specimen submission requirements.

c. WITHIN 7 DAYS AFTER EACH CYCLE OF TREATMENT (1 CYCLE = 21 DAYS) SUBMIT**:**

S1512 Treatment Form

S1512 Adverse Event Form

d. FOR COHORT A, WITHIN 7 DAYS AFTER THE END OF TREATMENT DISEASE ASSESSMENT:

Follow-Up Tumor Assessment Form (RECIST 1.1)

Radiology reports from all scans performed to assess disease*. Physician must note tumor measurement in patient records.

Physician report documenting any measurements of skin lesions made during a physical examination.*

*NOTE: Upload reports via the Source Documentation: Follow-Up form in Rave®

e. FOR COHORT B, WITHIN 7 DAYS AFTER EACH DISEASE ASSESSMENT (SEE STUDY CALENDAR FOR SCHEDULE) UNTIL PROGRESSION* (AS DEFINED IN SECTION 10.2d), SUBMIT:

Follow-Up Tumor Assessment Form (RECIST 1.1)

Radiology reports from all scans performed to assess disease.* Physician must note tumor measurement in patient records.

Physician report documenting any measurements of skin lesions made during a physical examination.*

*NOTE: Upload reports via the Source Documentation: Follow-Up form in Rave®.

CLOSED EFFECTIVE 06/04/2021

SWOG Authorized Document

f. WITHIN 7 DAYS OF DISCONTINUATION OF PROTOCOL TREATMENT, SUBMIT:

Off Treatment Notice

S1512 Treatment Form

S1512 Adverse Event Form.

g. WITHIN 14 DAYS OF PROGRESSION OR RELAPSE, SUBMIT:

Follow-Up Tumor Assessment Form (RECIST 1.1) OR Follow-Up Form (if patient underwent surgery to resect disease after starting protocol therapy).

Radiology reports from all scans performed to assess disease*. Physician must note tumor measurement in patient records.

Physician report documenting any measurements of skin lesions made during a physical examination.*

If patient was on protocol treatment at the time of progression, also submit the materials specified in Section 14.4f.

See Section 15.0 for specimen submission requirements.

*NOTE: Upload reports via the Source Documentation: Follow-Up form in Rave®.

h. WITHIN 14 DAYS OF SURGERY TO RESECT DISEASE, SUBMIT:

S1512 Pathologic Response Form

All operative and pathology reports from the procedure*.

Submit surgical specimen and specimen from biopsy between Weeks 3-5. See Section 15.0 for specimen submission requirements.

*NOTE: Upload reports via the Source Documentation: Follow-Up form in Rave®.

i. ONCE OFF ALL PROTOCOL TREATMENT SUBMIT EVERY 6 MONTHS FOR THE FIRST 2 YEARS, THEN YEARLY FOR A TOTAL OF 5 YEARS FROM INITIAL REGISTRATION:

Follow-Up Form

Late Effects Form (if prior to treatment for progression or relapse or a second primary, and prior to non-protocol treatment, the patient experiences any severe [Grade ≥ 3] long term toxicity that has not been previously reported).

j. WITHIN 28 DAYS OF KNOWLEDGE OF DEATH:

Notice of Death **and all of the items listed in Section 14.4e** (if the patient was still on protocol treatment) or Follow-Up Form (if the patient was off protocol treatment) documenting death information.

CLOSED EFFECTIVE 06/04/2021

SWOG Authorized Document

15.0 SPECIAL INSTRUCTIONS

15.1 Specimen Submission for Translational Medicine

Specimens for Translational Medicine studies outlined in Section 18.1 are required for participation in this study.

a. Specimens must be submitted at the timepoints listed below. Collection instructions are outlined in Section 15.1c and submission instructions are outlined in Section 15.1e. NOTE: With patient consent, leftover specimens will be banked for future unknown use.

b. Specimens must be submitted at the following times (see Section 9.0):

1. Three cores (at least 18 gauge needle) to be obtained from primary (preferred) or metastatic lesions (avoid lymph node biopsy if possible) and made into three different FFPE blocks (one core per block). Sending all three blocks is preferred. Thirty slides (minimum 5um thick) per block from each of the blocks may be submitted if the institution cannot release pathology paraffin embedded blocks, at the following times:
   - Baseline (required) – prior to the start of study treatment. Initial/Archival biopsy may be used if 4 cm x 30 slides can be provided). Submit tissue within 30 days after registration.
   - Biopsy between 3 and 5 weeks after initiation of treatment (required for all patients with residual disease to biopsy). Submit tissue within 30 days of collection.
   - Surgical specimen from each site of surgical resection (required if surgical resection is performed). Submit tissue within 30 days of collection.
   - Progression/relapse (optional). Submit tissue within 30 days of collection.

   Submit formalin fixed paraffin embedded (FFPE) tissue according to guidelines below.

2. Twenty mL whole blood in vacutainer tubes with EDTA coating. Collect at the following times:
   - Baseline (mandatory prior to the start of study treatment)

c. Specimen Collection Instructions

1. Standard collection of Paraffin Embedded Tissue Block.
   - Place the fresh tissue in formalin. Do Not Exceed 24 hours fixation time.
   - Fixed tissue must be paraffin embedded within 24 hours.
   - Send H & E (Hematoxylin and Eosin) stained section if it is routine practice for the site. Otherwise, sending the block without the H&E slide is adequate.

CLOSED EFFECTIVE 06/04/2021

SWOG Authorized Document

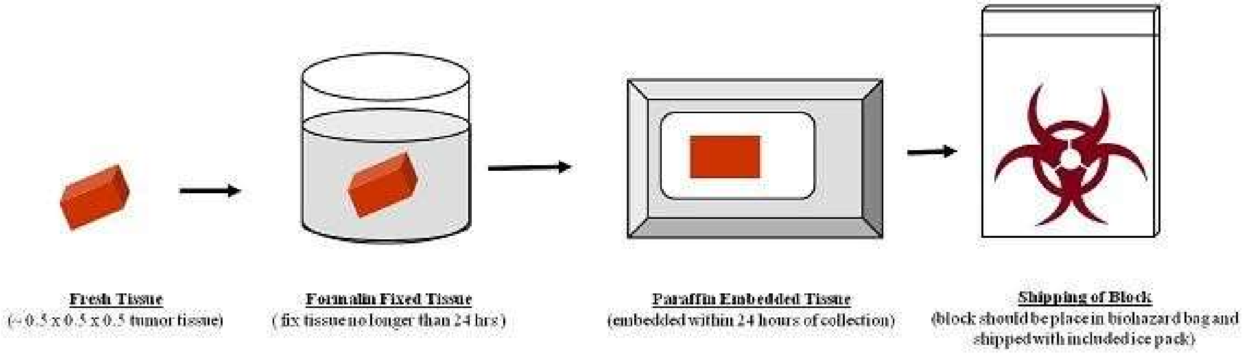

2. Standard collection of Slides.
   - Slides may be processed according to local pathology guidelines.

3. Standard Instructions for Shipping Paraffin Embedded Tissues and Slides:
   - Include in shipment:
     i. Tissue as outlined above
     ii. A pathology report (indicating the morphological diagnosis).
     iii. A copy of the packing/shipping list produced by the SWOG Online Specimen Tracking System.
   - Ship paraffin embedded tissue according to the shipping guidelines for ambient specimens in the General Specimen Submission Instructions below.

NOTE: FFPE tissue blocks will NOT be returned to the submitting institution unless it is required for patient care. If the block is needed for patient care, a written request must be submitted to the Ribas Laboratory with a rationale for the return. If a site will have an issue with this requirement, the option to submit cores in place of the block should be considered.

4. Standard Instructions for Collecting and Shipping Fresh Whole Blood Specimens.
   - Use 10 ml vacutainer tubes with EDTA to collect whole blood.
     - If your site does not have the recommended size of vacutainer tubes required by the treatment protocol, other sized tubes may be used to collect the required/requested amount of blood.
     - Avoid using < 3mL collection tubes.
     - Pre-label vacutainer tube(s) according to specimen labeling requirements indicated in the General Specimen Submission Instructions.
   - Use aseptic techniques and draw blood from the patient into the vacutainer tube(s). The amount of blood required will vary per protocol.
   - Immediately after the blood is drawn, thoroughly mix the blood with the anticoagulant by gently inverting the vacutainer tube(s) multiple times.
   - Store whole blood prior to shipping at room temperature. Do not freeze the whole blood. Storage time longer than 24 hours should be noted on the specimen shipping form.

CLOSED EFFECTIVE 08/04/202

SWOG Authorized Document

- Whole blood should be shipped the same day (day of collection) preferably using Federal Express, Priority Overnight service. Use of other courier services may delay package receipt.
    - **Specimens may be shipped Monday through Thursday using only overnight delivery to arrive Monday through Friday. No blood should be collected or shipped on Friday or right before a holiday. For questions about shipping specimens around a holiday, please reference the Bank's holiday hours memo distributed by the SWOG Operations Office, or call the Ribas Lab directly at 310-825-2676.**
    - During the months of April-September, ship fresh specimens on a cold pack. During the months of October-March, insulate fresh specimens to keep from freezing due to weather (ex. wrap specimen in bubble wrap).

For packaging instructions, refer to the shipping guidelines for ambient specimens in the General Specimen Submission Instructions outlined below.

d. Specimen collection kits are not being provided for this submission; sites will use institutional supplies.

e. SHIPPING SAMPLES

1. All submitted specimens must be labeled with the treatment protocol number, SWOG patient number, patient initials, time point, and date of specimen collection, specimen number and/or specimen type, Surgical Pathology ID (Accession # from pathology report), and block number (as applicable).

a. Instructions for Labels

i. Large Labels:
- Use Avery 5160 labels*
- Use for all specimens submitted
- Type or write information
- Place on outside bag or container, depending on specimen type

ii. Small Labels:
- Use Avery 5160 labels*
- Use for all specimens submitted
- Type or write information on right side of line
- Cut the label on the line
- Place only the right side of the label on each specimen
- For blood products, place on each vial submitted
- For paraffin specimens, place on slides or blocks

***If Avery 5160 labels are not available, the label may be printed on regular paper and taped to the specimen container.**

2. Include a specimen shipping form in every shipment, ensuring the information on the form exactly matches the specimens in the shipment.

CLOSED EFFECTIVE 06/04/2021

SWOG Authorized Document

3. Ship specimens according to IATA shipping regulations.

   a. *Shipping guidelines for ambient specimens:*
      i. The specimen must be wrapped in absorbable material.
      ii. Place the specimen in an AIRTIGHT container (must have a primary and secondary container, ex a Saf-T-Pak).
      iii. Place packaged specimen in an appropriate shipping container (ex. FedEx box or clinical pack).

   Mark the outside shipping container with an "Exempt Human Specimen" label.

4. SWOG Specimen Tracking System (STS)

   All specimen submissions for this study must be entered and tracked using the SWOG online Specimen Tracking system. SWOG members may log on the online system via the CRA Workbench. To access the CRA Workbench, go to the SWOG Web site (http://swog.org) and logon to the Members Area. After you have logged on using your SWOG roster ID number and password, click on the *CRA Workbench* link to access the home page for CRA Workbench website. Non- SWOG users may log into SpecTrack using their CTSU UserID and password on the SpecTrack login page located at https://crawb.crab.org/SpecTrack/Logon.aspx (select the option "SWOG – SWOG – CTSU"). SpecTrack start-up instructions (both written and demo) are available after signing in to SpecTrack.

   A copy of the Shipment Packing List produced by the online Specimen Tracking system should be printed and placed in the pocket of the specimen bag if it has one, or in a separate resealable bag. The Specimen Submission Form is NOT required when the online system is used.

   ALL SPECIMENS MUST BE LOGGED VIA THIS SYSTEM; THERE ARE NO EXCEPTIONS.

   To report technical problems with Specimen Tracking, such as database errors or connectivity issues, please send an email to technicalquestion@crab.org. For procedural help with logging and shipping specimens, there is an introduction to the system on the Specimen Tracking main page (http://dnet.crab.org/SpecTrack/Documents/Instructions.pdf); or contact the Data Operations Center at 206/652-2267 to be routed to the Data Coordinator for further assistance.

CLOSED EFFECTIVE 06/04/2021

SWOG Authorized Document

In the online specimen tracking system, the appropriate SWOG laboratory for submission is identified as follows:

Lab #224: The Ribas Laboratory
Attn: Agustin Vega-Crespo
Medical Receiving – Study **S1512**
CHS 54-200
650 Charles E Young Drive S
UCLA Medical Center
Los Angeles, CA 90095
Phone: 310-825-2676
FAX: 310-825-2493

Contact: Agustin Vega-Crespo
E-mail: avegacrespo@mednet.ucla.edu

ATTENTION: DO NOT SHIP SPECIMENS FROM SECTION 15.1 TO THE SWOG BIOSPECIMEN BANK

5. Federal guidelines for the shipment of blood products:

a. The tube must be wrapped in an absorbent material.

b. The tube must then be placed in an AIRTIGHT container (like a resealable bag).

c. Pack the resealable bag and tube in a Styrofoam shipping container.

d. Pack the Styrofoam shipping container in a cardboard box.

e. Mark the box "Biohazard"

15.2 Specimen Submission for Central Confirmatory Quality Review

Specimens for central review as outlined in Section 12.1 are required (if available) for participation in this study.

Specimen instructions are outlined in Section 15.2a and submission instructions are outlined in Section 15.2b. NOTE: With patient consent, leftover specimens will be banked for future unknown use.

a. Within 120 days after CTSU's distribution of Revision #7, archival slides from the primary diagnosis of desmoplastic melanoma must be submitted for central confirmatory quality review, if available:

1. Slides
- At least one (1) H&E slide representative of the diagnosis
- If available, at least one (1) immunostain, such as gp100, Sox 10, Melan-A, HMB-45, completed from the initial diagnosis of desmoplastic melanoma (the slides that were used to rule out other diagnoses), though all immunostains are preferred. All possible attempts should be made to obtain these slides

CLOSED EFFECTIVE 06/04/2021

SWOG Authorized Document

2. The corresponding anatomic pathology report that includes the diagnosis is required. Label the report with the patient ID# and surgical pathology identification number.

b. SHIPPING CENTRAL CONFIRMATORY QUALITY REVIEW SAMPLES

1. Specimen Labeling

Slides must be labeled with the following:

- SWOG participant number
- Participant initials
- Collection date (date the specimen was collected from the participant)
- Specify whether the tissue is from primary (P), metastatic (M), or normal/uninvolved (N)
- For lymph nodes, indicate it positive (+) or negative (-) for tumor involvement
- Surgical Pathology ID # (Accession#) and block number (e.g., A2, 3E, 2-1, B, etc.) must be on both the specimen label and the pathology report in order for the Bank to adequately match the specimen with any findings in the pathology report

2. For additional information about labeling and shipping instructions (including address) for slides, refer to the SWOG Specimen Submission webpage (https://www.swog.org/clinical-trials/biospecimen-resources/biospecimen-processing-and-submission-procedures).

3. SWOG Specimen Tracking System (STS)

All specimen submissions for this study must be entered and tracked using the SWOG online Specimen Tracking system. SWOG members may log on the online system via the CRA Workbench. To access the CRA Workbench, go to the SWOG Web site (http://swog.org) Non- SWOG users may log into SpecTrack using their CTSU UserID and password on the SpecTrack login page located at https://spectrack.crab.org (select the option “SWOG – SWOG – CTSU”). SpecTrack start-up instructions (both written and demo) are available after signing in to SpecTrack.

A copy of the Shipment Packing List produced by the online Specimen Tracking system should be printed and placed in the pocket of the specimen bag if it has one, or in a separate resealable bag. The patient ID must be included on any source documentation included with the shipment.

ALL SPECIMENS MUST BE LOGGED VIA THIS SYSTEM; THERE ARE NO EXCEPTIONS.

Note: If limited or no tissue is available, then the site must document reason for incomplete specimen submission (insufficient tissue available) in the participant’s medical record and in the SWOG Specimen Tracking System. If limited tissue is available, this must be documented in the Specimen Tracking System under “Comments” at the time of specimen submission. If no tissue available, this must be documented in the

CLOSED EFFECTIVE 06/04/2021

SWOG Authorized Document

Specimen Tracking System by choosing “Notify that Specimen Cannot be Submitted”.

To report technical problems with Specimen Tracking, such as database errors or connectivity issues, please send an email to technicalquestion@crab.org. For procedural help with logging and shipping specimens, there is an introduction to the system on the Specimen Tracking main page (https://spectrack.crab.org/Instructions); or contact the SWOG Statistics and Data Management Center at 206/652-2267 to be routed to the Data Coordinator for further assistance.

In the online specimen tracking system, the appropriate SWOG laboratory for submission of central confirmatory pathology review tissue samples for SWOG Repository Submission is identified as follows:

Lab #201: SWOG Biospecimen Bank
Solid Tissue, Myeloma, and Lymphoma Division
Phone: 614-722-2865
FAX: 614-722-2897
E-mail: bpcbank@nationwidechildrens.org

CLOSED EFFECTIVE 06/04/2021

16.0 ETHICAL AND REGULATORY CONSIDERATIONS

The following must be observed to comply with Food and Drug Administration regulations for the conduct and monitoring of clinical investigations; they also represent sound research practice:

Informed Consent

The principles of informed consent are described by Federal Regulatory Guidelines (Federal Register Vol. 46, No. 17, January 27, 1981, part 50) and the Office for Protection from Research Risks Reports: Protection of Human Patients (Code of Federal Regulations 45 CFR 46). They must be followed to comply with FDA regulations for the conduct and monitoring of clinical investigations.

Institutional Review

This study must be approved by an appropriate institutional review committee as defined by Federal Regulatory Guidelines (Ref. Federal Register Vol. 46, No. 17, January 27, 1981, part 56) and the Office for Protection from Research Risks Reports: Protection of Human Patients (Code of Federal Regulations 45 CFR 46).

Drug Accountability

An investigator is required to maintain adequate records of the disposition of investigational drugs according to procedures and requirements governing the use of investigational new drugs as described in the Code of Federal Regulations 21 CFR 312.

Publication and Industry Contact

The agent(s) supplied by CTEP, DCTD, NCI used in this protocol is/are provided to the NCI under a Collaborative Agreement (CRADA, CTA, CSA) between Merck (hereinafter referred to as "Collaborator(s)") and the NCI Division of Cancer Treatment and Diagnosis. Therefore, the following obligations/guidelines in addition to the provisions in the "Intellectual Property Option to Collaborator"

(http://ctep.cancer.gov/industryCollaborations2/intellectual_property.htm) contained within the terms of award apply to the use of the Agent in this study:

1. Agent(s) may not be used for any purpose outside the scope of this protocol, nor can Agent(s) be transferred or licensed to any party not participating in the clinical study. Collaborator(s) data for Agent(s) are confidential and proprietary to Collaborator(s) and shall be maintained as such by the investigators. The protocol documents for studies utilizing Agents contain confidential information and should not be shared or distributed without the permission of the NCI. If a copy of this protocol is requested by a patient or patient's family member participating on the study, the individual should sign a confidentiality agreement. A suitable model agreement can be downloaded from: http://ctep.cancer.gov.

2. For a clinical protocol where there is an investigational Agent used in combination with (an)other investigational Agent(s), each the patient of different Collaborative Agreements, the access to and use of data by each Collaborator shall be as follows (data pertaining to such combination use shall hereinafter be referred to as "Multi-Party Data"):

   a. NCI will provide all Collaborators with prior written notice regarding the existence and nature of any agreements governing their collaboration with NCI, the design of the proposed combination protocol, and the existence of any obligations which would tend to restrict NCI's participation in the proposed combination protocol.

   b. Each Collaborator shall agree to permit use of the Multi-Party Data from the clinical trial by any other Collaborator solely to the extent necessary to allow said other Collaborator to develop, obtain regulatory approval or commercialize its own Agent.

   c. Any Collaborator having the right to use the Multi-Party Data from these trials must agree in writing prior to the commencement of the trials that it will use the Multi-Party Data solely for development, regulatory approval, and commercialization of its own Agent.

3. Clinical Trial Data and Results and Raw Data developed under a Collaborative Agreement will be made available exclusively to Collaborator(s), the NCI, and the FDA, as appropriate and unless additional disclosure is required by law or court order as described in the IP Option to Collaborator (http://ctep.cancer.gov/industryCollaborations2/intellectual_property.htm). Additionally, all Clinical Data and Results and Raw Data will be collected, used and disclosed consistent with all applicable federal statutes and regulations for the protection of human patients, including, if applicable, the Standards for Privacy of Individually Identifiable Health Information set forth in 45 C.F.R. Part 164.

4. When a Collaborator wishes to initiate a data request, the request should first be sent to the NCI, who will then notify the appropriate investigators (Group Chair for Cooperative Group studies, or PI for other studies) of Collaborator's wish to contact them.

5. Any data provided to the Collaborator(s) for Phase III studies must be in accordance with the guidelines and policies of the responsible Data Monitoring Committee (DMC), if there is a DMC for this clinical trial.

6. Any manuscripts reporting the results of this clinical trial must be provided to CTEP by the Group office for Cooperative Group studies or by the principal investigator for non-Cooperative Group studies for immediate delivery to Collaborator(s) for advisory review and comment prior to submission for publication. Collaborator(s) will have 30 days from the date of receipt for review. Collaborator shall have the right to request that publication

CLOSED EFFECTIVE 06/04/2021

SWOG Authorized Document

be delayed for up to an additional 30 days in order to ensure that Collaborator's confidential and proprietary data, in addition to the Collaborator(s)'s intellectual property rights, are protected. Copies of abstracts must be provided to CTEP for forwarding to Collaborator(s) for courtesy review as soon as possible and preferably at least three (3) days prior to submission, but in any case, prior to presentation at the meeting or publication in the proceedings. Press releases and other media presentations must also be forwarded to CTEP prior to release. Copies of any manuscript, abstract and/or press release/media presentation should be sent to:

E-mail: ncicteppubbs@mail.nih.gov

The Regulatory Affairs Branch will then distribute them to the Collaborator(s). No publication, manuscript or other form of public disclosure shall contain any of the Collaborator's confidential/proprietary information.

Monitoring

This study will be monitored by the Clinical Data Update System (CDUS) Version 3.0. Cumulative CDUS data will be submitted quarterly to CTEP by electronic means. Reports are due January 31, April 30, July 31 and October 31.

Confidentiality

Please note that the information contained in this protocol is considered confidential and should not be used or shared beyond the purposes of completing protocol requirements until or unless additional permission is obtained.

16.1 Adverse Event Reporting Requirements

a. Purpose

Adverse event data collection and reporting, which are required as part of every clinical trial, are done to ensure the safety of patients enrolled in the studies as well as those who will enroll in future studies using similar agents. Adverse events are reported in a routine manner at scheduled times during a trial. (Directions for routine reporting are provided in Section 14.0.) Additionally, certain adverse events must be reported in an expedited manner to allow for more timely monitoring of patient safety and care. The following guidelines prescribe expedited adverse event reporting for this protocol. See also Appendix 18.1 for general and background information about expedited reporting.

b. Reporting method

This study requires that expedited adverse events be reported using the Cancer Therapy Evaluation Program Adverse Event Reporting System (CTEP-AERS). CTEP's guidelines for CTEP-AERS can be found at http://ctep.cancer.gov. A CTEP-AERS report must be submitted to the SWOG Operations Office electronically via the CTEP-AERS Web-based application located at: http://ctep.cancer.gov/protocolDevelopment/electronic_applications/adverse_events.htm.

CLOSED EFFECTIVE 06/04/2021

SWOG Authorized Document

c. When to report an event in an expedited manner

Some adverse events require 24-hour notification (refer to Table 16.1) via CTEP-AERS.

When the adverse event requires expedited reporting, submit the report within the number of calendar days of learning of the event, as specified in Table 16.1.

In the rare event when internet connectivity is disrupted a 24-hour notification is made to NCI by telephone at 301/897-7497. An electronic report MUST be submitted immediately upon re-establishment of internet connection.

Any supporting documentation requested by CTEP should be submitted in accordance with instructions provided by the CTEP-AERS system.

d. Other recipients of adverse event reports

The SWOG Operations Office will forward reports and documentation to the appropriate regulatory agencies and drug companies as required.

Adverse events determined to be reportable to the Institutional Review Board responsible for oversight of the patient must be reported according to local policy and procedures.

e. **Expedited reporting for investigational agents**

Expedited reporting is required if the patient has received at least one dose of the investigational agents as part of the trial. Reporting requirements are provided in Table 16.1. The investigational agent used in this study is pembrolizumab (MK-3475). If there is any question about the reportability of an adverse event or if on-line CTEP-AERS cannot be used, please telephone or email the SAE Specialist at the Operations Office, 210/614-8808 or adr@swog.org, before preparing the report.

CLOSED EFFECTIVE 06/04/2021

SWOG Authorized Document

Table 16.1:
Late Phase 2 and Phase 3 Studies: Expedited Reporting Requirements for Adverse Events that Occur on Studies under a CTEP IND within 30 Days of the Last Administration of the Investigational Agent/Intervention[1] pembrolizumab (MK-3475)

FDA REPORTING REQUIREMENTS FOR SERIOUS ADVERSE EVENTS (21 CFR Part 312)
NOTE: Investigators **MUST** immediately report to the sponsor (NCI) **ANY** Serious Adverse Events, whether or not they are considered related to the investigational agent(s)/intervention (21 CFR 312.64)

An adverse event is considered serious if it results in **ANY** of the following outcomes:

1) Death
2) A life-threatening adverse event
3) An adverse event that results in inpatient hospitalization or prolongation of existing hospitalization for ≥ 24 hours
4) A persistent or significant incapacity or substantial disruption of the ability to conduct normal life functions
5) A congenital anomaly/birth defect.
6) Important Medical Events (IME) that may not result in death, be life threatening, or require hospitalization may be considered serious when, based upon medical judgment, they may jeopardize the patient or patient and may require medical or surgical intervention to prevent one of the outcomes listed in this definition. (FDA, 21 CFR 312.32; ICH E2A and ICH E6).

ALL SERIOUS adverse events that meet the above criteria **MUST** be immediately reported to the NCI via CTEP-AERS within the timeframes detailed in the table below.

Hospitalization	Grade 1 Timeframes	Grade 2 Timeframes	Grade 3 Timeframes	Grade 4 & 5 Timeframes
Resulting in Hospitalization ≥ 24 hrs	10 Calendar Days			24-Hour 5 Calendar Days
Not resulting in Hospitalization ≥ 24 hrs	Not required		10 Calendar Days	

NOTE: Protocol specific exceptions to expedited reporting of serious adverse events are found in the Specific Protocol Exceptions to Expedited Reporting (SPEER) portion of the CAEPR or [Section 16.1f.]

Expedited AE reporting timelines are defined as:

- "24-Hour; 5 Calendar Days" - The AE must initially be reported via CTEP-AERS within 24 hours of learning of the AE, followed by a complete expedited report within 5 calendar days of the initial 24-hour report.
- "10 Calendar Days" - A complete expedited report on the AE must be submitted within 10 calendar days of learning of the AE.

[1]Serious adverse events that occur more than 30 days after the last administration of investigational agent/intervention and have an attribution of possible, probable, or definite require reporting as follows:

Expedited 24-hour notification followed by complete report within 5 calendar days for:

- All Grade 4, and Grade 5 AEs

Expedited 10 calendar day reports for:

- Grade 2 adverse events resulting in hospitalization or prolongation of hospitalization
- Grade 3 adverse events

May 5, 2011

CLOSED EFFECTIVE 06/04/2021

SWOG Authorized Document

f. **Additional Instructions or Exceptions to CTEP-AERS Expedited Reporting Requirements for Late Phase 2 and Phase 3 Studies Utilizing an Agent under a CTEP IND:**

1. Group-specific instructions

Submission of the on-line CTEP-AERS report plus any necessary amendments generally completes the reporting requirements. In addition, you may be asked to submit supporting clinical data to the Operations Offices in order to complete the evaluation of the event. If requested, the supporting data should be sent within **5 calendar days** by fax to 210-614-0006. Supporting clinical data submitted should include:

- Printed copy of the first page of the CTEP-AERS Report.
- Copies of clinical sourced documentation of the event.
- If applicable, and they have not yet been submitted to the SWOG Data Operations Center copies of Off Treatment Notice and/or Notice of Death.

2. The adverse events listed below also require expedited monitoring for this trial:

- Fever ≥ Grade 1 per CTCAE 4.0 accompanied by hypotension, dehydration requiring IV fluids
- Renal insufficiency (≥ Grade 2)
- ≥ Grade 3 chills in the absence of an obvious infectious cause

3. For both study arms, the adverse events listed below do **not** require expedited reporting via CTEP-AERS: none.

g. Reporting Secondary Malignancy, including AML/ALL/MDS

1. A secondary malignancy is a cancer caused by treatment for a previous malignancy (e.g., treatment with investigational agent/intervention, radiation or chemotherapy). A secondary malignancy is not considered a metastasis of the initial neoplasm.

CTEP requires all secondary malignancies that occur following treatment with an agent under an NCI IND to be reported via CTEP-AERS. Three options are available to describe the event.

- Leukemia secondary to oncology chemotherapy (e.g., Acute Myelocytic Leukemia [AML])

- Myelodysplastic syndrome (MDS)

- Treatment-related secondary malignancy

Any malignancy possibly related to cancer treatment (including AML/MDS) should also be reported via the routine reporting mechanisms outlined in each protocol.

Second Malignancy: A second malignancy is one unrelated to the treatment of a prior malignancy (and is NOT a metastasis from the initial malignancy). Second malignancies require ONLY routine reporting via CDUS unless otherwise specified.

CLOSED EFFECTIVE 06/04/2021

SWOG Authorized Document

For more information see:
http://ctep.cancer.gov/protocolDevelopment/electronic_applicatio2.

2. Supporting documentation should be submitted to CTEP in accordance with instructions provided by the CTEP-AERS system. A copy of the report and the following supporting documentation must also be submitted to SWOG Operations Office within 30 days:

- a copy of the pathology report confirming the AML/ALL /MDS diagnosis
- (if available) a copy of the cytogenetics report

SWOG
ATTN: SAE Program
4201 Medical Drive, Suite 250
San Antonio, Texas 78229

NOTE: If a patient has been enrolled in more than one NCI-sponsored study, the report must be submitted for the most recent trial.

h. Reporting Pregnancy, Fetal Death, and Death Neonatal

- **Pregnancy** Study participants who become pregnant while on study; that pregnancy should be reported in an expedited manner via CTEP-AERS as **Grade 3 "Pregnancy, puerperium and perinatal conditions – Other (pregnancy)"** under the **Pregnancy, puerperium and perinatal conditions** SOC.

  Additionally, the pregnancy outcome for patients on study should be reported via CTEP-AERS at the time the outcome becomes known, accompanied by the same Pregnancy Report Form used for the initial report.

- **Fetal Death** Fetal Death defined in CTCAE as "A disorder characterized by death in utero; failure of the product of conception to show evidence of respiration, heartbeat, or definite movement of a voluntary muscle after expulsion from the uterus, without possibility of resuscitation" should be reported expeditiously as **Grade 4 "pregnancy, puerperium and perinatal conditions – Other (pregnancy loss)" under the Pregnancy, puerperium and perinatal conditions** SOC.

- **Death Neonatal** Neonatal death, defined in CTCAE as "A disorder characterized by cessation of life occurring during the first 28 days of life" that is felt by the investigator to be at least possibly due to the investigational agent/intervention should be reported expeditiously.

  A neonatal death should be reported expeditiously as **Grade 4 "General disorders and administration – Other (neonatal loss)"** under the **General disorders and administration** SOC.

  *Fetal death and neonatal death should **NOT** be reported as a Grade 5 event. If reported as such, the* CTEP-AERS *interprets this as a death of the patient being treated.*

CLOSED EFFECTIVE 06/04/2021

SWOG Authorized Document

NOTE: When submitting CTEP-AERS reports for "Pregnancy, "Pregnancy loss", or "Neonatal loss", the Pregnancy Information Form should also be completed and faxed with any additional medical information to 301-230-0159. The potential risk of exposure of the fetus to the investigational agent(s) or chemotherapy agent(s) should be documented in the "Description of Event" section of the CTEP-AERS report.

The Pregnancy Information Form is available at http://ctep.cancer.gov/protocolDevelopment/adverse_effects.htmns/docs/aeguidelines.pdf.

CLOSED EFFECTIVE 06/04/2021

SWOG Authorized Document

17.0 BIBLIOGRAPHY

1 Champiat S, Ferte C, Lebel-Binay S, Eggermont A, Soria J. Exomics and immunogenics: Bridging mutational load and immune checkpoints efficacy. OncoImmunology 3(1):e27817, 2014.

2 Mar VJ, Wong SQ, Li J,Scolyer RA, McLean C, Papenfuss AT, Tothill RW, Kakavand H, Mann GJ, Thompson JF, Behren A, Cebon JS, Wokfe R, Kelly JW, Dobrovic A, McArthur GA. BRAF/NRAS wild-type melanomas have a high mutation load correlating with histologic and molecular signatures of UV damage. Clinical Cancer Research 19(17):4589-98, 2013.

3 Okazaki T, Chikuma S, Iwai Y, Fagarasan S, Honjo T, A rheostat for immune responses: the unique properties of PD-1 and their advantages for clinical application. Nat Immunol 14:1212-1218, 2013.

4 Keir ME, Butte MJ, Freeman GJ, Sharpe AH. PD-1 and its ligands in tolerance and immunity. Annu Rev Immunol 26:677-704, 2008.

5 Pardoll DM. The blockade of immune checkpoints in cancer immunotherapy. Nat Rev Cancer 12:252-64, 2012.

6 Keir ME, Butte MJ, Freeman GJ, Sharpe AH. PD-1 and its ligands in tolerance and immunity. Annu Rev Immunol 26:677-704, 2008.

7 Tumeh PC, Harview CL, Yearley JH, Shintaku IP, Taylor EJ, Robert L, Chmielowski B, Spasic M, Henry G, Ciobanu V, West AN, Carmona M, Kivork C, Seja E, Cherry G, Gutierrez AJ, Grogan TR, Mateus C, Tomasic G, Glaspy JA, Emerson RO, Robins H, Pierce RH, Elashoff DA, Robert C, Ribas A. PD-1 blockade induces responses by inhibiting adaptive immune resistance. Nature 27;515(7528):568-71, 2014.

8 Snyder A, Makarov V, Merghoub T, Yuan J, Zaretsky JM, Desrichard A, Walsh LA, Postow MA, Wong P, Ho TS, Hollmann TJ, Bruggeman C, Kannan K, Li Y, Elipenahli C, Liu C, Harbison CT, Wang L, Ribas A, Wolchok JD, Chan TA. Genetic basis for clinical response to CTLA-4 blockade in melanoma. N Engl J Med (4);371(23):2189-99, 2014.

9 Margaritescu I, Chirita AD. Desmoplastic melanoma-challenges in the diagnosis and management of a rare cutaneous tumor. Rom J Morphol Embryol 55 (3):947-952, 2014.

10 Margaritescu I, Chirita AD. Desmoplastic melanoma-challenges in the diagnosis and management of a rare cutaneous tumor. Rom J Morphol Embryol 55 (3):947-952, 2014.

11 Chen LL, Jaines N, Barker CA, Busam KJ, Marghoob AA. Desmoplastic melanoma: a review. J Am Acad Dermato 68(5):825-33, 2013.

12 Okazaki T, Chikuma S, Iwai Y, Fagarasan S, Honjo T, A rheostat for immune responses: the unique properties of PD-1 and their advantages for clinical application. Nat Immunol 14:1212-1218, 2013.

13 Keir ME, Butte MJ, Freeman GJ, Sharpe AH. PD-1 and its ligands in tolerance and immunity. Annu Rev Immunol 26:677-704, 2008.

14 Pardoll DM. The blockade of immune checkpoints in cancer immunotherapy. Nat Rev Cancer 12:252-64, 2012.

CLOSED EFFECTIVE 06/04/2021

SWOG Authorized Document

18.0 APPENDIX

18.1 Translational Medicine Details

18.2 Specimen Banking Instructions for the UCLA Lab #224: The Ribas Laboratory

18.3 Specimen Banking Instructions for the SWOG Biospecimen Bank

CLOSED EFFECTIVE 06/04/2021

18.1 Translational Medicine Details

Data storage and computational infrastructure: To optimally perform computational analysis on the large amount of genomic and imaging data to be generated from this study, it is expected that appropriate cloud computing infrastructure with the necessary analysis pipeline is required for the storage and computation of these data. One such computational infrastructure and resource available for this study is through the collaboration with the Parker Institute for Cancer Immunotherapy.

Whole exome sequencing to analyze mutational load: To test whether there is a correlation of tumor mutational load and response to PD-1 antibody therapy, DNA will be isolated from thick slides of FFPE tissues and *PBMC via commercial kit (i.e. Qiagen AllPrep DNA/RNA FFPE kit, cat 80234). Whole exome sequencing (WES), including probe-based exon capture, library preparation, and high throughput 100bp paired-end sequencing on the Illumina HiSeq2500 platform will be performed at the UCLA Immunogenetics core laboratory (CLIA approved lab). DNA from the same patient's PBMC (preferably) or other non-cancerous tissue will be isolated and sequenced for baseline comparison and differentiation of germline SNPs from true somatic mutations. Read mapping and data preprocessing will be performed according to the Broad Institute Best Practices Guidelines for the Genome Analysis Toolkit (version 3), and mutation detection will require a consensus call among several published programs (Varscan2, MuTect, GATK HaplotypeCaller). Mutational load will be quantified as the number of nonsynonymous somatic mutations per MB.

Quantitative analysis of T cell infiltration and circulating tumor DNA (ctDNA) profile: To analyze whether baseline CD8+ cell infiltration and ctDNA profile are correlated with response to anti-PD1 therapy, and whether PD-1 blockade can increase CD8+ cell infiltration in DM patients, slides taken from standard formalin-fixed paraffin-embedded (FFPE) tissue blocks obtained from baseline, 4 week (on treatment), and 9 week biopsies will be stained by quantitative multiplexed immunohistochemistry (IHC) for, at the minimum, S100 and CD8 to determine the presence of CD8+ T cells inside tumors and in the invasive margin. ctDNA profiling will be performed on plasma processed from patient baseline blood sample to determine the amount or fraction of ctDNA corresponding to the genetic profile of the patient tumor biopsies. Slides will be analyzed using quantitative digital pathology following published methods, such as described by Tumeh et al. and Angelo et al.. *(1, 2)* This analysis will provide a predictive score for response or lack of response to pembrolizumab based on the baseline CD8+ T cell infiltration or ctDNA profile.

<u>Method</u>: Slides will be stained with, at the minimum, S100 and CD8 (one slide per stain, or multiple stain per slide as appropriate for chosen quantitative multiplexed IHC technique) at a core facility with the instrumentation, such as the UCLA Anatomic Pathology IHC Laboratory (CLIA approved lab). Immunostaining will be performed using staining procedures appropriate for the specific quantitative multiplexed IHC technique and validated by the Translational Medicine team, for example, Leica Bond III autostainers using Leica Bond ancillary reagents and REFINE polymer DAB detection system (Leica DS9800). Antibodies used will include rabbit polyclonal S100 (DAKO, 1/1,000 dilution, low pH retrieval), mouse anti-CD8 clone C8/144B (DAKO, 1/100, low PH retrieval), and all staining will be performed on Dako autostainers, and visualized with DAB. Slides will be baked for 60 minutes at 60°C. Procedure involves dewaxing, heat-retrieved in Bond ER1(pH6) buffer for 20 minutes, and sequential incubations with primary antibody, post-primary reagent, and polymer for 15 minutes, 8 minutes, and 8 minutes, respectively, and subsequent DAB chromogen application and Hematoxylin counterstaining. All stained slides will be evaluated in a blinded fashion by one dermatopathologist and one investigator trained to identify the features of melanoma, and the presence of CD8 within the tumor parenchyma (tumor) and the connective tissue surrounding the tumor (invasive margin) will be examined.

CLOSED EFFECTIVE 06/04/2021

SWOG Authorized Document

All slides will be scanned at an absolute magnification of X200 (resolution of 0.5 mm/pixel). An algorithm was designed based on pattern recognition that quantified immune cells within S100-positive areas (tumor) and S100-negative areas (invasive margin). Image analysis based on RGB (red, green, blue) spectra will be used to detect all cells by counterstaining with haemotoxylin (blue), and DAB or fast red. The algorithm calculated the density (cells mm^{-2}) and percentage cellularity (% positive cells/all nucleated cells) using Indica Labs Halo platform.

Analysis of TCR clonality in tumors: To answer the question whether the T cell response is targeting specific tumor antigen (mono-clonal) or a non-specific process (poly-clonal) in the tumors, we will analyze TCR clonality by deep sequencing the TCR Vβ CDR region using the ImmunoSeq assay from Adaptive Biotech as described. *(3)* From thick slides of FFPE tissues taken at baseline, 4 weeks, and 9 weeks, we will isolate DNA and submit it for TCR Vβ sequencing. This analysis will provide information about the clonality or diversity of T cells infiltrating tumors in DM samples. DNA analysis will be performed by the UCLA Immunogenetics core laboratory (CLIA approved lab).

DNA will be isolated from FFPE thick cuts followed by extraction using a DNeasy kit (Qiagen). Melanin will be removed from visibly pigmented melanoma samples using a PCR Inhibitor Removal kit (ZymoResearch). TCR Vβ CDR3 regions will be amplified and sequenced using the survey ImmunoSeq assay in a multiplexed PCR method using 45 forward primers specific to TCR Vβ gene segments and 13 reverse primers specific to TCR Vβ gene segments (Adaptive Biotechnologies). Reads of length 87bp will be obtained using the Illumina HiSeq System. For each sample, Shannon entropy will be calculated on the clonal abundance of all productive TCR sequences in the data set. Shannon entropy will be normalized to the range by dividing Shannon entropy by the logarithm of the number of unique productive TCR sequences in the data set. This normalized entropy value will be then inverted (12 normalized entropy) to produce our clonality metric.

Analysis of adaptive immune resistance mechanism in tumors: Slides taken from standard FFPE tissue blocks obtained from baseline, the on treatment biopsy (week 4) and the week 9 biopsy/resection will be stained by quantitative multiplexed immunohistochemistry (IHC) for a set of multiple immune-relevant biomarkers, including PD-L1 (SP142, Spring Bioscience), to evaluate whether PD-L1 can be induced by PD-1 blockade, the patient's immune response, and which is correlated with response. Slides will be analyzed using quantitative digital pathology following the methods described for Quantitative analysis of T cell infiltration. Analysis will be performed at core facilities with the relevant instrumentations and infrastructures, such as the UCLA Anatomic Pathology IHC Laboratory (CLIA approved lab).

<u>Method:</u>

1. Bake slides for 1 hour at 65°C.

2. Deparaffinize slides in xylene (3 changes) and rehydrate through graded ethanol to deonized-H2O.

3. Perform antigen retrieval in pressure cooker for 5 minutes with Tris-EDTA pH9 buffer (Leica ER2). Cool for 15 minutes at room temperature and rinse in DI-water.

Perform IHC of samples using staining procedures appropriate for the specific quantitative multiplexed IHC technique and validated by the Translational Medicine team, for example, Leica Bond III autostainer with Refine Polymer Detection (Leica DS9800); autostainer programmed for primary antibody at 1/200 dilution in daVinci Green Diluent (Biocare Medical) for 60 minutes, Polymer for 15 minutes, Peroxidase block for 5 minutes, DAB for

CLOSED EFFECTIVE 06/04/2021

SWOG Authorized Document

10 minutes, and offline manual steps 0.5% cupric sulfate for 10 minutes and Hematoxylin for 5 minutes. Usual Bond washes between steps. Use Bond Refine Polymer Detection kit (DS9800), which includes reagents for all steps after primary antibody, except for cupric sulfate and hematoxylin counterstain.

References for 18.1

1 Tumeh PC, Harview CL, Yearley JH, Shintaku IP, Taylor EJ, Robert L, Chmielowski B, Spasic M, Henry G, Ciobanu V, West AN, Carmona M, Kivork C, Seja E, Cherry G, Gutierrez AJ, Grogan TR, Mateus C, Tomasic G, Glaspy JA, Emerson RO, Robins H, Pierce RH, Elashoff DA, Robert C, Ribas A. PD-1 blockade induces responses by inhibiting adaptive immune resistance. Nature 27;515(7528):568-71, 2014

2 Angelo M, Bendall SC, Finck R, Hale MB, Hitzman C, Borowsky AD, Levenson RM, Lowe JB, Liu SD, Zhao S, Natkunam Y, Nolan GP. Multiplexed ion beam imaging of human breast tumors. Nat Med. 2014 Apr;20(4):436-42. doi: 10.1038/nm.3488. Epub 2014 Mar 2. PMID: 24584119; PMCID: PMC4110905.

3 Tumeh PC, Harview CL, Yearley JH, Shintaku IP, Taylor EJ, Robert L, Chmielowski B, Spasic M, Henry G, Ciobanu V, West AN, Carmona M, Kivork C, Seja E, Cherry G, Gutierrez AJ, Grogan TR, Mateus C, Tomasic G, Glaspy JA, Emerson RO, Robins H, Pierce RH, Elashoff DA, Robert C, Ribas A. PD-1 blockade induces responses by inhibiting adaptive immune resistance. Nature 27;515(7528):568-71, 2014

CLOSED EFFECTIVE 06/04/2021

SWOG Authorized Document

18.2 Specimen Banking Instructions for the UCLA Lab #224: The Ribas Laboratory

The UCLA Lab #224, The Ribas Laboratory, will receive formalin-fixed, paraffin-embedded (FFPE) tumor tissue as either blocks or unstained slides as noted in Section 15.1.

Upon receipt, the Ribas Laboratory will accession, barcode, and bank all FFPE specimens at room temperature until ready for translational medicine as detailed in Section 18.1.

The UCLA lab #224 will also receive twenty mL fresh whole blood in vacutainer tubes with EDTA coating at ambient temperature approximately as noted in Section 15.1. Upon receipt, blood will be processed per translational medicine details in Section 18.1.

CLOSED EFFECTIVE 06/04/2021

SWOG Authorized Document

18.3 Specimen Banking Instructions for the SWOG Biospecimen Bank

The SWOG Bank will receive stained slides from formalin-fixed, paraffin-embedded (FFPE) tumor tissue as noted in Section 15.2. Upon receipt, the Bank will accession, barcode, and bank all FFPE tissue slides at room temperature.

For each case, one H&E stained slide and all immunostained slides will be scanned at 40X magnification to Dr. Wada and Dr. Plaza for digital pathology review in batches throughout the study. A redacted pathology report will accompany the digital whole slide images.

CLOSED EFFECTIVE 06/04/2021

SWOG Authorized Document
