# Supplementary material for: Neoadjuvant PD-1 blockade in surgically resectable desmoplastic melanoma: cohort A of the phase 2 SWOG S1512 trial
Source: Nat Cancer. 2026 Jan 29;7(2):272–82. doi: 10.1038/s43018-025-01113-y (PMC12948668; doi:10.1038/s43018-025-01113-y)
Supplement: Supplementary file 2 — Reporting Summary [file 43018_2025_1113_MOESM2_ESM.pdf]

nature portfolio

Corresponding author(s): Antoni Ribas

Last updated by author(s): Dec 16, 2025

Reporting Summary

Nature Portfolio wishes to improve the reproducibility of the work that we publish. This form provides structure for consistency and transparency in reporting. For further information on Nature Portfolio policies, see our Editorial Policies and the Editorial Policy Checklist.

Statistics

For all statistical analyses, confirm that the following items are present in the figure legend, table legend, main text, or Methods section.

n/a	Confirmed	
☐	☒	The exact sample size (*n*) for each experimental group/condition, given as a discrete number and unit of measurement
☐	☒	A statement on whether measurements were taken from distinct samples or whether the same sample was measured repeatedly
☐	☒	The statistical test(s) used AND whether they are one- or two-sided<br>*Only common tests should be described solely by name; describe more complex techniques in the Methods section.*
☒	☐	A description of all covariates tested
☐	☒	A description of any assumptions or corrections, such as tests of normality and adjustment for multiple comparisons
☐	☒	A full description of the statistical parameters including central tendency (e.g. means) or other basic estimates (e.g. regression coefficient) AND variation (e.g. standard deviation) or associated estimates of uncertainty (e.g. confidence intervals)
☐	☒	For null hypothesis testing, the test statistic (e.g. F, t, r) with confidence intervals, effect sizes, degrees of freedom and P value noted<br>*Give P values as exact values whenever suitable.*
☒	☐	For Bayesian analysis, information on the choice of priors and Markov chain Monte Carlo settings
☒	☐	For hierarchical and complex designs, identification of the appropriate level for tests and full reporting of outcomes
☐	☒	Estimates of effect sizes (e.g. Cohen's d, Pearson's r), indicating how they were calculated

Our web collection on statistics for biologists contains articles on many of the points above.

Software and code

Policy information about availability of computer code

Data collection	Clinical data from sites reported through iMedidate Rave; transferred to the SWOG SQL database; exported for analysis in R (v4.2.0).
Data analysis	All analyses were performed using SAS 9.4 (SAS Institute Inc.) and R v4.3.1 (The R Foundation for Statistical Computing).

For manuscripts utilizing custom algorithms or software that are central to the research but not yet described in published literature, software must be made available to editors and reviewers. We strongly encourage code deposition in a community repository (e.g. GitHub). See the Nature Portfolio guidelines for submitting code & software for further information.

Data

Policy information about availability of data

All manuscripts must include a data availability statement. This statement should provide the following information, where applicable:

- Accession codes, unique identifiers, or web links for publicly available datasets
- A description of any restrictions on data availability
- For clinical datasets or third party data, please ensure that the statement adheres to our policy

SWOG makes all research data available externally to investigators and pharmaceutical companies, in accordance with the policies of the National Institutes of

Health (NIH) and National Cancer Institute (NCI). All data to reproduce the analyses presented in this article are available upon request from SWOG in accordance with SWOG's data sharing policy and procs: https://www.swog.org/sites/default/files/docs/2019-12/Policy43_0.pdf.
The protocol (including statistical analysis plan in Section 11 of the protocol) and informed consent are Supplementary Materials. Whole exome sequencing data is available with an authorized access upon registration and approval via dbGaP at accession https://dbgap.ncbi.nlm.nih.gov/beta/study/phs004123.v1.p1. The remaining data are available within the Article, Supplementary Information and/or Source Data file. Source data are provided with this paper

Human research participants

Policy information about studies involving human research participants and Sex and Gender in Research.

Reporting on sex and gender	Patients were screened and enrolled on this study irrespective of their sex/gender. Any data regarding a patient's sex and gender was collected by the clinical trial groups at each site. Sex- or gender-based subgroup analysis are reported in Table 1 and Extended Data Table 1.
Population characteristics	Provided in Extended Data Table 1.
Recruitment	Patients were recruited across 10 clinical investigational sites. Given that this was an NCI-funded US cooperative group trial, the sites were limited to the United States of America. There were no biases introduced and patients were screened on a first-come first-serve basis based on meeting the protocol inclusion-exclusion criteria. No protocol waivers were allowed on this study.
Ethics oversight	The trial was conducted in accordance with the principles of the Declaration of Helsinki. The trial protocol and statistical analysis plan were designed in a collaboration between the SWOG and CTEP investigators. The protocol was approved by the Cancer Therapy Evaluation Program (CTEP) Central Institutional Review Board (CIRB)and institutional review boards from each of the 10 clinical sites enrolling patients to cohort A of S1512.

Note that full information on the approval of the study protocol must also be provided in the manuscript.

Field-specific reporting

Please select the one below that is the best fit for your research. If you are not sure, read the appropriate sections before making your selection.

☒ Life sciences ☐ Behavioural & social sciences ☐ Ecological, evolutionary & environmental sciences

For a reference copy of the document with all sections, see nature.com/documents/nr-reporting-summary-flat.pdf

Life sciences study design

All studies must disclose on these points even when the disclosure is negative.

Sample size	The primary endpoint was the rate of pathologic complete response (pCR). The sample size (n=25) was based on a single stage design with 90% power to rule out a pCR rate of 5% at the 3.4% level, if the true pCR rate was 25%. The observation of four out of 25 cases with pCR would be considered evidence that the treatment warrants further study, provided other factors such as toxicity and overall survival also appear favorable. Enrollment of 30 patients was estimated to allow for at least 25 evaluable patients. All patients who receive a single dose of study treatment and met eligibility criteria were considered evaluable for the primary endpoint.
Data exclusions	28 of 30 enrolled patients were included in the analysis. One patient refused protocol therapy and withdrew consent, and one patient was deemed ineligible after a review of the pathology report indicated their disease was not consistent with desmoplastic melanoma.
Replication	N/A - phase 2 clinical trial
Randomization	Not randomized.
Blinding	Study are was no blinded. No placebo was given and so no blinding was possible, per standard with many oncology studies.

Reporting for specific materials, systems and methods

We require information from authors about some types of materials, experimental systems and methods used in many studies. Here, indicate whether each material, system or method listed is relevant to your study. If you are not sure if a list item applies to your research, read the appropriate section before selecting a response.

Materials & experimental systems

n/a	Involved in the study	
☐	☒	Antibodies
☒	☐	Eukaryotic cell lines
☒	☐	Palaeontology and archaeology
☒	☐	Animals and other organisms
☐	☒	Clinical data
☒	☐	Dual use research of concern

Methods

n/a	Involved in the study	
☒	☐	ChIP-seq
☒	☐	Flow cytometry
☒	☐	MRI-based neuroimaging

Antibodies

Antibodies used

The therapeutic antibody used for treatment of patients within the clinical trial was pembrolizumab (Keytruda(R)), provided by Merck through NCI/CTEP.

Validation

Describe the validation of each primary antibody for the species and application, noting any validation statements on the manufacturer's website, relevant citations, antibody profiles in online databases, or data provided in the manuscript.

Clinical data

Policy information about clinical studies

All manuscripts should comply with the ICMJE guidelines for publication of clinical research and a completed CONSORT checklist must be included with all submissions.

Clinical trial registration

NCT02775851

Study protocol

The study protocol is provided in the Supplemental Information Files.

Data collection

Data was collected at individual sites between July 2017 and May 2024. Data was submitted online through the iMedidate Rave platform, which is uploaded daily into the SWOG SQL database. Enrolling sites were responsible for uploading data for participants enrolled on the trial. Each site followed their site-specific rules for data collection and submission, following the timelines provided in the protocol.

Outcomes

A one-sided exact binomial test using the method of Clopper and Pearson was used to test the pCR rate against the null hypothesis. Binary proportions are summarized along with 95% confidence intervals. The method of Kaplan-Meier was used to estimate the distributions of relapse-free survival and overall survival, and the log-log method was used to estimate the corresponding confidence intervals for survival at three years. Melanoma-specific survival was calculated as 1-CIF, where CIF is the cumulative incidence of melanoma-specific deaths estimated non-parametrically using the method of Nelson-Aalen. Death from other causes were treated as competing risks. Melanoma-specific survival confidence interval for survival at three years was calculated using the method recommended by Pintilie 20. All analyses were performed using SAS 9.4 (SAS Institute Inc.) and R v4.3.1 (The R Foundation for Statistical Computing).
